# Expanding the Chemistry of Pentafluorophenyl-N-Confused Porphyrin: Diketonate Substitution and Derivatizations at the External 3-C Position of the Inverted Pyrrole Ring

**DOI:** 10.1021/acsorginorgau.4c00065

**Published:** 2024-10-01

**Authors:** Bhakyaraj Kasi, Belarani Ojha, Wen-Feng Liaw, Chen-Hsiung Hung

**Affiliations:** † Molecular Science and Technology Program, Taiwan International Graduate Program, Academia Sinica, Taipei 115201, Taiwan; ‡ Department of Chemistry, National Tsing Hua University, Hsinchu 300044, Taiwan; § Institute of Chemistry, Academia Sinica, Nankang, Taipei 115201, Taiwan

**Keywords:** N-confused porphyrins, acyl cleavage degradation, ring fusion, BF_2_ chelation, HF elimination

## Abstract

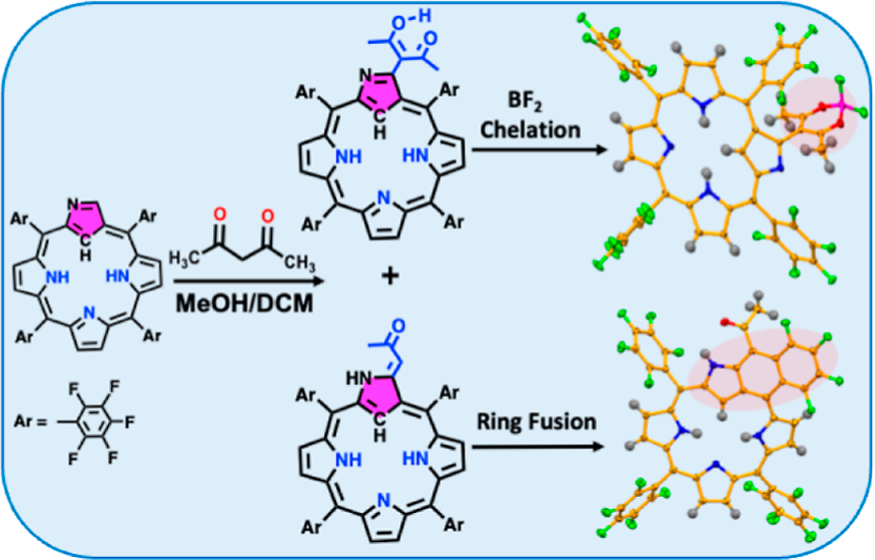

In this study, we synthesized two new 3-C-substituted pentafluorophenyl-N-confused porphyrins (PFNCPs), one with acetylacetonate (PFNCP-acac, **2a**) and the other with ylidene-2-propanone (PFNCP-ac, **3a**), through a one-pot reaction in the absence of a catalyst. Under mild acidic and heating conditions, the acac-substituted compound underwent acyl cleavage degradation, yielding ac-substituted product **3a**. Subsequent chelation of the acac-substituted PFNCP with BF_2_ resulted in a boron diketonate derivative, PFNCP-acacBF_2_ (**4**). Additionally, an electrocyclic reaction of the ac-substituted PFNCP **3a**, without a catalyst, produced a tricyclic fused [6,6,5]-TF-PFNCP (**5**). This tricyclic product could also be obtained directly from PFNCP-acac **2a** under heating conditions. The absorption spectra revealed that acac- and ac-substituted macrocycles exhibit either a single or split Soret band, respectively, in the 400–550 nm range, along with multiple *Q* bands spanning the 580–690 nm region. While BF_2_ derivatization caused a slight red shift in the absorption spectra, the [6,6,5]-tricyclic fused NCP demonstrated a significant red shift. All newly synthesized compounds were characterized by using single-crystal X-ray structures, ^1^H NMR spectroscopy, and mass spectrometry. Density functional theory (DFT) studies were conducted to elucidate the photophysical properties of these macrocycles.

## Introduction

N-Confused porphyrins (NCPs)^1,2^ and their carbaporphyrinoid counterparts have markedly advanced the field of porphyrin analogues.^3^ These compounds exhibit a distinctive core structure featuring a pyrrolic joint connecting the macrocycle via an α- and a β-pyrrolic carbon, resulting in a unique three-carbon, one-nitrogen core.^4^ The presence of this three-carbon, one-nitrogen core, coupled with reactive peripheral nitrogen and carbon atoms, significantly enhances their chemical diversity.^5^ The remarkable electronic, optical, and physical properties of NCPs, stemming from their dynamic tautomeric states and aromatic nature, facilitate a variety of applications. These include bioinorganic modeling, photodynamic therapy,^6^ solar cell sensitization, molecular sensing,^7,8^ catalysis,^9−12^ and the development of materials for electronic devices.^13,14^

**Chart 1:**
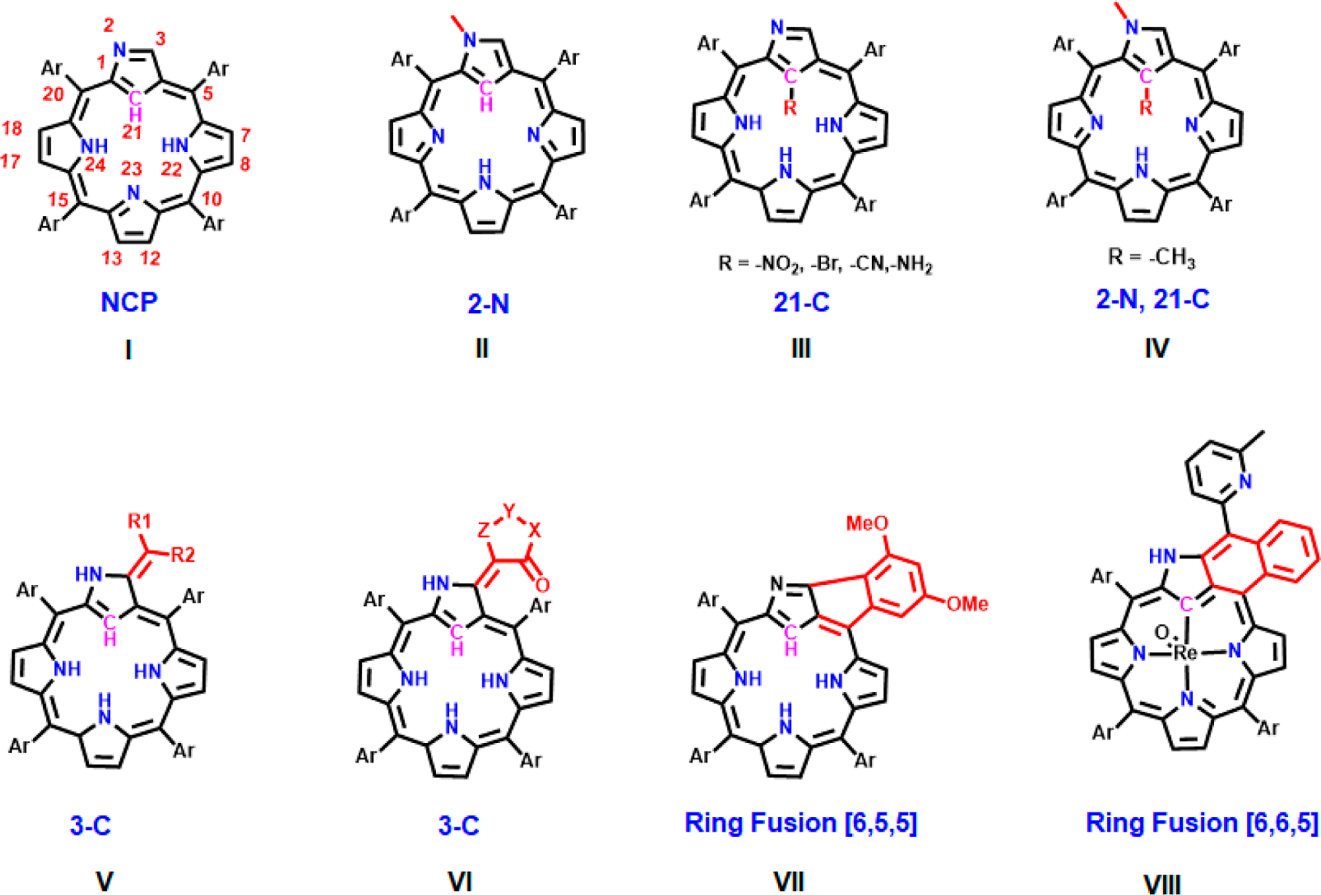
Structures of NCP Derivatives with Different Active Sites

NCP derivatives can be synthesized by targeting three primary active sites^15,16^ on the inverted pyrrole ring—specifically, the external nitrogen (2-N),^17−19^ the external carbon atom (3-C),^20−23^ and the internal carbon (21-C),^24,25^ as depicted in Chart 1 (refer to structure I for atom numbering). The external nitrogen at position 2-N in NCPs exhibits lower steric hindrance and higher nucleophilicity compared to the pyrrolic nitrogen atoms in the inner core of porphyrins.^15^ Alkylation of 2-N, as shown in Chart 1II, can be efficiently accomplished using an alkylation reagent and Cs_2_CO_3._^26^ Additionally, the reaction of the NiNCP complex with CH_3_I induces alkylation of the inner 21-C through oxidative addition and elimination processes.^27^ Subsequent treatment with a second equivalent of CH_3_I results in dual methylation at both 2-N and 21-C dual, as depicted in Chart 1IV.^28−30^

Theoretical calculations indicate that in Ag(III) NCP macrocycles, the outer C=N bond is highly electrophilic, making 3-C the primary site for imine-type nucleophilic substitution reactions^31^ (refer to Chart 1V,VI). Protonation or the presence of an electron-withdrawing group at 2-N enhances the electrophilic nature of 3-C. Various reactions, including amination,^32^ alkoxylation,^31^ cyanation,^33^ and C–C bond coupling,^34,35^ have been used to functionalize the 3-C position. For example, 3-ethoxy-substituted AgNCP was synthesized using NCP, CF_3_CO_2_Ag, and EtOH, with subsequent hydrolysis leading to a lactam ring-incorporated NCP.^36^ Additionally, reactions with active methylene precursors, like cyclohexane-1,3-dione, have yielded 3-C-substituted NCP derivatives efficiently.^37^

NCP also undergoes distinctive reactions involving the inverted pyrrole ring such as ring fusion and expansion. For instance, dual bromination at both 3-C and 21-C facilitates C–N bond formation between 3-C and adjacent pyrrolic nitrogen, resulting in N-fused porphyrin (NFP) formation through HBr elimination. Introducing a bridging unit, like a carbonyl or ethylene group, allows the preparation of fused ring NCPs.^38^ Moreover, exocyclic extended ring systems can be achieved by forming a C–C bond between 3-C and the *ortho*-carbon of the adjacent *meso*-3,5-dimethoxyphenyl substituent, leading to a [6,5,5]-tricyclic fused ring.^39^ The first [6,6,5]-tricyclic exocyclic ring-fused NCP complex was serendipitously isolated from a rhenium metalation reaction using 2,6-lutidine, where Re(I) activates the C–H bond in the lutidine’s methyl group.^40^ The incorporation of exocyclic rings is as in Chart 1VII,VIII results in a bathochromic shift and enhances the reversibility of redox processes due to the more extensive and rigid ring system.^41−47^

**Chart 2:**
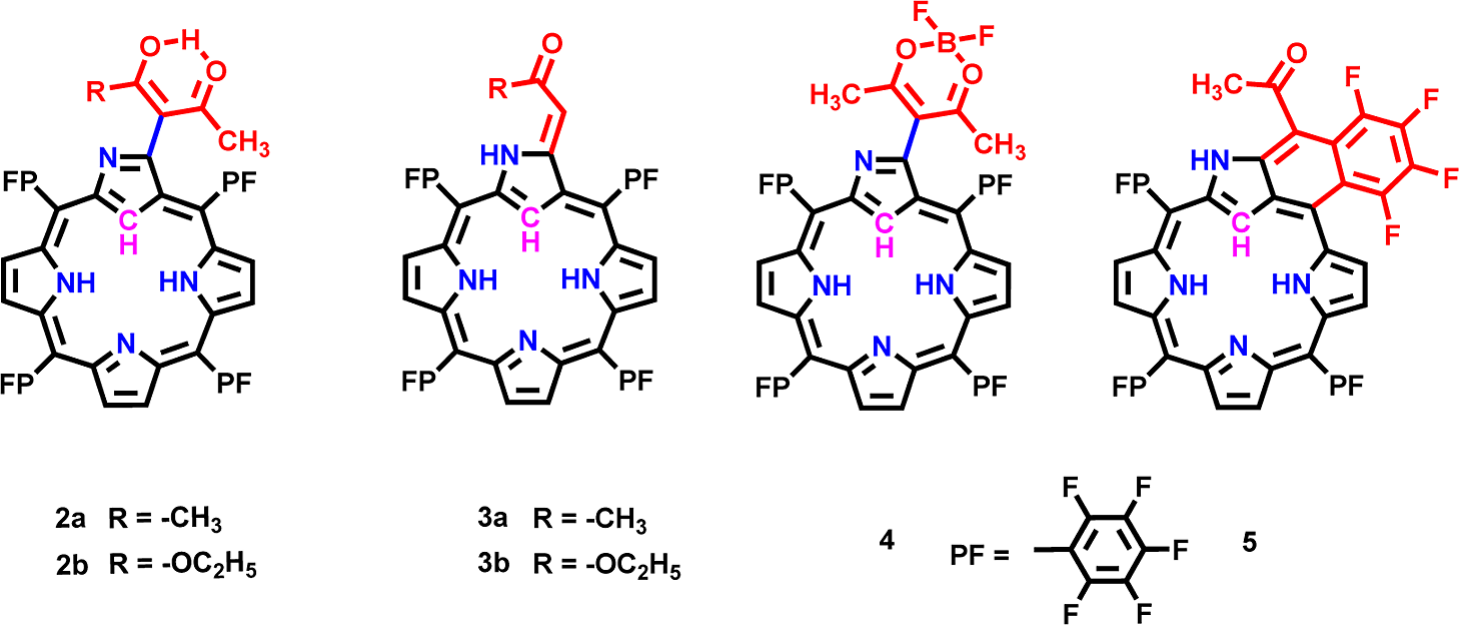
Structures of Various 3-C-Substituted PFNCP Derivatives **2–5**

In this work, we describe the synthesis and characterization of 3-C acetylacetonate-substituted PFNCP-acac **2a** (Chart 2), which provides a general method for producing 3-C diketonate-substituted derivatives. When treated with BF_3_·OEt_2_ in basic conditions, it yielded the difluoroborate-chelated PFNCP-acacBF_2_**4**. Under an acidic environment, the acetylacetonate group of **2a** undergoes an acyl cleavage degradation reaction, yielding a 3-C methyl vinyl ketone-derived PFNCP-ac **3a**. Subsequently, a catalyst-free electrocyclic reaction in the free-base state facilitates the transformation into [6,6,5]-tricyclic fused [6,6,5]-TF-PFNCP 5. Notably, the presence of a *meso*-pentafluorophenyl group near the 3-C position significantly influences these reactions, likely due to an inductive effect facilitating interactions between the 3-C and the *ortho* C–F unit. Additionally, another variant of 3-C-substituted PFNCP was synthesized by reacting PFNCP with ethyl acetoacetate, resulting in a 3-ketoester compound (PFNCP-eacac, **2b**) and its corresponding acyl cleavage degradation product (PFNCP-eac, **3b**).

## Results and Discussion

### Synthesis

**Scheme 1:**
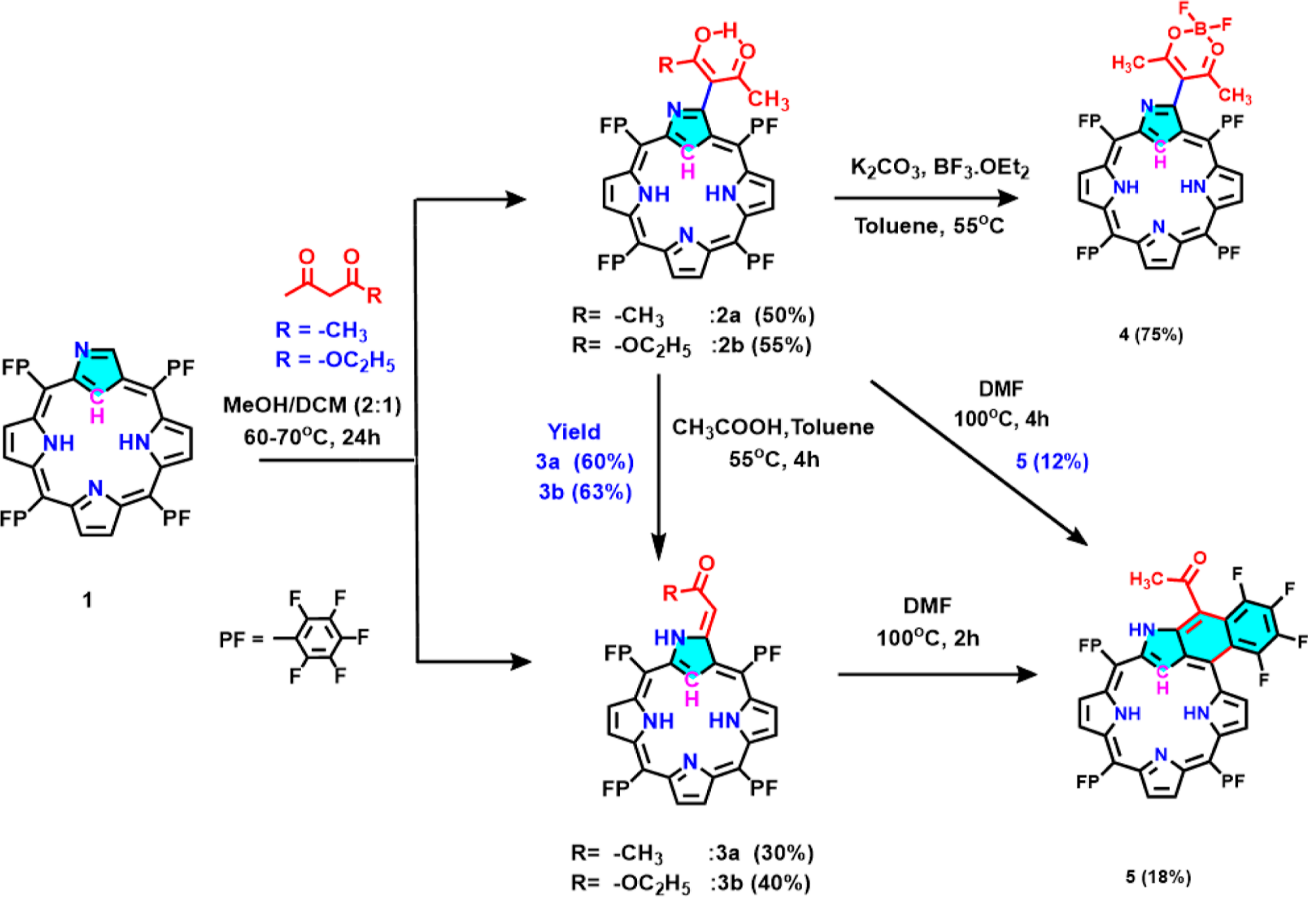
Synthesis of Compounds **1–5**

Novel derivatives of PFNCP, specifically PFNCP-acac **2a** with an acetylacetonate group and PFNCP-ac **3a** with a 3-ylidene-2-propanone substitution at the 3-C position of the inverted pyrrole ring, were synthesized under mild reaction conditions without any catalyst, as depicted in Scheme 1. PFNCP **1** was initially prepared via the condensation reaction starting from 2,3,4,5,6-pentafluorobenzaldehyde and pyrrole, following the established Lindsey’s NCP-optimized preparation conditions.^48^ Compound **1**, when reacted with acetylacetonate in CH_3_OH/CH_2_Cl_2_ (2:1) at 60–70 °C for 24 h, afforded compounds **2a** and **3a** with 50% and 30% yields, respectively, after silica gel column chromatography. In similar conditions, treating **1** with ethyl acetoacetate led to the formation of another type of 3-C-substituted PFNCP, PFNCP-eacac **2b** and PFNCP-eac **3b**. The acetylacetonate-substituted **2a** was used as a precursor for the preparations of other PFNCP derivatives. Reacting **2a** with acetic acid in toluene under mild heating conditions led to acyl cleavage in the acetylacetonate group, producing compound **3a** with a 60% yield.^49^ Notably, the acetylacetonate unit demonstrated strong chelation affinity toward difluoroborate, and its reaction with BF_3_·OEt_2_ in basic conditions yielded the difluoroborate-chelated PFNCP-acacBF_2_**4** with a 75% yield.

The formation of a [6,6,5]-tricyclic fused ring structure in PFNCP was initially perceived from a metalation reaction of compound **2a** with [VO(acac)_2_]. Since using **3a** as a precursor for the synthesis of tricyclic fused ring product [6,6,5]-TF-PFNCP (**5**) only requires HF elimination, **3a** was refluxed in dimethylformamide (DMF), acting both as a solvent and a base, for 2 h, isolating **5** with an 18% yield. Similarly, refluxing compound **2a** in DMF for 4 h also resulted in the formation of compound 5 with a 12% yield. The 3-ketoester NCP **2b** also underwent an acyl cleavage reaction in the presence of acid, leading to the formation of acrylate **3b** (63% yield). All new compounds (**2–5**) are freely soluble in organic solvents and were thoroughly characterized by UV–vis spectroscopy; mass spectrometry; ^1^H, ^19^F, ^11^B, and ^13^C NMR; and single-crystal X-ray diffraction (XRD) analyses.

### Spectroscopic Characterizations of PFNCP Derivatives

**Figure 1:**
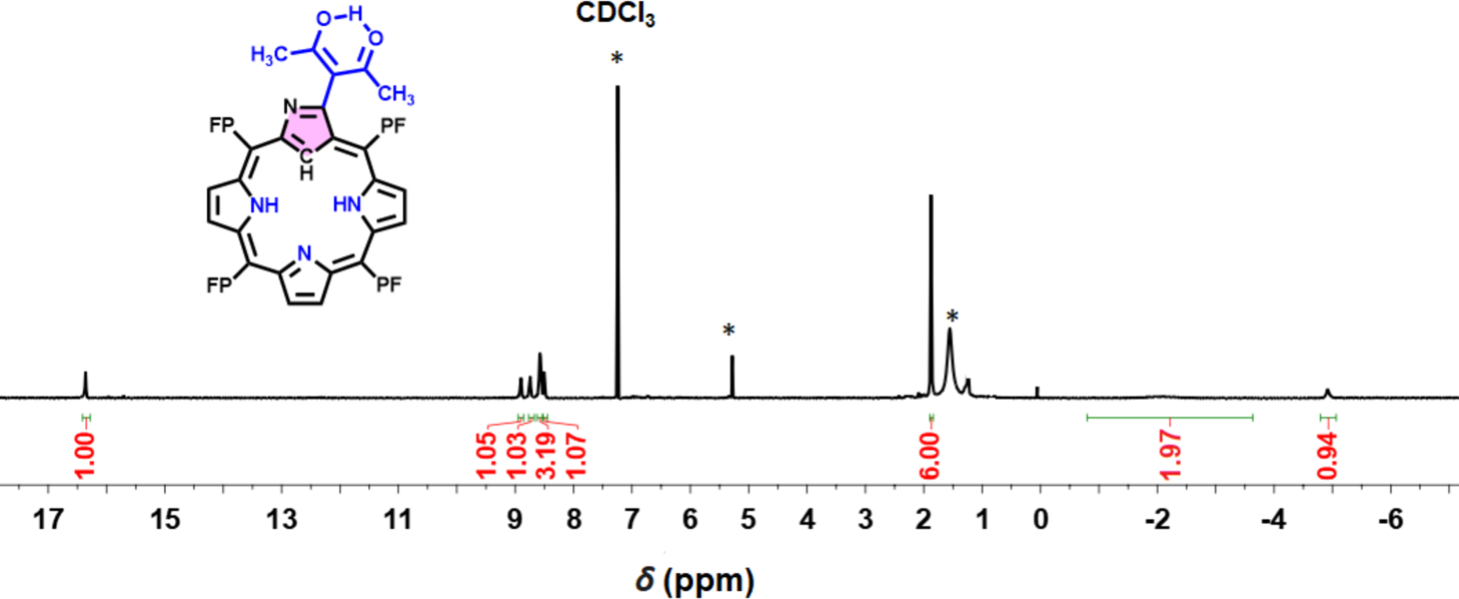
^1^H NMR (400 MHz) spectra of compound **2a** recorded in CDCl_3_ (* stands for solvent impurity).

The resonances observed in the ^1^H NMR spectroscopy of compounds **2–5** in CDCl_3_ provide clear structural insights, simplified by the absence of *meso*-phenyl protons. The ^1^H NMR spectrum of compound **2a** is depicted in Figure 1, while the spectra of all other new compounds are presented in Figures S2, S4, S9, S12, S16, and S19. ^1^H NMR spectra of macrocycle **2a** and its BF_2_-chelated derivative **4** are characterized by six β-pyrrolic protons (a–f) on PFNCP, appearing in the range of 8.52–8.98 ppm. Additionally, a broad resonance from two inner pyrrolic NHs (g,h) was observed between −2.05 and −2.41 ppm, along with a sharp inner –CH (i) of the inverted pyrrole at −4.98 ppm. A notable feature in **2a** is the enolic proton (l) of the acac unit, appearing as a singlet at 16.36 ppm, which is absent in NCP 1 (Figure. S7). This unusual downfield shift is attributed to both hydrogen bonding with the carbonyl oxygen atom and the anisotropic effect. The two methyl groups of the acac unit (j,k) are identified as a singlet at 1.88 ppm. The formation of the boron diketonate complex **4** through BF_2_ chelation is confirmed by the resonances in the ^11^B and ^19^F NMR (Figures S21 and S22). The absence of the enolic hydroxyl proton signal in the downfield region above 10 ppm in the ^1^H NMR spectrum of compound **4** (Figures S19 and S23) further supports the insertion of the BF_2_ moiety. Notably, the similar patterns of proton resonances to PFNCP (1) suggest that compounds **2a** and **4** predominately exist in the 3H tautomeric form.^48^ It also shows that the introduction of the acac unit or BF_2_ chelation does not significantly alter the electronic structures of the PFNCP core due to the limited conjugation interaction between the acac π-electrons and the porphyrinic conjugation system.

**Figure 2:**
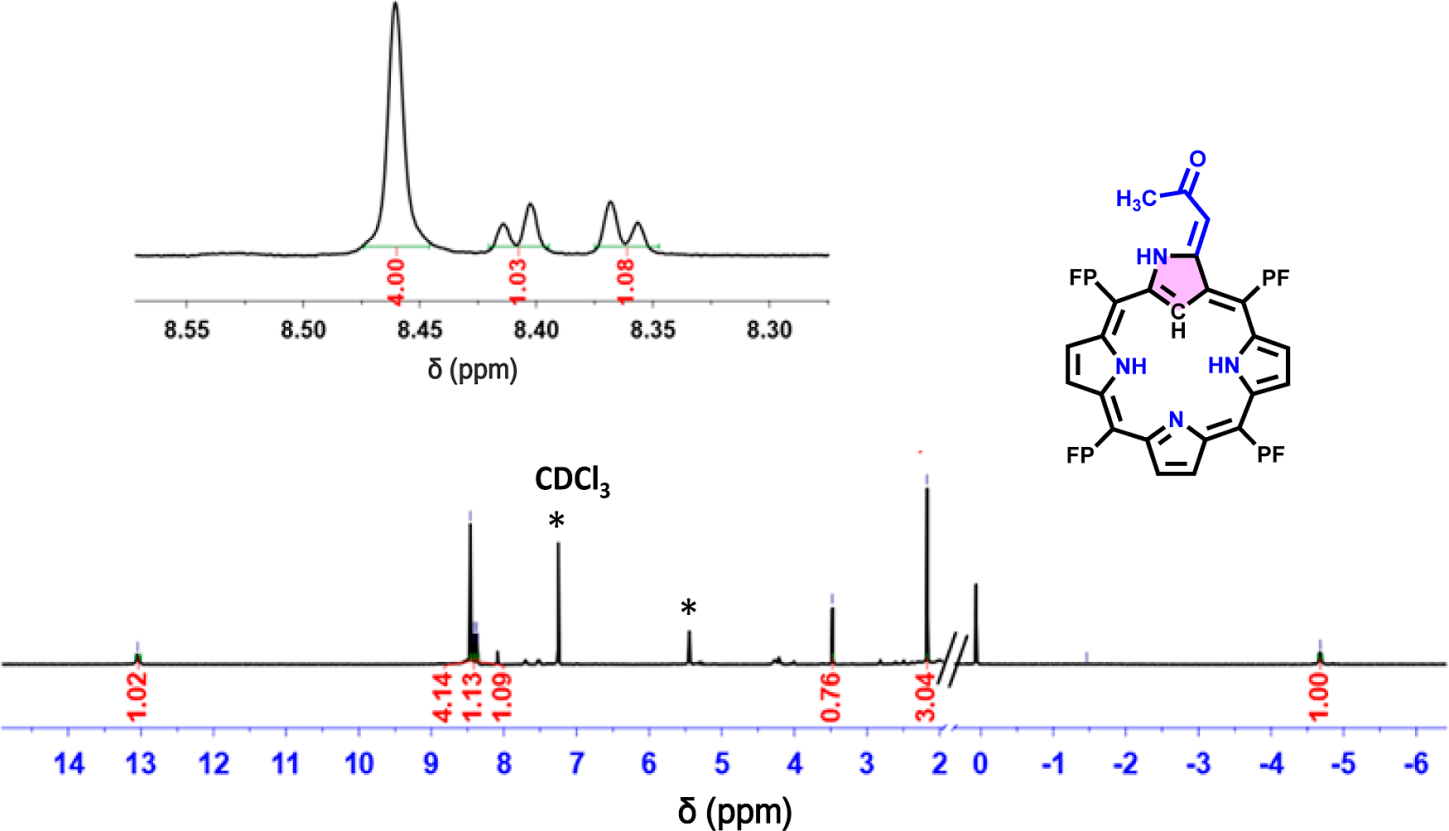
^1^H NMR (400 MHz) spectra of compound **3a** recorded in CDCl_3_ (* stands for solvent impurity).

**Figure 3:**
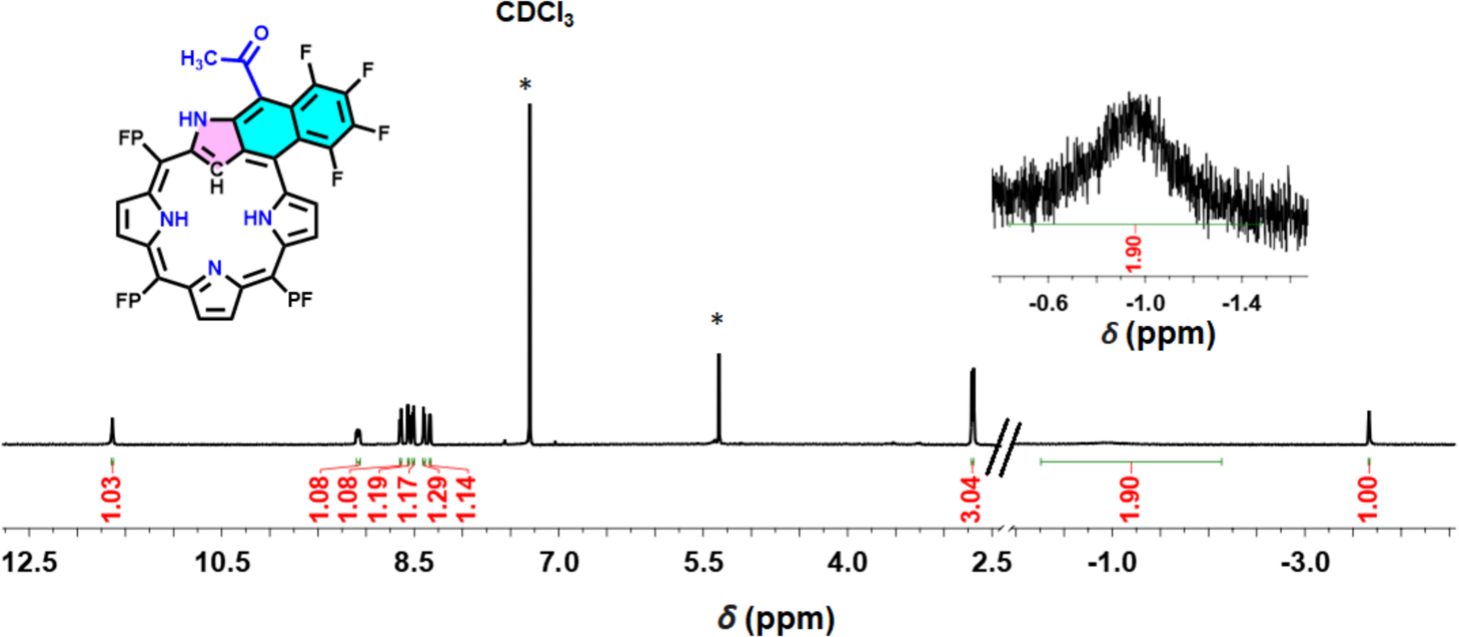
^1^H NMR (400 MHz) spectra of compound **5** recorded in CDCl_3_ (* stands for solvent impurity).

In Figure 2, six β-pyrrolic protons of compound **3a** appear in the range of 8.36–8.46 ppm in the ^1^H NMR spectrum. The sharp peak at −4.68 ppm corresponds to the inner C–H proton of the inverted pyrrole ring, while the inner pyrrolic N–Hs are too broad to be distinctly identified at room temperature. The presence of one methyl group on the 3-ylidene-2-propanone unit, resulting from the degradation of the acac group, is confirmed by the resonances of the methyl protons at 2.17 ppm and a vinyl proton at 3.48 ppm. The outer N–H on the inverted pyrrole ring is downfield-shifted and identified at 13.04 ppm. The exocyclic fused [6,6,5]-TF-PFNCP (**5**) exhibits a unique ^1^H NMR pattern, distinct from other PFNCP derivatives reported here, as shown in Figure 3. The two inner pyrrolic N–H and C–H protons are located at −1.02 and −3.71 ppm, respectively, with a ratio of 2:1. The outer NH of the inverted pyrrole ring, now part of the tricyclic structure, is found at 11.59 ppm. The transformation of compound **3a** into the tricyclic compound **5** is evidenced by the disappearance of the vinylic =CH proton signal, while the –CH_3_ resonance shifts slightly downfield to 2.67 ppm due to its new environment on the exterior of the conjugation ring system. The β-pyrrolic protons of the NCP core show chemical shifts between 8.29 and 9.06 ppm. The observed downfield shifts for the inner pyrrolic N–H and inner C–H protons in the NMR spectrum of **5**, relative to the corresponding resonances in compound **2a**, suggest a minor reduction in the aromatic character of the NCP core within [6,6,5]-TF-PFNCP (**5**). An increased number of resonance signals in both the ^1^H and ^19^F NMR (Figures S25 and S27) indicates a reduced symmetry in the fused macrocycle **5**.

Additionally, the ^1^H NMR spectra of the PFNCP 3-ketoester PFNCP-eacac (**2b**) and its acyl cleavage degradation product PFNCP-eac (**3b**) are presented in the Supporting Information (Figures S9 and S16). The ESI or MALDI-TOF high-resolution mass spectra of the NCP derivatives, recorded in positive ion mode, are shown in Figures S3, S8, S11, S15, S18, and S24. The measured *m*/*z* values match precisely with the calculated [M + H]^+^ mass values of all of the synthesized products.

### Single-Crystal X-ray Diffraction Structures

Single-crystal structures of compounds **2a**, **4**, and **5** were successfully resolved with high quality, providing not only confirmation on the identities of the substituents but also additional evidence supporting the tautomeric forms in the conjugation system. Crystals suitable for single-crystal X-ray diffraction analysis of **2a**, **4**, and **5** were grown through the slow diffusion of *n*-hexane into their respective solutions in CH_2_Cl_2_ or CHCl_3_ over a period of 1 week. Compound **2a**, with a solvated CH_2_Cl_2_ molecule, crystallized in the monoclinic *C*2/*c* space group. Meanwhile, compound **4** crystallized in the monoclinic *P*2_1_/*c* space group with a solvated CHCl_3_ molecule. In the crystal structure of compound **5**, which crystallized in the triclinic *P̅*1 space group due to significant solvent disorder, standard SQUEEZE procedures were employed to omit the electron density associated with the disordered solvent molecules. The charge density analysis revealed that the total electron count of the omitted electron density matches with two solvated CH_2_Cl_2_. All relevant crystallographic parameters are detailed in the Supporting Information (Tables S2–S13).

**Figure 4:**
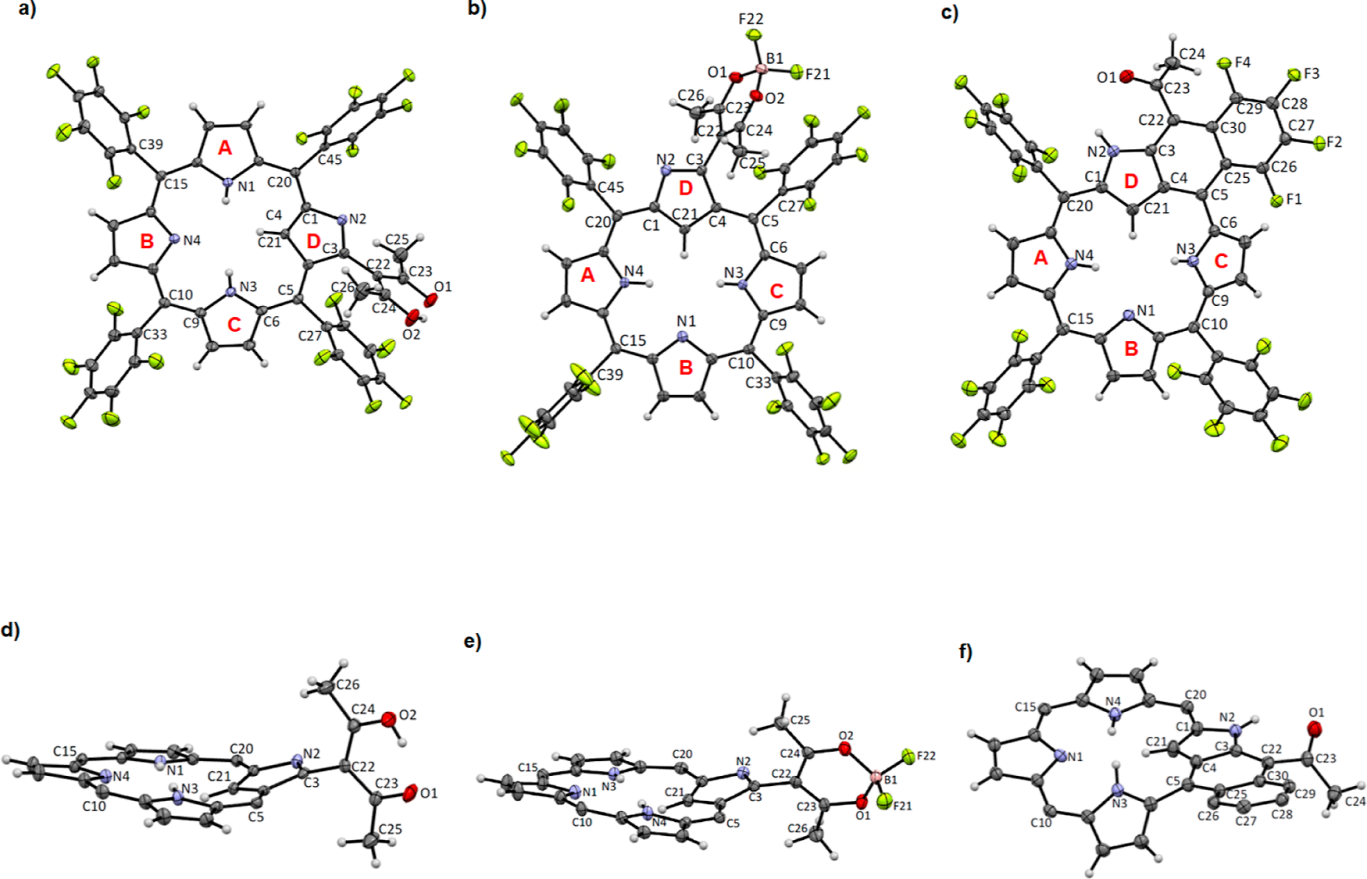
X-ray crystal structures (top view: a–c; side view: d–f) with atoms presented in 50% ellipsoids for compounds **2a**, **4**, and **5**, respectively. The *meso* groups were omitted for clarity in the side-view structures.

In the crystal structure of compound **2a**, depicted in Figure 4a, the acetylacetonate (acac) group at the 3-C position is distinctly visible. The enol tautomeric form is confirmed by a notably shorter carbonyl bond distance of 1.276(4) Å for C(23)–O(1) and a longer alkoxide bond distance of 1.302(4) Å for C(24)–O(2). The enol conjugation form is further evident from the significant difference in C–C bond lengths in the acac moiety, with C(22)–C(24) measuring 1.392(4) Å and C(22)–C(23) extending to 1.424(4) Å. The atoms in the acac unit are nearly coplanar, with an average deviation of 0.04 Å from the mean plane of the seven atoms. The 1.480(3) Å bond length of C(3)–C(22) connecting PFNCP and the acac unit affirms a single bond linkage, characteristic of the acac enol form. Importantly, the C(3) and N(2) atoms on the inverted pyrrole ring show no disorder, allowing for precise determination of all bond distances and angles in the PFNCP core without any orientation disorder. The high quality of the data also enables the identification of all three inner core protons from the difference charge density map, confirming the 3H tautomeric form for the PFNCP macrocycle.

In the PFNCP macrocycle, as shown in Figure 4d, the porphyrin core of **2a** exhibits a buckled conformation due to steric hindrance from the three inner protons, with the inverted pyrrole ring tilted away from the mean plane. The twisting angle of this inverted pyrrole ring relative to the mean plane, defined by the 17 atoms of the tripyrrin unit, is 22.96°. The average deviation of the atoms in the inverted pyrrole ring from the mean plane is 0.475 Å. All pentafluorophenyl rings, along with the acac unit, are oriented almost perpendicular to the tripyrrin plane. The average distance between the acac mean plane and the adjacent pentafluorophenyl ring is 3.06 Å. Additionally, the crystal structure is characterized by extensive intermolecular interactions, including fluorine–fluorine interactions, hydrogen bonding between outer pyrrolic protons and fluoride atoms, and interactions between fluoride atoms and inner protons, leading to a densely packed molecular structure.

The ORTEP plots of compound **4**, depicted with 50% thermal ellipsoids, are illustrated in Figure 4b,e. These plots show that the overall conformation and bond distances of the PFNCP core in compound **4** are similar to those in compound **2a**. This similarity indicates that both compounds share the same tautomeric form of the N-confused porphyrin core, with minimal perturbation of electronic structures between the orthogonal PFNCP and acac units. However, the chelation of BF_2_ in compound **4** induces a transformation of the acac unit from the enol to the diketonate form. This change results in near identical C–O bond distances, measured at 1.304(3) Å for C(23)–O(1) and 1.298(3) Å for C(24)–O(2). Additionally, the delocalization of π-electrons in the diketonate form leads to near-identical C–C bond distances of 1.391(4) Å for C(22)–C(24) and 1.396(4) Å for C(22)–C(23).

The asymmetric unit of compound **5** is characterized by a distinctive porphyrinic structure featuring three pyrrole rings (A, B, and C), three *meso*-carbons, and a tricyclic fused ring, as illustrated in Figure 4c,f. The tricyclic ring is formed by fusing the inverted pyrrole ring and a *meso*-phenyl moiety through the elimination of HF from the 3-ylidene-2-propanone substituent and an *ortho*-fluoride of a *meso*-pentafluorophenyl group on **3a**. This tricyclic ring exhibits a slight buckle, with an average deviation of 0.1739 Å from its mean plane, likely due to steric hindrance between the acetyl substituent and F(4) on the phenyl ring. The bond lengths within the tricyclic ring suggest a delocalized π-system as they fall between those typical of single and double bonds. The acetyl substituent on the tricyclic ring shows a bond length of 1.508(3) Å for C(23)–C(24) and a shorter distance of 1.223(3) Å for C(23)–O(1).

The porphyrin core in compound **5** is notably twisted, a consequence of steric constraints from F(1) on the tricyclic fused ring and the adjacent pyrrolic ring C. The twisting angle, defined by the average plane of the tricyclic structure and the mean plane of 11 atoms on NCP from ring A, ring B, and C(15), is measured at 13.58°. Ring C, in particular, shows significant distortion from the dipyrromethene mean plane with a tilting angle of 27.43°. The extended bond lengths of 1.439(3) Å (C(5)–C(6)) and 1.424(3) Å (C(1)–C(20)), connecting the tricyclic ring to the porphyrinic conjugation system, along with the large twisting angle between ring C and the tricyclic ring, indicate limited π-delocalizing between the porphyrinic conjugation and tricyclic ring. This discrete conjugation leads to a decrease in aromaticity, as reflected in the spectroscopic data of compound **5**.

Besides the three high-quality single-crystal structures discussed above, there have been efforts to obtain high-quality crystals of compound **3a**, albeit without success. However, a crystal structure data set for **3a** was acquired by slow diffusion of *n*-hexane into CHCl_3_ over a period of 4 days and refined to reach a *R*_1_ index of 0.1809 (for *I* > 2σ(*I*)), as detailed in Table S11 of the Supporting Information. Although this data set exhibits larger deviations in bond lengths and angles, preventing the determination of precise geometry for **3a**, the 3-ylidene-2-propanone substituent is clearly identifiable, as shown in Figure S31. Notably, the shorter bond distances of 1.315(15) Å for C(3)–C(22) and 1.301(17) Å for C(23)–O(1) align with the proposed structure for **3a**.

### Photophysical Properties

**Figure 5:**
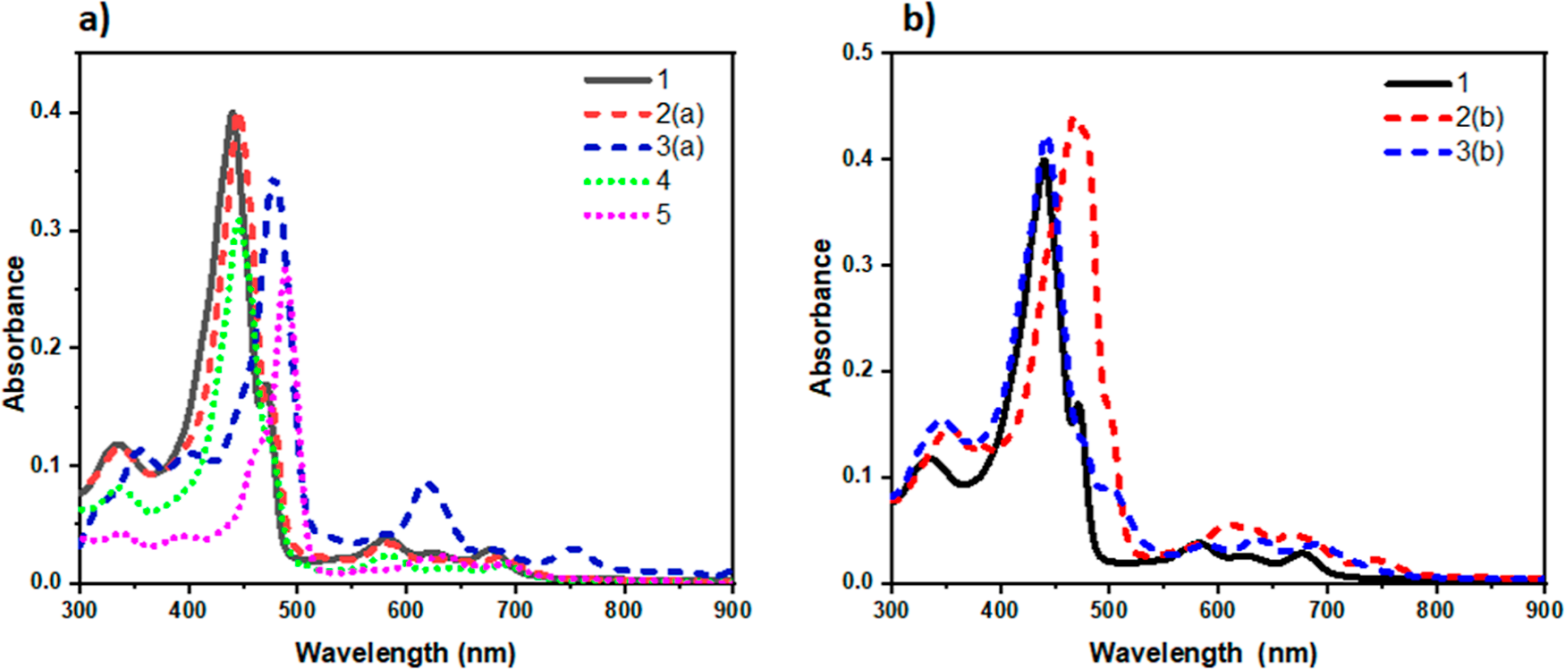
Comparison of absorption spectra of the PFNCP derivatives (2 × 10^–5^ M) recorded in CH_3_CN at room temperature: (a) **1**, **2a**, **3a**, **4**, and **5**; (b) **2b** and **3b**.

**Figure 6:**
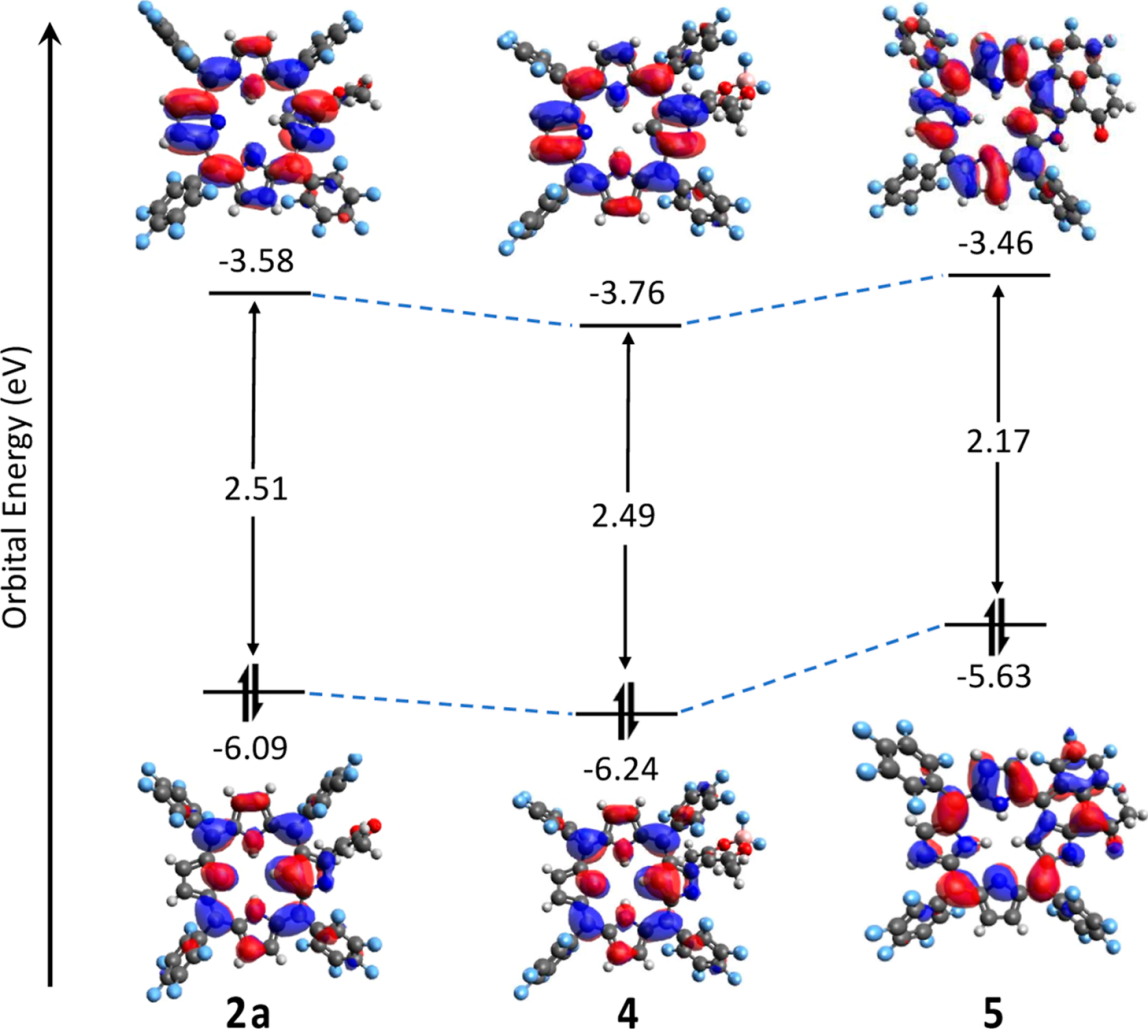
Energy levels and electron density distribution of the frontier orbitals for the studied compounds (**2a**, **4**, and **5**).

The absorption spectra of compounds **2–5** were analyzed in a CH_3_CN solution, and the results are displayed in Figure 5 and Table S1 (with (λ_abs_, nm (log ε_max_)). These PFNCP derivatives exhibit a prominent *Soret* band in the 400–550 nm range and multiple weaker *Q* bands in the 580–680 nm low-energy region. The spectrum of compound **2a** closely resembles that of PFNCP **1**, while compound **3a** shows a slight red shift in the absorption spectra. The chelation of BF_2_ to acac induces a slight red shift in the absorption spectrum. Notably, compound **5**, the fused-ring product, shows a 48 nm red shift in the Soret band compared to that of **1**. To elucidate further, density functional theory (DFT) calculations were conducted, revealing the energy levels and electron density distributions in the highest occupied molecular orbitals (HOMOs) and lowest unoccupied molecular orbitals (LUMOs), as illustrated in Figure 6. Consistent with the absorption spectra, both the HOMO and LUMO exhibit downward shifts following BF_2_ chelation in compound **4**. This alternation slightly reduces the energy gap between the HOMO and LUMO, causing a minor red shift in the absorption spectrum of compound **4**. Ring fusion in compound **5** leads to upward shifts in both the HOMO and LUMO compared to **2a** and **4**. A significant elevation of the HOMO in **4** results in a substantial decrease in the HOMO–LUMO gap, contributing to the red shift observed in its absorption spectrum.

Interestingly, the electron density in both HOMO and LUMO of **2a** and **4** shows minimal distribution to the acac and acac-BF_2_ moieties, aligning with the orthogonal geometry between the PFNCP core and its 3-C substituents. In contrast, compound **5** exhibits an even electron density distribution across the porphyrin core and tricyclic ring in the HOMO, with a significant density also noted in the tricyclic fused ring of the LUMO.

## Conclusions

We have successfully synthesized the first example of N-confused porphyrin substituted with acetylacetonate and ylidene-2-propanone at the 3-C position without a catalyst using a one-pot reaction approach. These derivatives exhibit remarkable stability. The acetylacetonate-substituted NCP efficiently forms the corresponding BF_2_ complex when treated with K_2_CO_3_ as a mild base and BF_3_·OEt_2_ in toluene. Furthermore, direct synthesis of ylidene-2-propanone-substituted NCP from acetylacetonate-substituted NCP is achieved through acyl cleavage degradation catalyzed by an acid. The ylidene-2-propanone derivative undergoes HF elimination to yield a tricyclic ring-fused macrocycle. Comprehensive structural elucidation using single-crystal X-ray analysis and ^1^H NMR spectroscopy provided detailed insights into their molecular configurations. Absorption spectra revealed that all macrocycles show a prominent Soret band in the 400–550 nm range and several weaker *Q* bands between 580 and 680 nm, with the tricyclic fused macrocycle showing a notable red shift in the Soret band. DFT calculations corroborate these experimental findings, supporting the structural and electronic properties of the synthesized macrocycles.

## Experimental Section

### General

All of the reagents were purchased from Sigma-Aldrich and used as received. NMR spectra were recorded with either a Bruker Advance IIIHD 400 MHz or a Bruker DRX 500 NMR spectrometer. High-resolution mass spectra, reported as millimeters per second, were obtained using a JMS-700 double-focusing mass spectrometer (JEOL, Tokyo, Japan) for HRMS (FAB) or a JMST100LP AccuTOF LC-plus 4G mass spectrometer for HRMS (ESI). UV–visible spectra were collected by using an Agilent 8453 UV–visible spectrophotometer. Single-crystal X-ray diffraction measurement data were obtained using a Bruker-AXS APEX CCD diffractometer with graphite-monochromated Mo-Kα radiation (λ = 0.71073 Å). The SHELXL-97 program with full-matrix least-squares on *F*^2^ values and direct methods were used for the refinement of the crystallographic data. All non-hydrogen atoms were refined anisotropically, while the hydrogen atoms were located in ideal, calculated positions, with isotropic thermal parameters riding on their respective carbon atoms. Cambridge Crystallographic Data Centre (CCDC) deposition numbers 2322601 (for **2a**), 2322592 (for **4**), and 2322785 (for **5**) contain the supplementary crystallographic data for this paper. Density functional theory (DFT) calculations were executed by using the ORCA quantum chemical software (Version 5.0). The atomic structures of compounds **2a**, **4**, and **5**, derived from X-ray single-crystal analyses, were directly used for single-point energy evaluations. These calculations were conducted without modifying or optimizing the structures utilizing the B3LYP hybrid-GGA (generalized gradient approximation) density functional^50,51^ alongside the Ahlrichs def2-TZVP triple-ζ basis set^52^ and the corresponding auxiliary basis set (def2/J).^53^

### Synthesis of 5,10,15,20-Tetrakis(pentafluorophenyl)-2-aza-21-carbaporphyrin (Pentafluorophenyl-*N*-confused-porphyrin, PFNCP, **1**)

In a 2 L round-bottom flask, CH_2_Cl_2_ (1.5 L), pyrrole (0.693 mL, 10.0 mmol), and pentafluorobenzaldehyde (1.23 mL, 10 mmol) were added. The reaction was initiated by the addition of trifluoromethanesulfonic acid (0.09 mL, 1.0 mmol) under an inert atmosphere. The reaction mixture was stirred continuously for 24 h at room temperature under a nitrogen atmosphere. The reaction was quenched by the addition of triethylamine (0.5 mL, 4 mmol), followed by oxidation with DDQ (2.50 g, 11.02 mmol), and stirred for 30 min. The solvent was removed by rotatory evaporation. The crude sample was passed through a column of 4 cm × 16 cm filled with silica slurry in *n*-hexane/CH_2_Cl_2_ (3:1). The polarity of the eluant was increased from 3:1, 2:1, 1:1 to 1:2 of *n*-hexane/CH_2_Cl_2_. Initially, *meso*-tetrakis(pentafluorophenyl)porphyrin began to slowly elute at *n*-hexene/CH_2_Cl_2_ (3:1), and after increasing the polarity, 1 was eluted with around 1:1 to 1:2 of *n*-hexane/CH_2_Cl_2_. The combined fractions were evaporated to dryness and placed under vacuum to obtain 198 mg of 1 as a purple crystalline solid (8.5%).

#### PFNCP (**1**)

^1^H NMR (400 MHz, CDCl_3_) δ: 8.98 (d, *J* = 4.7 Hz, 1H), 8.92 (d, *J* = 8.5 Hz, 2H), 8.70 (t, *J* = 4.2 Hz, 2H), 8.67 (d, *J* = 4.7 Hz, 1H), 8.64 (d, *J* = 4.7 Hz, 1H), −2.52 (d, *J* = 27.2 Hz, 2H), −5.23 (s, 1H). HRMS (ESI-TOF) *m*/z: [M + H]^+^ calcd for C_44_H_11_F_20_N_4_, 975.0658; found 975.0649. λ_max_ (log ε) 335 (4.76), 440 (5.29), 471 (4.92), 581 (4.27), 624 (4.2), 679 (4.14).

### Synthesis of 4-Hydroxy-3-((15*s*,20*s*)-5,10,15,20-tetrakis(perfluorophenyl)-2-azaporphyrin-3-yl)pent-3-en-2-one (PFNCP-acac, **2a**)

In a 50 mL two-neck round-bottom flask, PFNCP (**1**) (50 mg, 0.05 mmol) was dissolved in CH_2_Cl_2_ under a nitrogen atmosphere. After that, 2,4-pentanedione (10 mL, 9.8 mmol) was added to the reaction mixture of MeOH and CH_2_Cl_2_ (2:1) and refluxed at 60–70 °C (oil bath) for 24 h under N_2_. The solution turned from green to brown. The reaction was stopped after 24 h, and the solvent was removed by rotatory evaporation. The crude product was purified by silica gel column chromatography by using a CH_2_Cl_2_/hexene (50:50) mixture. We obtained the green crystalline solid compound with a 30% yield (15 mg) of PFNCP-ac (**3a**) after recrystallization (CH_2_Cl_2_ and hexane 1:2). PFNCP-acac (**2a**) was obtained at higher polarity using CH_2_Cl_2_/MeOH (98:2) as a brown crystalline solid with a 50% yield (25 mg) after drying.

#### PFNCP-acac (**2a**)

^1^H NMR (400 MHz, CDCl_3_) δ: 16.36 (s, 1H), 8.93 (d, *J* = 4.8 Hz, 1H), 8.77 (d, *J* = 5.0 Hz, 1H), 8.62–8.53 (m, 3H), 8.52 (d, *J* = 4.8 Hz, 1H), 1.88 (s, 6H), −2.06 (d, *J* = 140.8 Hz, 2H), −4.98 (s, 1H). ^13^C{^1^H} NMR (101 MHz, CDCl_3_) δ: 192.2, 161.9, 158.9, 157.1, 149.3, 148.1, 147.2, 145.7, 144.8, 144.1, 141.4, 140.0, 138.6, 138.6, 137.0, 135.9, 135.1, 134.4, 129.8, 128.4, 127.0, 125.7, 115.5, 114.3, 112.5, 110.6, 106.7, 104.1, 101.3, 30.1, 24.5. ^19^F NMR (376 MHz, CDCl_3_) δ: −136.74 (s, 8F), −138.33 (d, *J* = 19.4 Hz, 2F), −150.72 (s, 2F), −160.70 (d, *J* = 88.7 Hz, 6F), −161.72 (S, 2F). HRMS (ESI-TOF) *m*/z: [M + H]^+^ calcd for C_49_H_17_N_4_O_2_F_20_, 1073.1026; found 1073.1035. UV/vis (CH_3_CN, λ_max_ (log ε)): 337 (4.75), 445 (5.3), 475 (4.87), 583 (4.23), 637 (4.00), 687 (4.06).

#### PFNCP-ac (**3a**)

^1^H NMR (400 MHz, CDCl_3_) δ: 13.04 (s, 1H), 8.46 (s, 4H), 8.42 (d, *J* = 4.7 Hz, 1H), 8.37 (d, *J* = 4.7 Hz, 1H),3.48 (s, 1H), 2.17 (s, 3H), −1.46 (s, 2H), −4.68 (s, 1H). ^13^C{^1^H} NMR (101 MHz, CDCl_3_) δ: 198.1, 152.6, 147.8, 147.3, 145.3, 144.7, 142.6, 141.2, 139.7, 138.4, 136.4, 133.8, 133.1, 131.1, 129.1, 127.0, 126.3, 124.9, 115.4, 111.7, 105.1, 102.7, 99.9, 96.6, 94.1, 65.8, 30.3, 29.9. ^19^F NMR (376 MHz, CDCl_3_) δ: −136.92, −137.22 (m, 6F), −137.78 (dd, *J* = 24.5, 8.9 Hz, 2F), −149.17 (t, *J* = 19.9 Hz, 1F), −150.36, −150.89 (m, 1F), −151.27 – −151.89 (m, 2F), −159.10, −159.61 (m, 4F), −160.75, −161.63 (m, 4F). HRMS (ESI) *m*/z: [M + H]^+^ calcd for C_47_H_15_N_4_OF_20_, 1031.0921; found 1031.0929. UV/vis (CH_3_CN, λ_max_ (log ε)): 351 (4.83), 385 (4.80), 461 (5.19), 486 (5.21), 633 (4.44), 669 (4.42).

### Synthesis of 1-((15*s*,20*s*)-5,10,15,20-Tetrakis(perfluorophenyl)-2*H*,3*H*-2-azaporphyrin-3-ylidene)propan-2-one (PFNCP-ac, **3a**) from **2a** (PFNCP-acac)

Compound **2a** (PFNCP-acac, 50 mg, 0.04 mmol) was taken in a 50 mL two-necked round-bottom flask. It was dissolved in 10 mL of toluene and kept in a nitrogen atmosphere for 10 min. Then, 200 μL of acetic acid was added to the solution, and the reaction was heated for 4 h at around 55 °C (oil bath). The reaction mixture was subjected to silica gel column chromatography using CH_2_Cl_2_/hexene (50:50) to obtain PFNCP-ac (**3a**) as a green crystalline solid with a 60% yield (30 mg) after drying.

### Synthesis of Ethyl-3-hydroxy-2-(5,10,15,20-tetrakis(perfluorophenyl)-2-azaporphyrin-3-yl)but-2-enoate (PFNCP-eacac, **2b**)

PFNCP (**1**) (50 mg, 0.05 mmol) was taken in a two-neck round-bottom flask. Ethyl acetoacetate (1 mL, 7.8 mmol) was dissolved in CH_2_Cl_2_/CH_3_OH (2:1) and added to the reaction. The reaction mixture was refluxed at 60–70 °C (oil bath) for 24 h under N_2_. After reaction, the mixture was allowed to cool and washed with water 2–3 times to remove the unreacted ethyl acetoacetonate from the reaction mixture. The crude compound was subjected to silica gel column chromatography. PFNCP-eac (**3b**) was obtained with CH_2_Cl_2_/hexene (50:50) as a green color solid with a 40% yield (20 mg). More polar brown color spot PFNCP-eacac (**2b**) was obtained with CH_2_Cl_2_/hexene (60:40) as a brown crystalline solid with a 55% yield (27.5 mg).

#### PFNCP-eacac (**2b**)

^1^H NMR (400 MHz, CDCl_3_) δ: 13.02 (s, 1H), 8.90 (d, *J* = 3.0 Hz, 1H), 8.76 (d, *J* = 3.2 Hz, 1H), 8.58 (d, *J* = 4.6 Hz, 3H), 8.52 (d, *J* = 4.5 Hz, 1H), 4.24–4.01 (m, 2H), 2.26 (s, 3H), 0.61 (t, 3H), −2.05 (d, *J* = 91.3 Hz, 2H), −4.98 (s, 1H). ^13^C{^1^H} NMR (101 MHz, CDCl_3_) δ: 171.8, 161.3, 158.5, 156.8, 148.2, 145.3, 144.1, 141.3, 139.5, 138.5, 137.2, 135.7, 134.9, 129.6, 128.2, 127.9, 126.7, 125.5, 111.9, 103.9, 100.9, 61.6, 30.2, 20.4. HRMS (ESI-TOF) *m*/z: [M + H]^+^ calcd for C_50_H_19_N_4_O_3_F_20_, 1103.1132; found 1103.1140. UV/vis (CH_3_CN, λ_max_ (log ε)): 345 (4.89), 442 (5.32), 501 (4.64), 587 (4.23), 633 (4.30), 698 (4.25).

#### PFNCP-eac (**3b**)

^1^H NMR (400 MHz, CDCl_3_) δ: 11.40 (s, 1H), 8.43 (d, *J* = 4.9 Hz, 2H), 8.39–8.37 (m, 3H), 8.34 (d, *J* = 4.7 Hz, 1H), 5.04 (s, 1H), 4.24–4.19 (q, *J* = 7.1 Hz, 2H), 0.88 (dd, *J* = 12.4, 5.4 Hz, 3H), −4.48 (d, *J* = 1.5 Hz, 1H). ^13^C{^1^H} NMR (101 MHz, CDCl_3_) δ: 170.5, 155.7, 154.2, 153.4, 148.0, 147.5, 145.5, 145.1, 143.4, 140.1, 138.8, 138.1, 135.9, 134.5, 133.7, 132.9, 127.2, 126.8, 126.4, 124.5, 105.4, 102.9, 99.0, 92.6, 89.6, 60.7, 30.1. HRMS (ESI) *m*/z: [M + H]^+^ calcd for C_48_H_17_N_4_O_2_F_20_, 1061.1027; found 1061.1032. UV/vis (CH_3_CN, λ_max_ (log ε)): 353 (4.88), 467 (5.34), 478 (5.32), 500 (4.91), 610 (4.43), 668 (4.36), 745 (4.04).

### Synthesis of Ethyl (*E*)-2-(5,10,15,20-tetrakis(perfluorophenyl)-2*H*,3*H*-2-azaporphyrin-3-ylidene)acetate (PFNCP-eac, **3b**) from **2b** (PFNCP-eacac)

Compound **2b** (PFNCP-eacac, 50 mg, 0.045 mmol) was taken in a 50 mL two-necked round-bottom flask. It was dissolved in 10 mL of toluene and kept in a nitrogen atmosphere for 10 min. Then, 200 μL of acetic acid was added to the solution, and the reaction was heated for 4 h at around 55 °C (oil bath). Then, the reaction mixture was diluted with CH_2_Cl_2_ and washed with a saturated solution of Na_2_CO_3_. The crude product was purified by silica gel column chromatography using CH_2_Cl_2_/hexene (50:50) to obtain PFNCP-eac (**3b**) as a green crystalline solid with a 63% yield (31.5 mg) after drying.

### Synthesis of (15*s*,20*s*)-3-(2,2-Difluoro-4,6-dimethyl-2*H*-1l3,3,2l4-dioxaborinin-5-yl)-5,10,15,20-tetrakis(perfluorophenyl)-2-azaporphyrin (PFNCP-acacBF_2_, **4**)

In a 50 mL double-neck round-bottom flask, 50 mg (0.04 mmol) of PFNCP-acac (**2a**) was dissolved in 25 mL of toluene. K_2_CO_3_ (55 mg, 0.4 mmol) was added to the solution, followed by slow addition of BF_3_·OEt_2_ (113.5 μL, 0.92 mmol). The solution changed from brown to green. Then, the reaction mixture was stirred at 55 °C (oil bath) for 6 h. The solvent was evaporated by rotatory evaporation, and the residue was neutralized by NaHCO_3_ and extracted twice with 50 mL of CH_2_Cl_2_. The combined organic layers were dried over anhydrous Na_2_SO_4_, and the solvent was removed on a rotary evaporator under vacuum. The crude compound was purified by silica gel chromatography using CH_2_Cl_2_/*n*-hexane (80:20) to afford PFNCP-acacBF_2_ (**4**) as an orange crystalline solid compound with a 75% yield (33.5 mg).

#### PFNCP-acacBF_2_ (**4**)

^1^H NMR (400 MHz, CDCl_3_) δ: 8.93 (d, *J* = 4.9 Hz, 1H), 8.78 (d, *J* = 5.0 Hz, 1H), 8.64–8.60 (m, 2H), 8.59 (d, *J* = 4.8 Hz, 1H), 8.53 (d, *J* = 4.8 Hz, 1H), 1.88 (s, 6H), −2.06 (d, *J* = 81.2 Hz, 2H), −4.98 (s, 1H). ^13^C{^1^H} NMR (101 MHz, CDCl_3_) δ: 192.4, 159.1, 157.2, 148.2, 145.7, 141.9, 140.4, 138.6, 137.3, 136.1, 135.4, 133.1, 130.2, 129.1, 127.3, 126.1, 113.3, 112.1, 107.0, 104.8, 104.1, 101.7, 100.9, 53.8, 23.9. ^19^F NMR (376 MHz, CDCl_3_) δ: −136.30, −137.11 (m, 6F), −138.16, −138.62 (m, 2F), −138.89 (d, *J* = 16.4 Hz, 2F), −146.45 (t, *J* = 19.0 Hz, 1F), −150.36 (t, *J* = 20.9 Hz, 1F), −150.55 (td, *J* = 20.7, 11.3 Hz, 2F), −159.72 (t, *J* = 18.5 Hz, 2F), −160.73 (dt, *J* = 22.9, 8.0 Hz, 4F), −161.69 (td, *J* = 21.7, 6.4 Hz, 2F). ^11^B NMR (128 MHz, CDCl_3_) δ: 0.25 (dd, *J* = 10.5, 6.0 Hz, 1H). MS (MALDI-TOF) *m*/z: [M + H]^+^ calcd for C_49_H_16_N_4_O_2_BF_22_, 1121.10; found 1121.10. UV/vis (CH_3_CN, λ_max_(log ε)): 337 (4.60), 445 (5.18), 475 (4.77), 583 (4.06), 641 (3.81), 689 (4.47).

### Synthesis of [6,6,5]-Tricyclic Fused PFNCP ([6,6,5]-TF-PFNCP, **5**)

In a 50 mL double-neck round-bottom flask, 50 mg (0.04 mmol) of PFNCP-ac (**3a**) was dissolved in 20 mL of DMF, and the mixture was refluxed (oil bath) for 2 h under a nitrogen atmosphere. After that, the reaction was stopped, and DMF was removed using the distillation method. The crude compound was purified by using silica gel chromatography using CH_2_Cl_2_/hexene (50:50) to obtain [6,6,5]-TF-PFNCP (**5**) as a green crystalline solid with an 18% yield (9 mg). Using a similar reaction procedure, the reaction of PFNCP-acac (**2a**) in DMF under reflux for 4 h isolated 5 with a 12% yield (6 mg).

#### [6,6,5]-TF-PFNCP (**5**)

^1^H NMR (400 MHz, CDCl_3_) δ: 11.59 (s, 1H), 9.06 (dd, *J* = 10.2, 4.8 Hz, 1H), 8.63 (d, *J* = 5.1 Hz, 1H), 8.53 (d, *J* = 5.0 Hz, 1H), 8.48 (d, *J* = 4.6 Hz, 1H), 8.36 (d, *J* = 4.7 Hz, 1H), 8.30 (d, *J* = 4.7 Hz, 1H), 2.67 (d, *J* = 8.7 Hz, 3H), −1.02 (s, 2H), −3.71 (s, 1H).^13^C{^1^H} NMR (101 MHz, CDCl_3_) δ: 201.5, 157.5, 152.3, 148.0, 145.6, 142.5, 140.0, 138.6, 136.5, 136.0, 135.0, 133.9, 132.4, 127.8, 127.7, 125.9, 123.4, 115.3, 111.9, 109.4, 108.7, 100.7, 97.3, 93.3, 31.4. ^19^F NMR (376 MHz, CDCl_3_) δ: −135.55 (d, *J* = 8.3 Hz, 2F), −137.10 (ddd, *J* = 69.3, 38.8, 11.8 Hz, 6F), −149.26 (t, *J* = 20.9 Hz, 1F), −151.65 (dd, *J* = 43.0, 21.1 Hz, 2F), −153.55, −154.03 (m, 1F), −159.04 (ddd, *J* = 51.0, 31.0, 17.7 Hz, 3F), −160.63, −162.29 (m, 4F). HRMS (ESI-TOF) *m*/z: [M + H]^+^ calcd for C_47_H_14_N_4_O_5_F_19_, 1011.0858; found 1011.0856. UV/vis (CH_3_CN, λ_max_ (log ε)): 337 (4.32), 395 (4.30), 488 (5.12), 561 (3.74), 612 (4.00), 635 (4.06), 666 (3.87), 691 (3.95).

## Data Availability

The data underlying this study are available in the published article and its Supporting Information.

## Supporting Information Available

Characterization data (HRMS, ^1^H NMR, ^13^C NMR, and absorption spectra) and crystallographic characterization (PDF)
