# Supplementary material for: Expanding the Chemistry of Pentafluorophenyl-N-Confused Porphyrin: Diketonate Substitution and Derivatizations at the External 3-C Position of the Inverted Pyrrole Ring
Source: ACS Org Inorg Au. 2024 Oct 1;4(6):681–91. doi: 10.1021/acsorginorgau.4c00065 (PMC11621957; doi:10.1021/acsorginorgau.4c00065)
Supplement: Supplementary file 1 — gg4c00065_si_001.pdf [file gg4c00065_si_001.pdf]

Supporting Information

Expanding the Chemistry of Pentafluorophenyl-N-Confused Porphyrin: Diketonate Substitution and Derivatizations at the External 3-C Position of Inverted Pyrrole Ring

Bhakyaraj Kasi[a,b,c], Belarani Ojha[c], Wen-Feng Liaw[b]* and Chen-Hsiung Hung[c]*

[a]Molecular Science and Technology Program, Taiwan International Graduate Program, Academia Sinica, Taipei 115201, Taiwan

[b]Department of Chemistry, National Tsing Hua University, Hsinchu 300044, Taiwan

[c]Institute of Chemistry, Academia Sinica, Nankang Taipei 115201, Taiwan

E-mail: chhung@gate.sinica.edu.tw

Sr. no	Contents	Page no.
1	**Figure. S1** to **S27.** Characterization data (NMR and HR mass spectra) of all new compounds	S2-S28
2	**Figure. S28**. Absorption spectra of all new compounds recorded in CH_3CN	S29
3	**Figure. S29-S32.** X-ray structure of compound **2a, 3a**, **4**, and **5**	S30-S33
5	**Tables S1.** Absorption spectra data of compound **2- 5**	S34
6	**Tables S2** to **S13.** X-ray data of compound **2a,3 a**, **4**, and **5**	S35-S54
7	**Table S14-S16.** Cartesian coordinates and computed total energies of molecules **2a**, **4** , and **5**	S55-S78

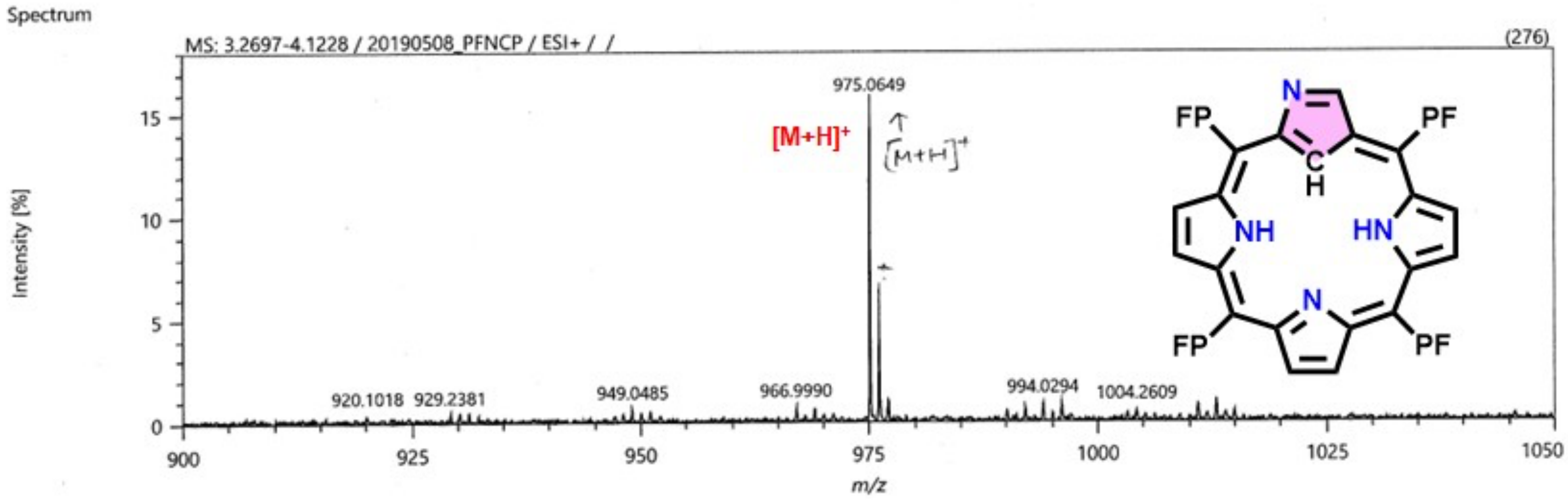

Elemental Composition

Parameters	
Tolerance:	±20.00 ppm
Electron:	Odd/Even
Charge:	+1
DBE:	-1.5 - 999.0

Elements Set 1:

Symbol	C	H	O	Re	Cl	F	N	S
Min	0	0	0	0	0	20	4	0
Max	400	1000	0	0	0	20	4	0

Results

Mass	Formula	Calculated Mass	Mass Difference [mDa]	Mass Difference [ppm]	DBE
975.06494	C44 H11 N4 F20	975.06589	-0.95	-0.97	31.5

Figure S1. HR mass spectrum of the compound **1**

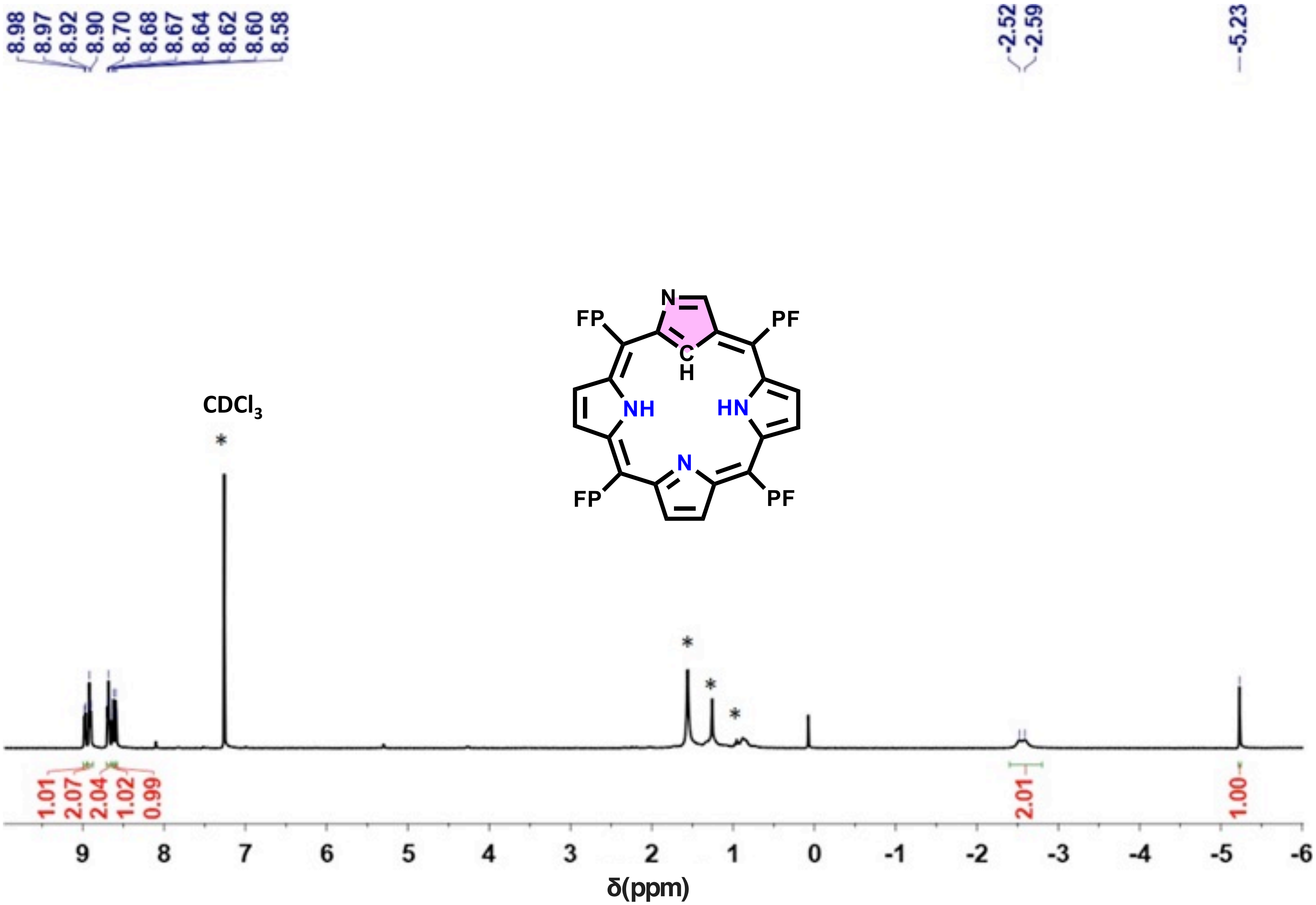

Figure S2. ^{1}H NMR (400 MHz) spectrum of the compound **1** recorded in $CDCl_3$. The Peaks with * are due to solvent impurities.

Spectrum

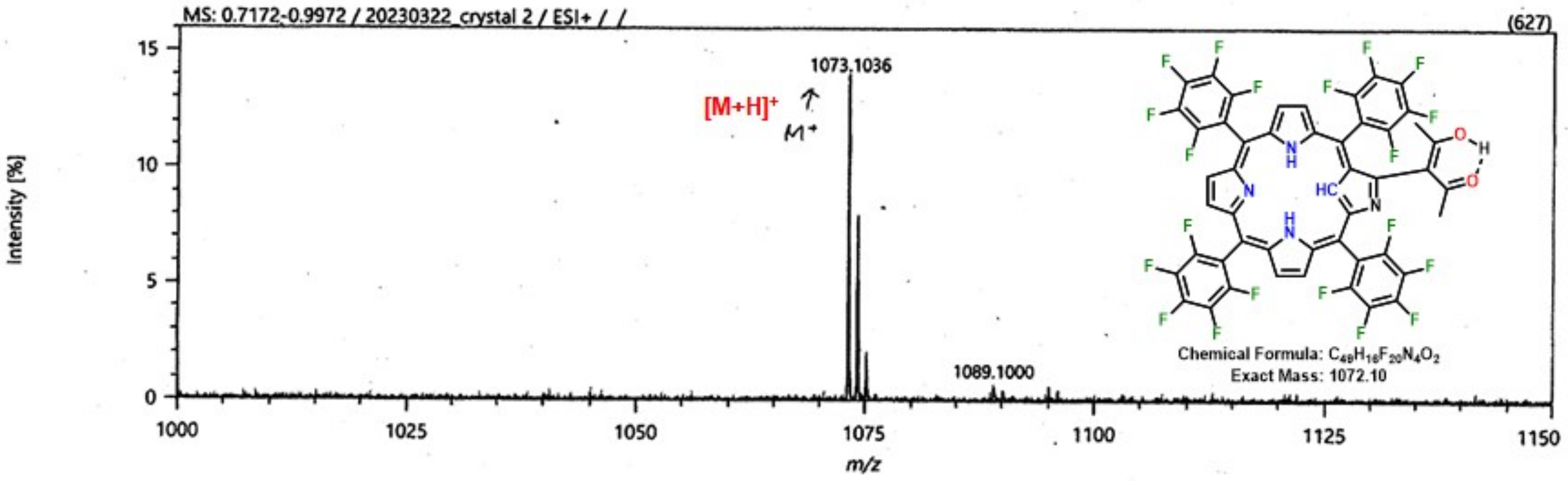

Elemental Composition

Parameters

Tolerance:	±10.00 ppm
Electron:	Odd/Even
Charge:	+1
DBE:	-99.0 - 999.0

Elements Set 1:

Symbol	C	H	O	N	F
Min	0	0	2	4	20
Max	100	400	2	4	20

Results

Mass	Formula	Calculated Mass	Mass Difference [mDa]	Mass Difference [ppm]	DBE
1073.10358	C49 H17 N4 O2 F20	1073.10267	0.92	0.85	33.5

Figure S3. HR mass spectrum of the compound **2a.**

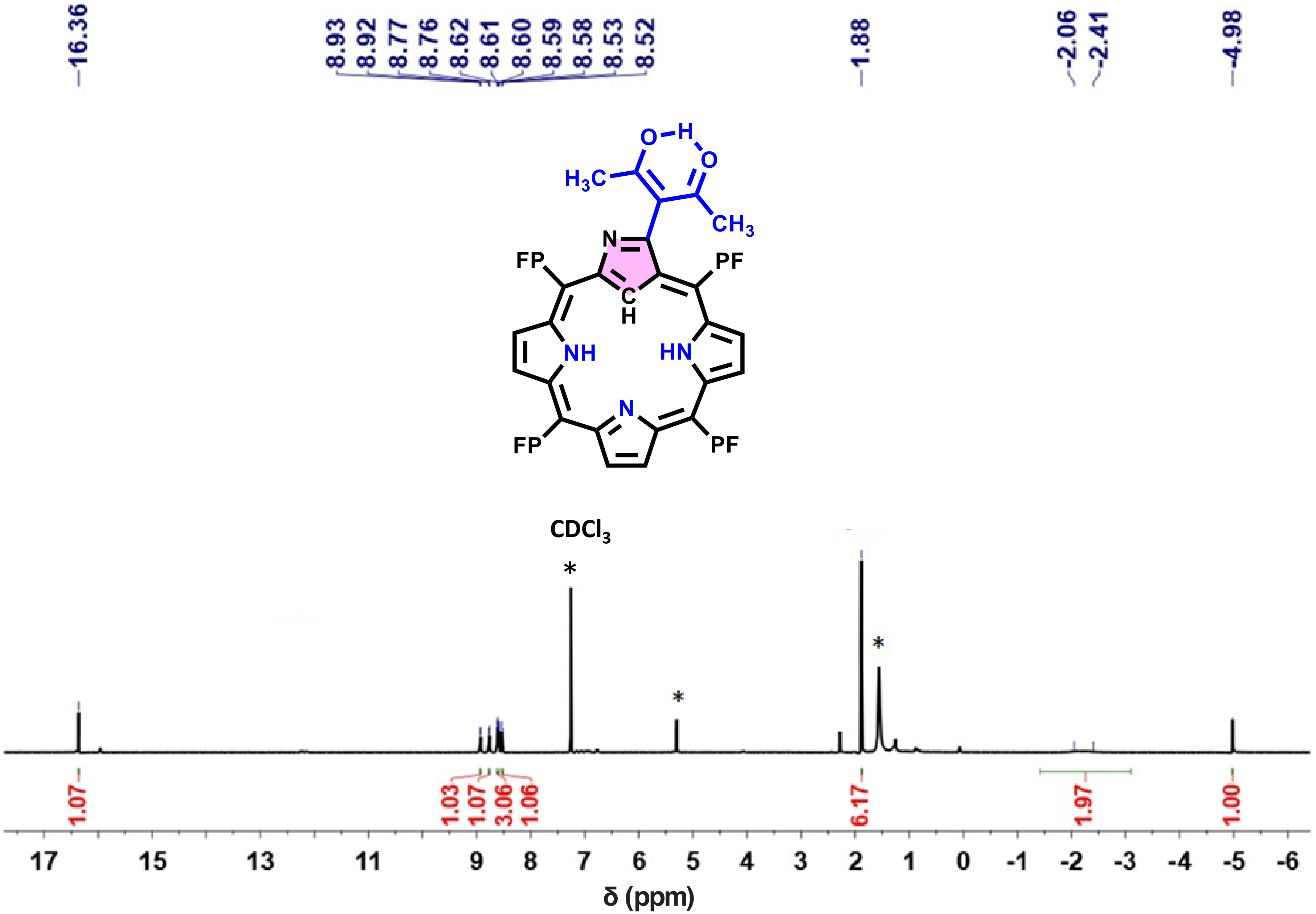

Figure S4 .^{1}H NMR (400 MHz) spectrum of the compound **2a** recorded in $CDCl_3$. The Peaks with * are due to solvent impurities.

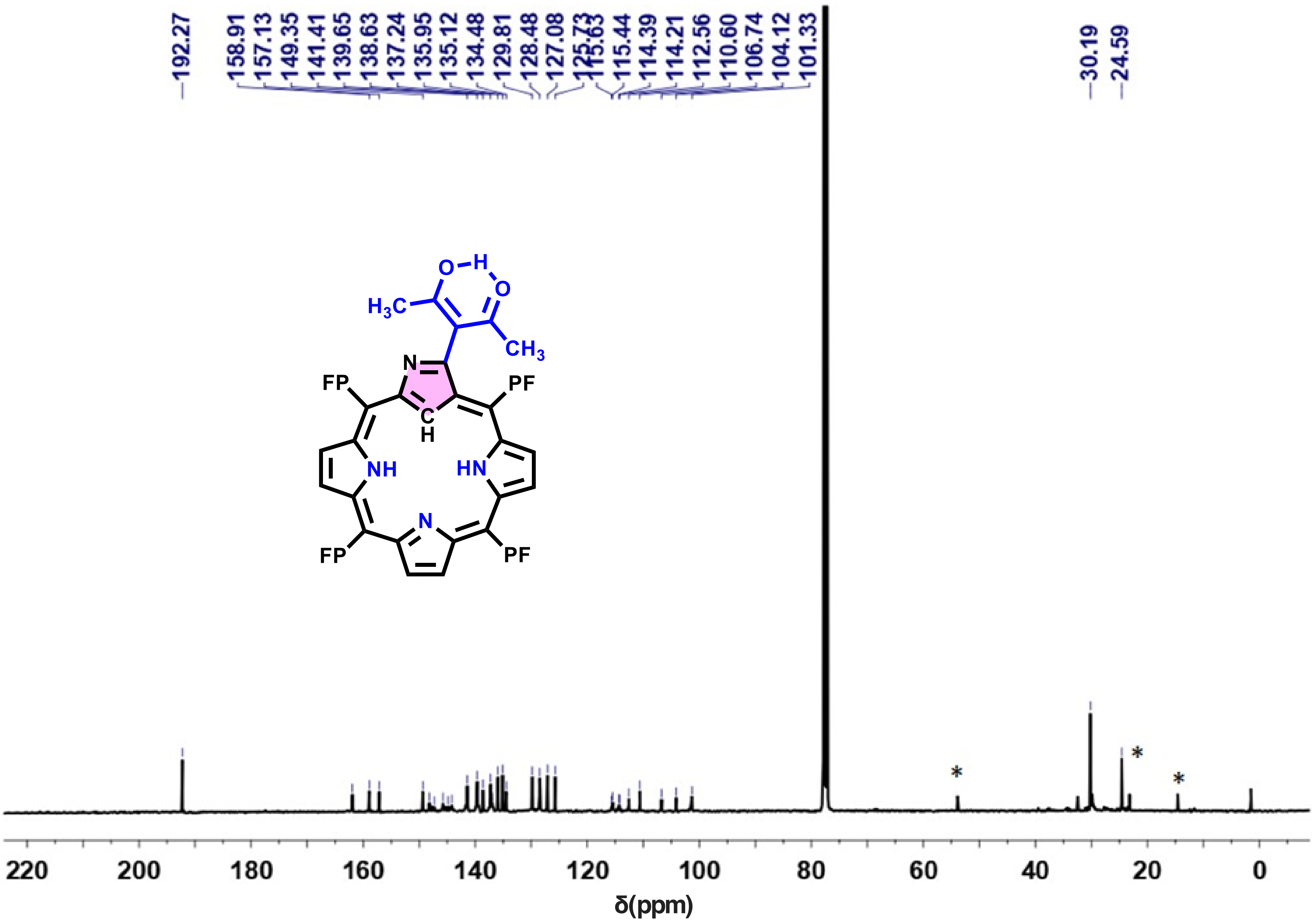

Figure S5. $^{13}C\{^{1}H\}$ NMR (101 MHz) spectrum of the compound **2a** recorded in $CDCl_3$. The Peaks with * are due to solvent impurities.

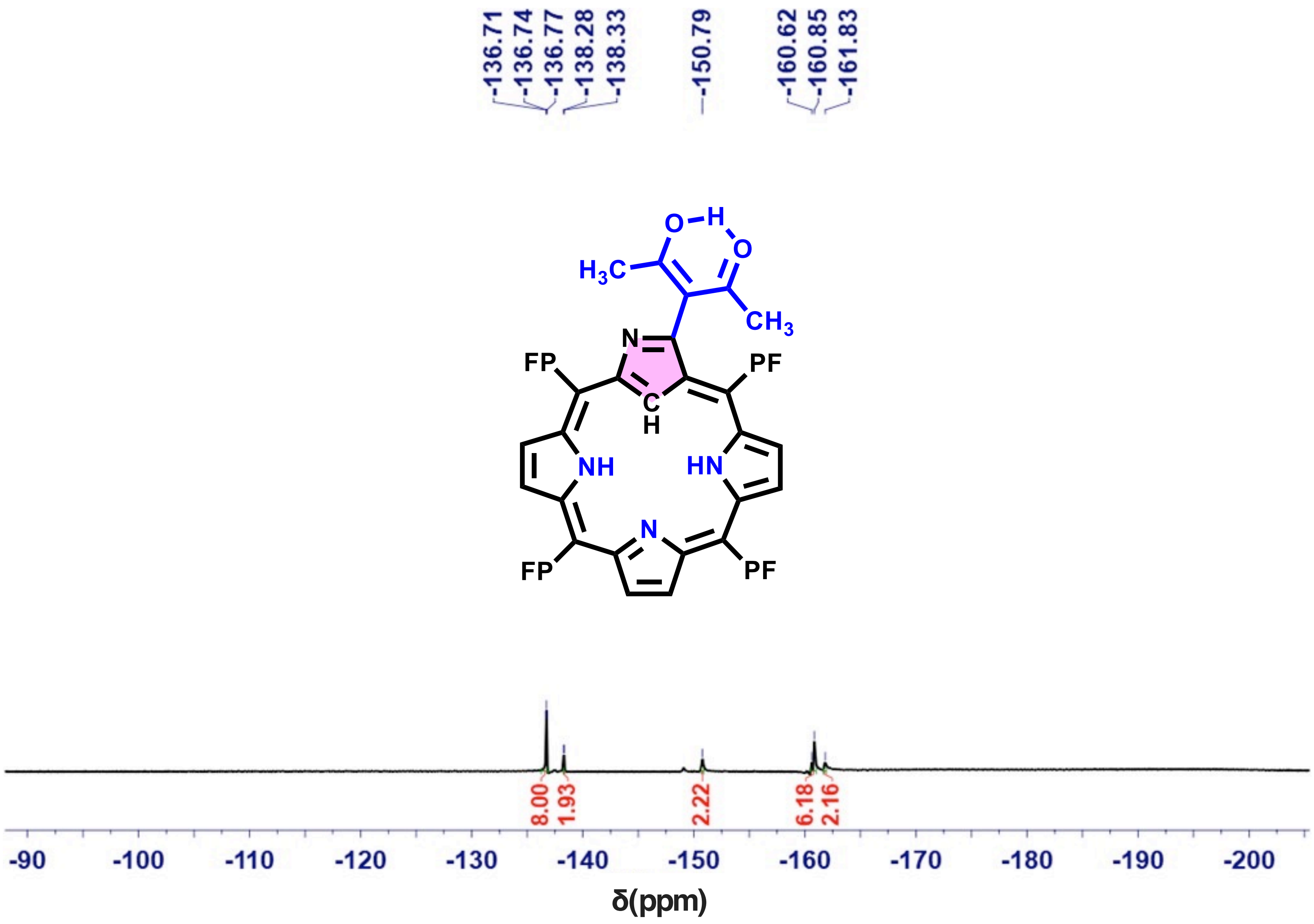

Figure S6. ^{19}F NMR (376 MHz) spectrum of the compound **2a** recorded in $CDCl_3$.

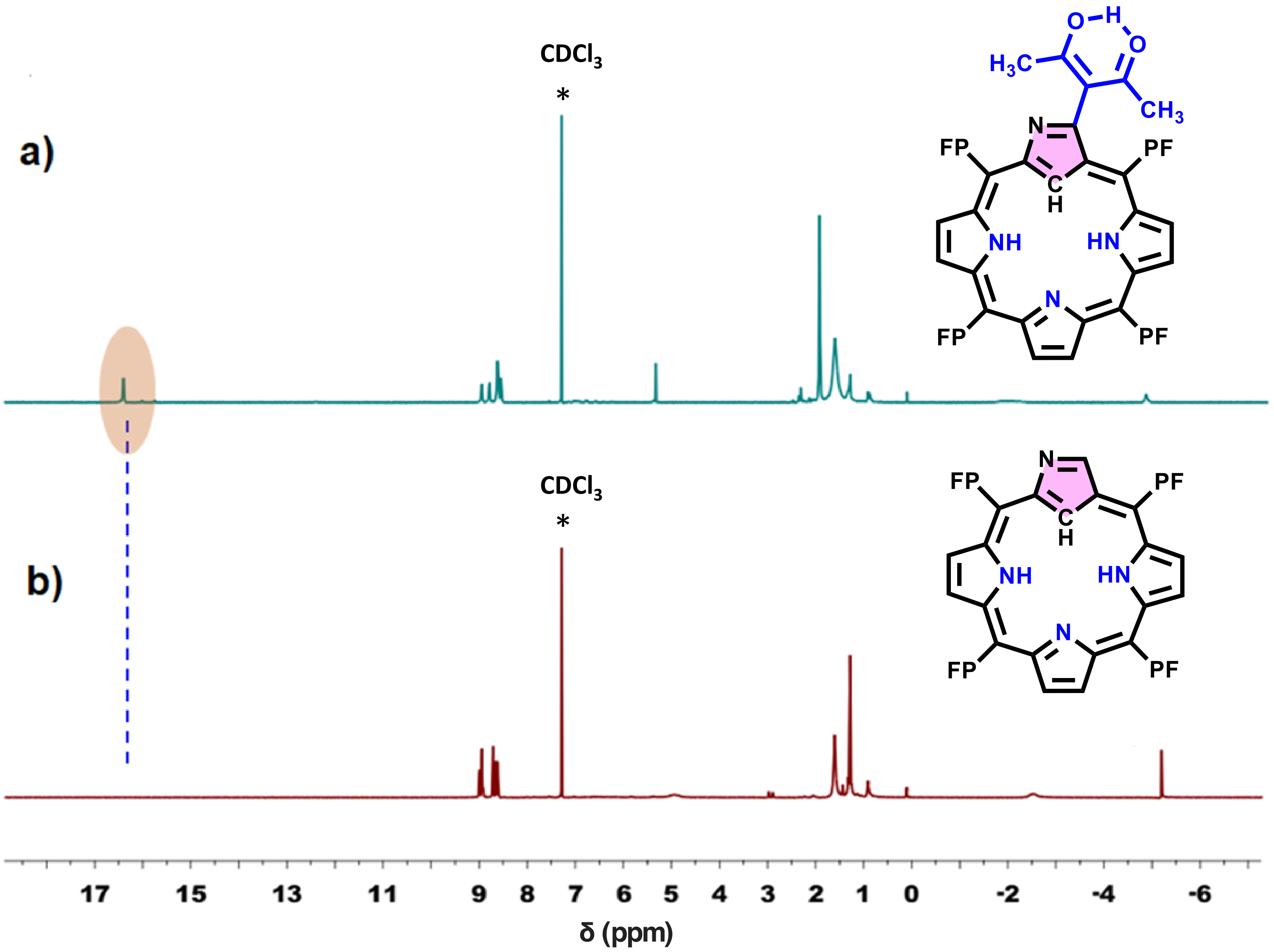

Figure S7. Comparison ^{1}H NMR (400 MHz) spectra of compound **2a** (a) and compound **1** (b) recorded in $CDCl_3$.

Spectrum

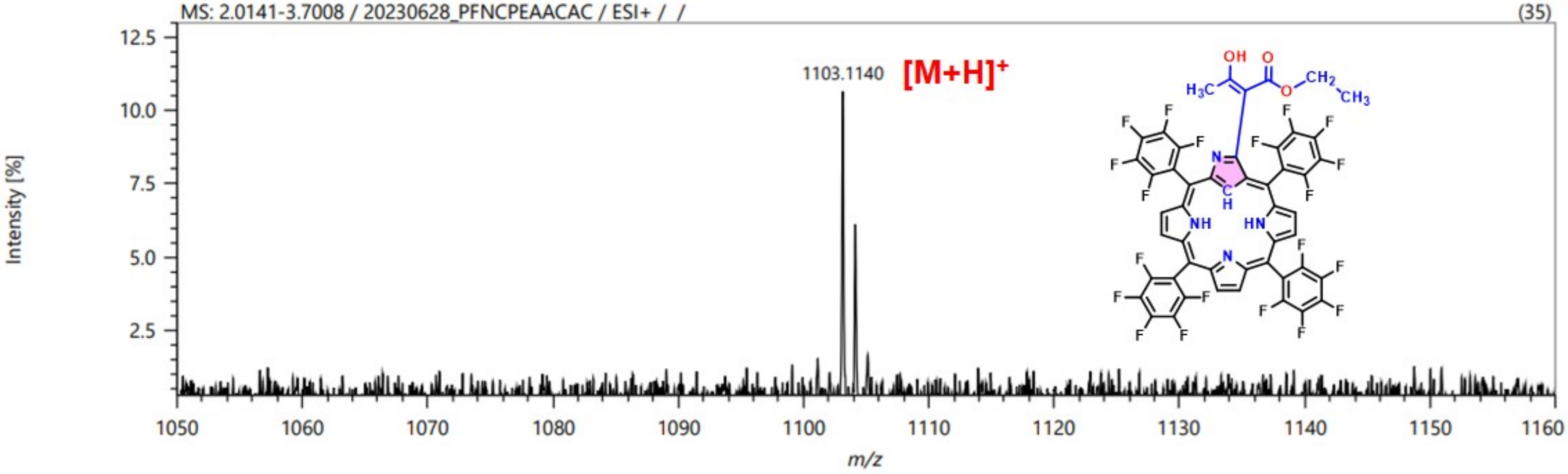

Elemental Composition

Parameters	
Tolerance:	±10.00 ppm
Electron:	Odd/Even
Charge:	+1
DBE:	-99.9 - 999.0

Elements Set 1:

Symbol	C	H	O	N	F
Min	0	0	3	4	20
Max	1000	4000	3	4	20

Results

Mass	Formula	Calculated Mass	Mass Difference [mDa]	Mass Difference [ppm]	DBE
1103.11402	C50 H19 N4 O3 F20	1103.11323	0.79	0.72	33.5

Figure S8. HR mass spectrum of the compound **2b.**

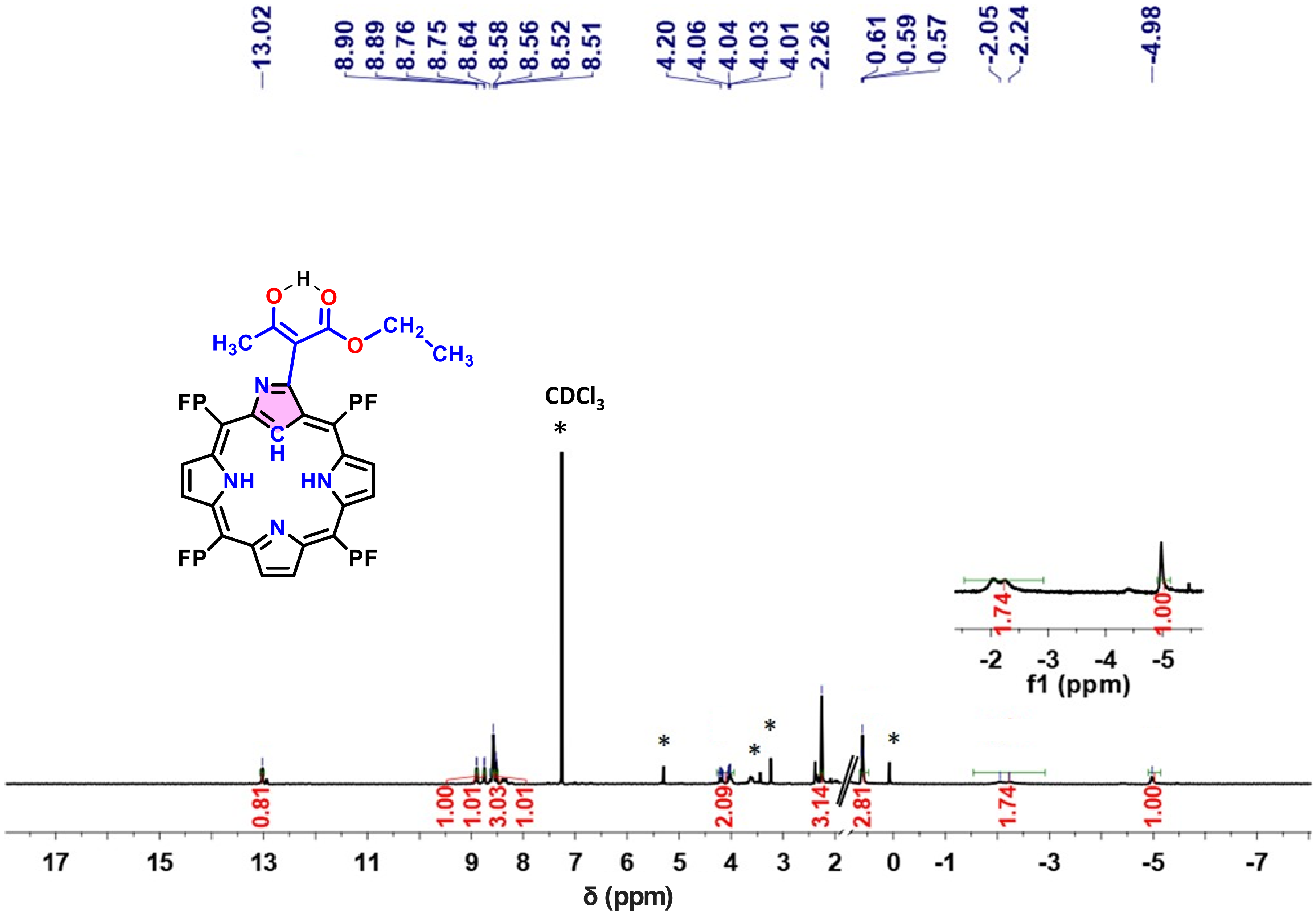

Figure S9. ^{1}H NMR (400 MHz) spectrum of the compound **2b** recorded in $CDCl_3$. The Peaks with * are due to solvent impurities.

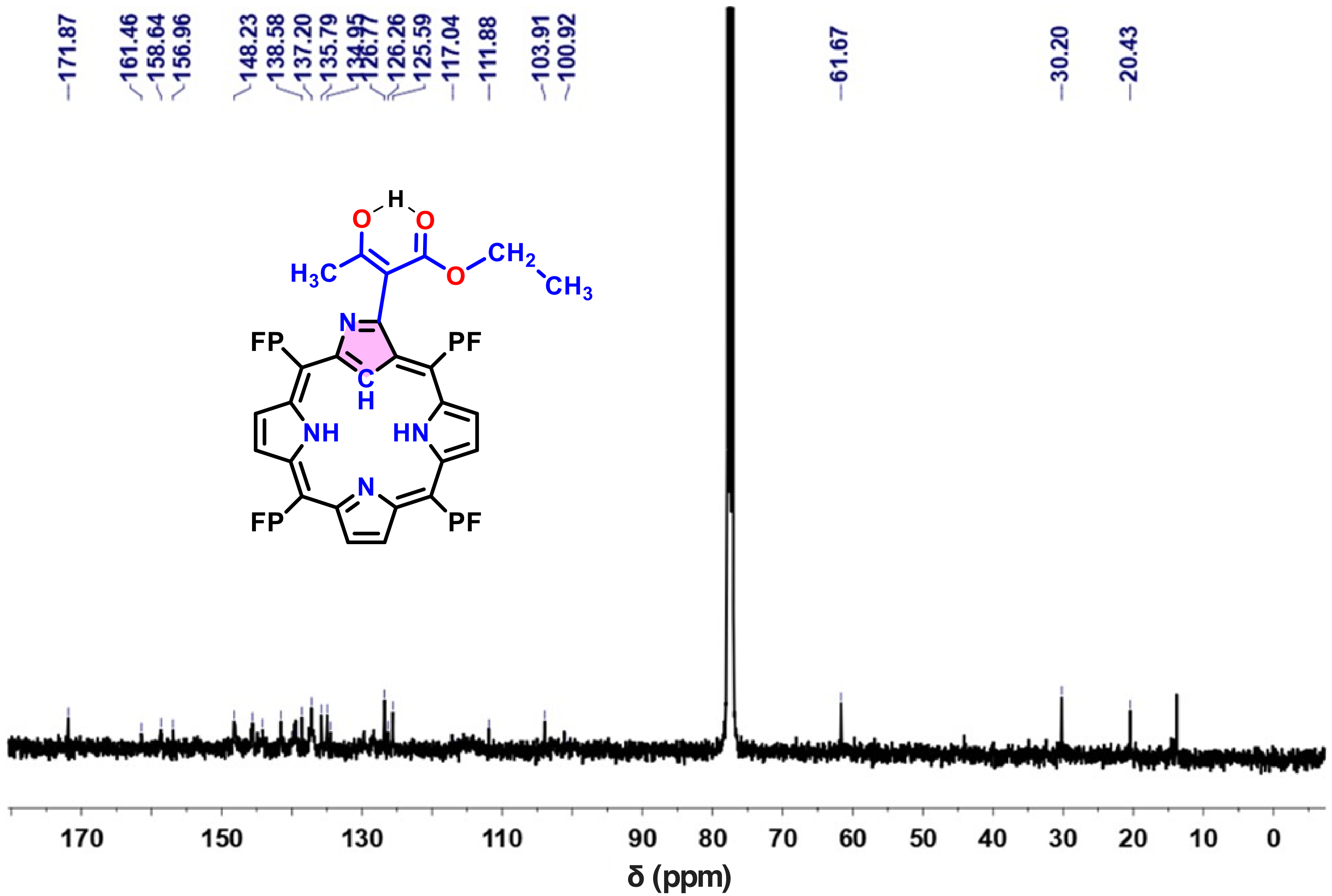

Figure S10. $^{13}C\{^{1}H\}$ NMR (101 MHz) spectrum of the compound **2b** recorded in $CDCl_3$. The Peaks with * are due to solvent impurities.

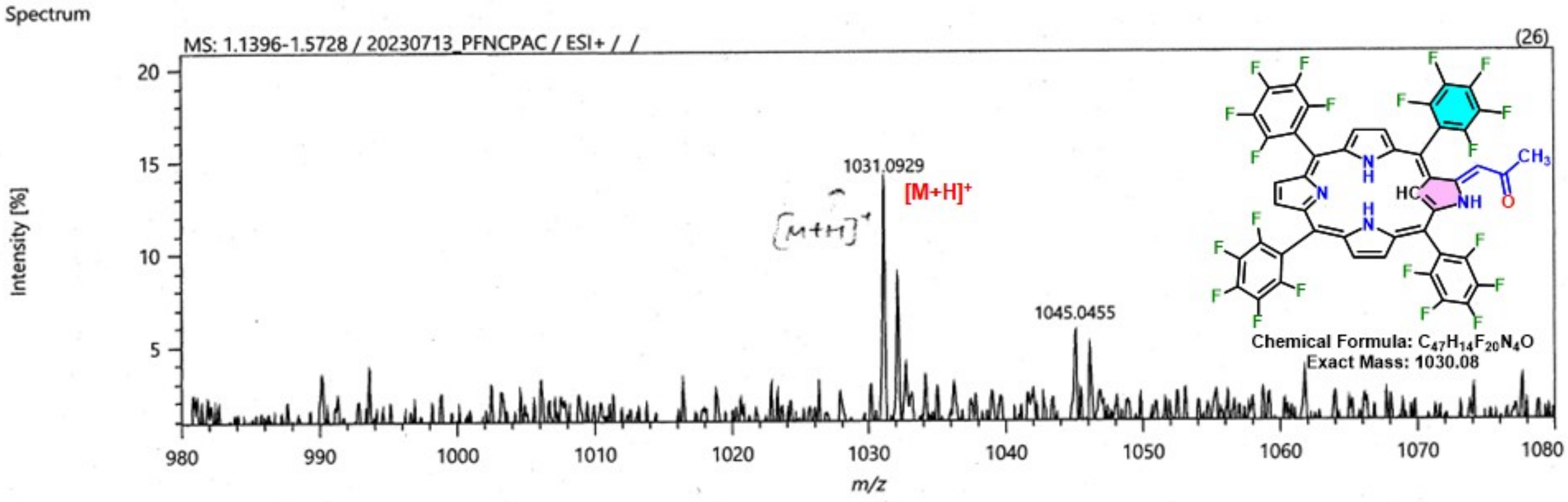

Elemental Composition

Parameters		Elements Set 2:					
Tolerance:	±10.00 ppm	Symbol	C	H	O	N	F
Electron:	Odd/Even	Min	0	0	1	4	20
Charge:	+1	Max	1000	4000	1	4	20
DBE:	-99.9 - 999.0						

Results

Mass	Formula	Calculated Mass	Mass Difference [mDa]	Mass Difference [ppm]	DBE
1031.09294	C47 H15 N4 O F20	1031.09210	0.84	0.81	32.5

Figure S11. HR mass spectrum of the compound **3a**.

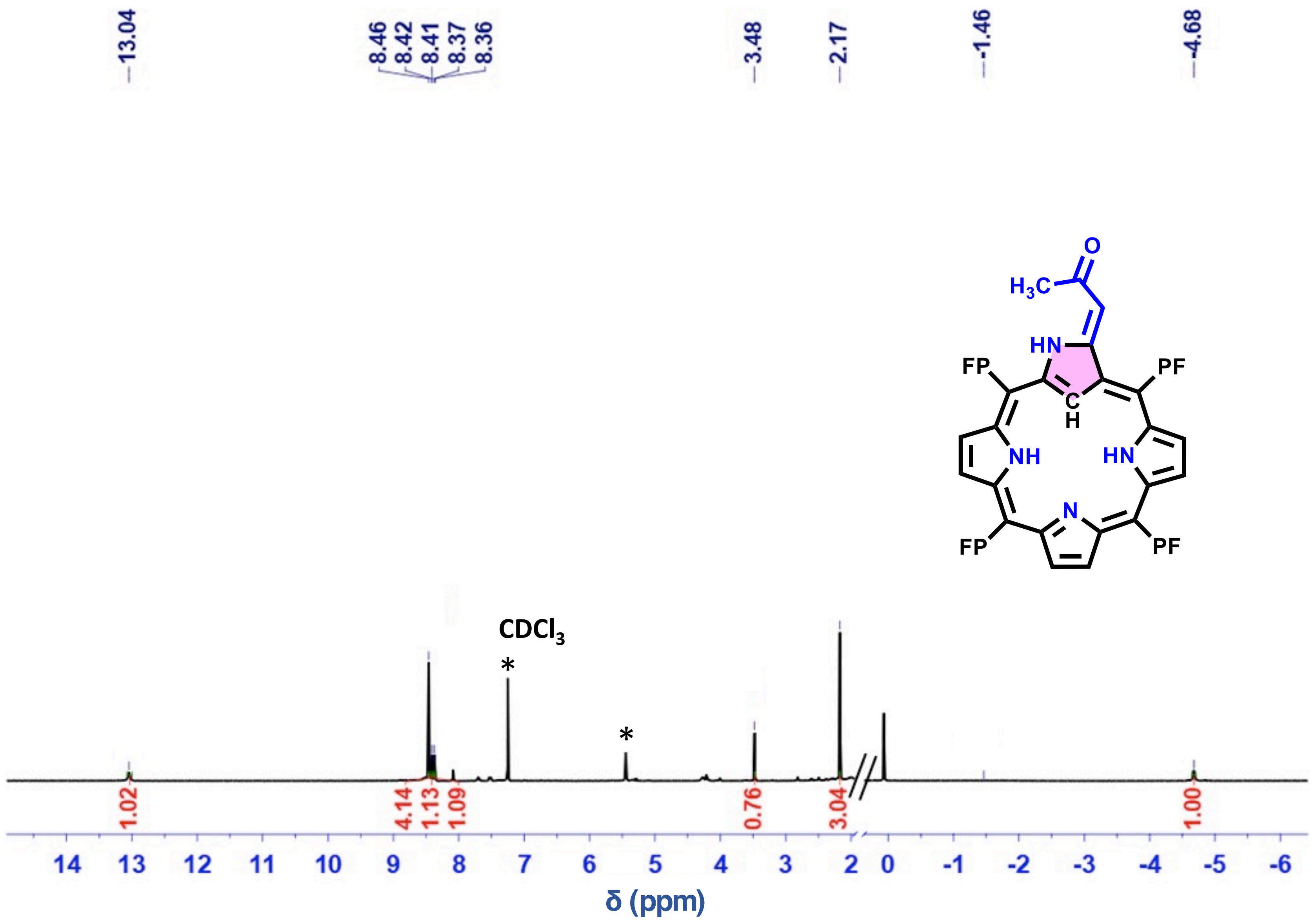

Figure S12. ^{1}H NMR (400 MHz) spectrum of the compound **3a** recorded in $CDCl_3$. The Peaks with * are due to solvent impurities.

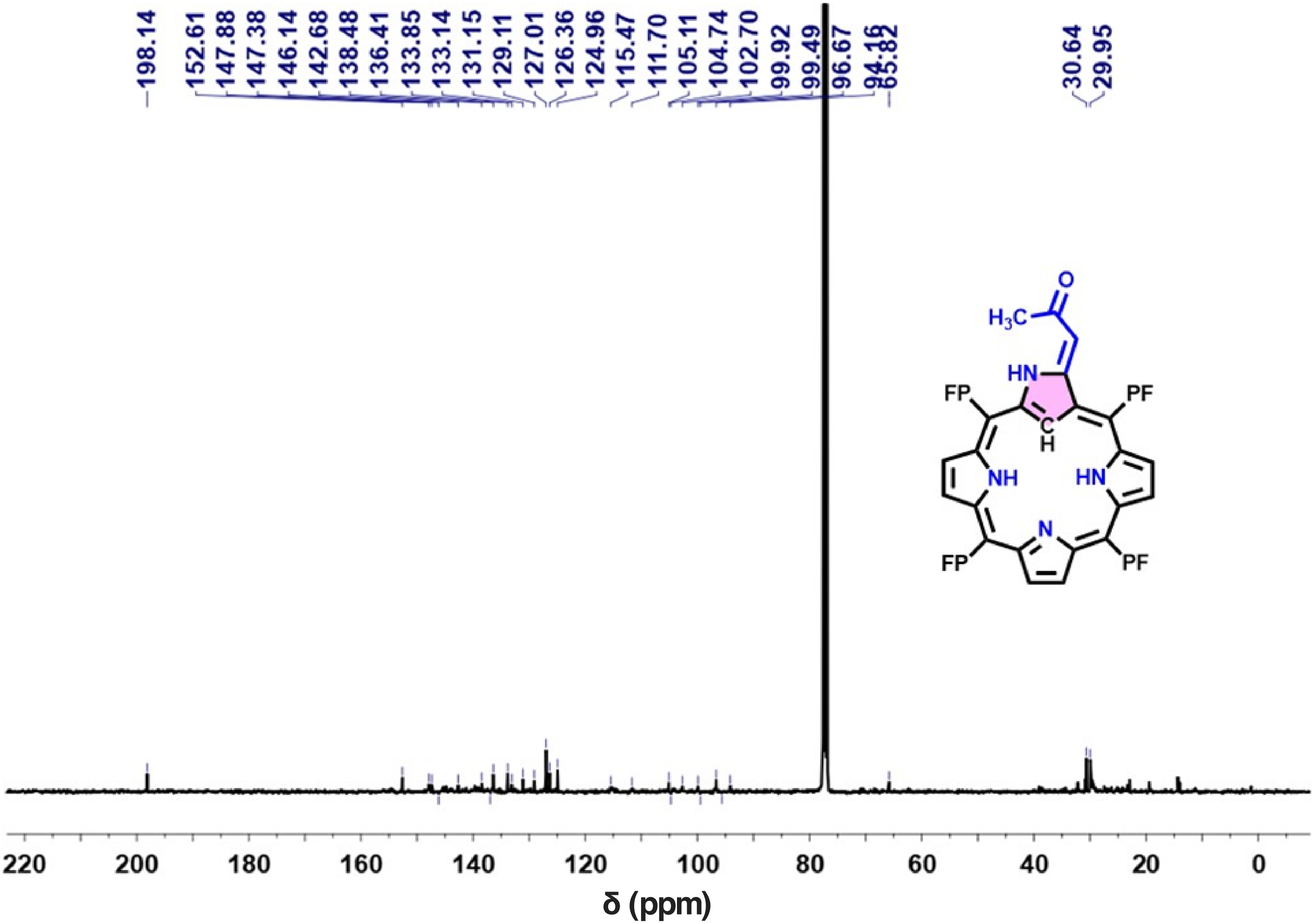

Figure S13. $^{13}C\{^{1}H\}$ NMR (101 MHz) spectrum of the compound **3a** recorded in $CDCl_3$.

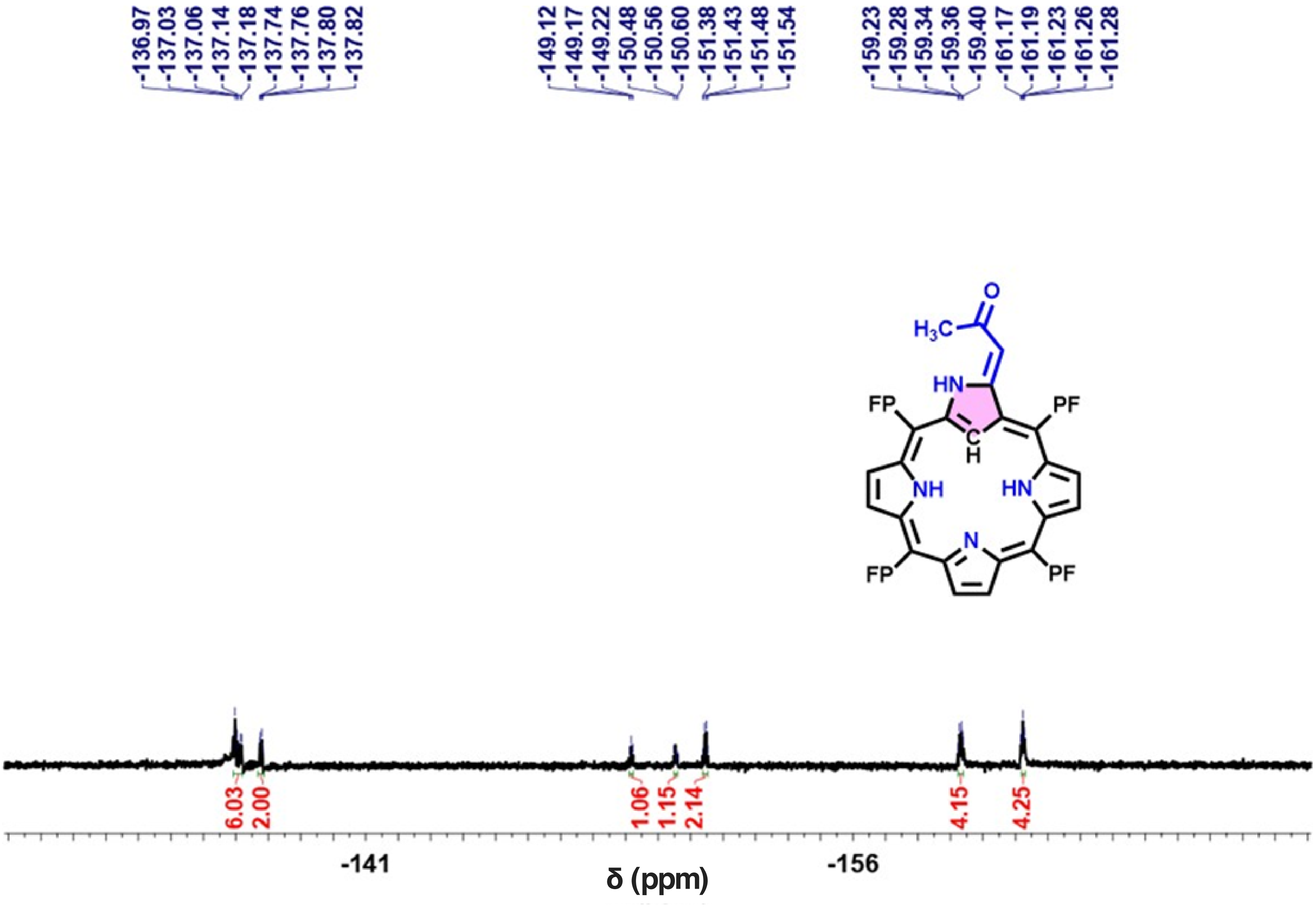

Figure S14. ^{19}F NMR (376 MHz) spectrum of the compound **3a** recorded in $CDCl_3$.

Spectrum

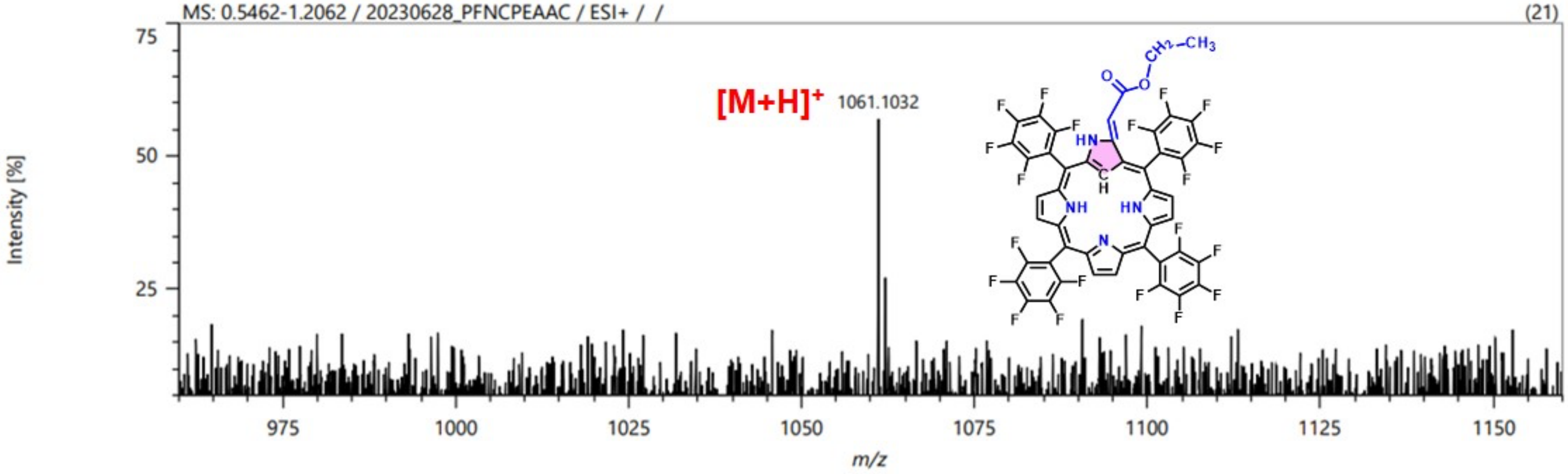

Elemental Composition

Parameters	
Tolerance:	±10.00 ppm
Electron:	Odd/Even
Charge:	+1
DBE:	-99.9 - 999.0

Elements Set 1:

Symbol	C	H	O	N	F
Min	0	0	2	4	20
Max	1000	4000	2	4	20

Results

Mass	Formula	Calculated Mass	Mass Difference [mDa]	Mass Difference [ppm]	DBE
1061.10324	C48 H17 N4 O2 F20	1061.10267	0.57	0.54	32.5

Figure S15. HR mass spectrum of the compound **3b**.

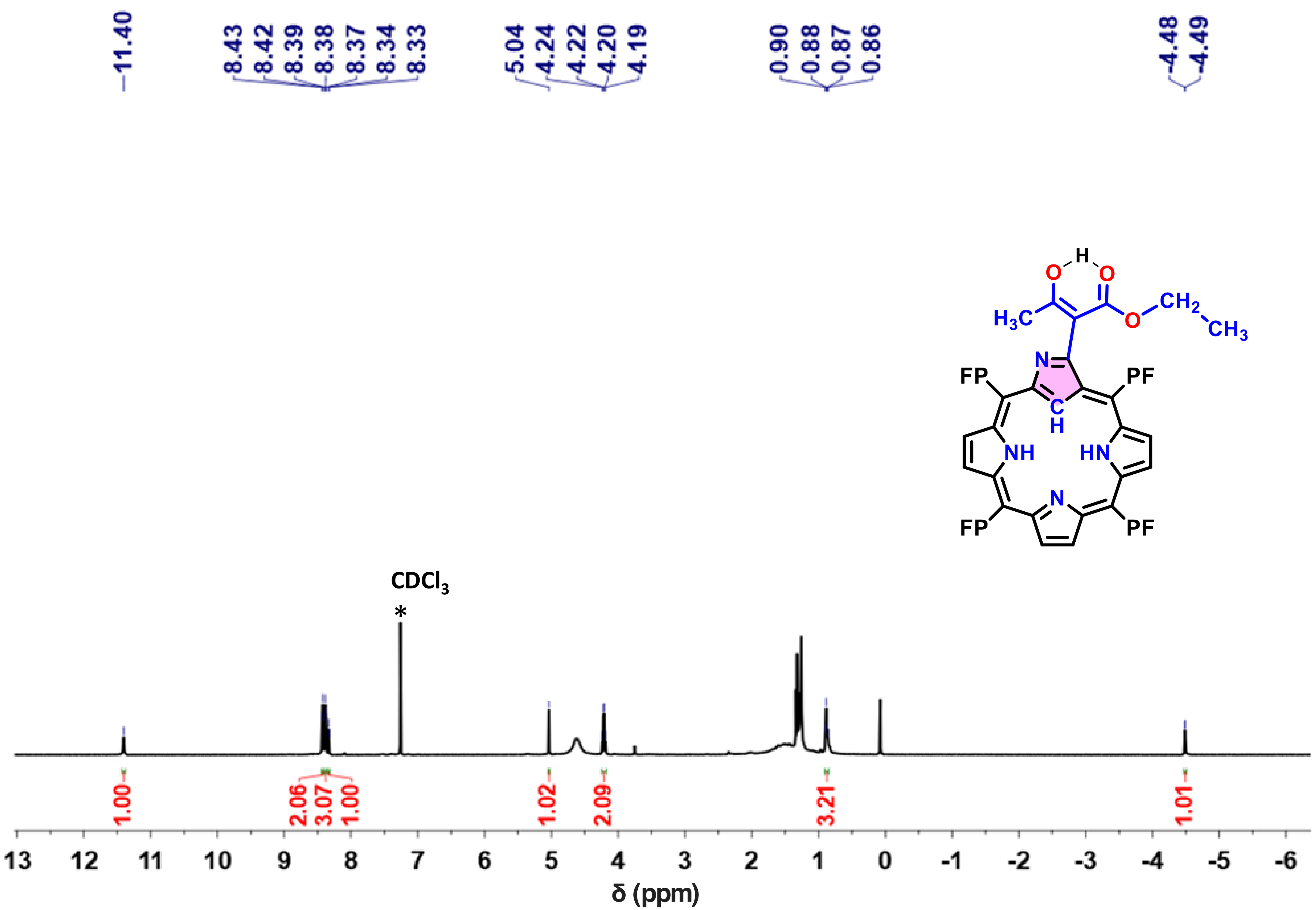

Figure S16. ^{1}H NMR (400 MHz) spectrum of the compound **3b** recorded in $CDCl_3$. The Peaks with * are due to solvent impurities.

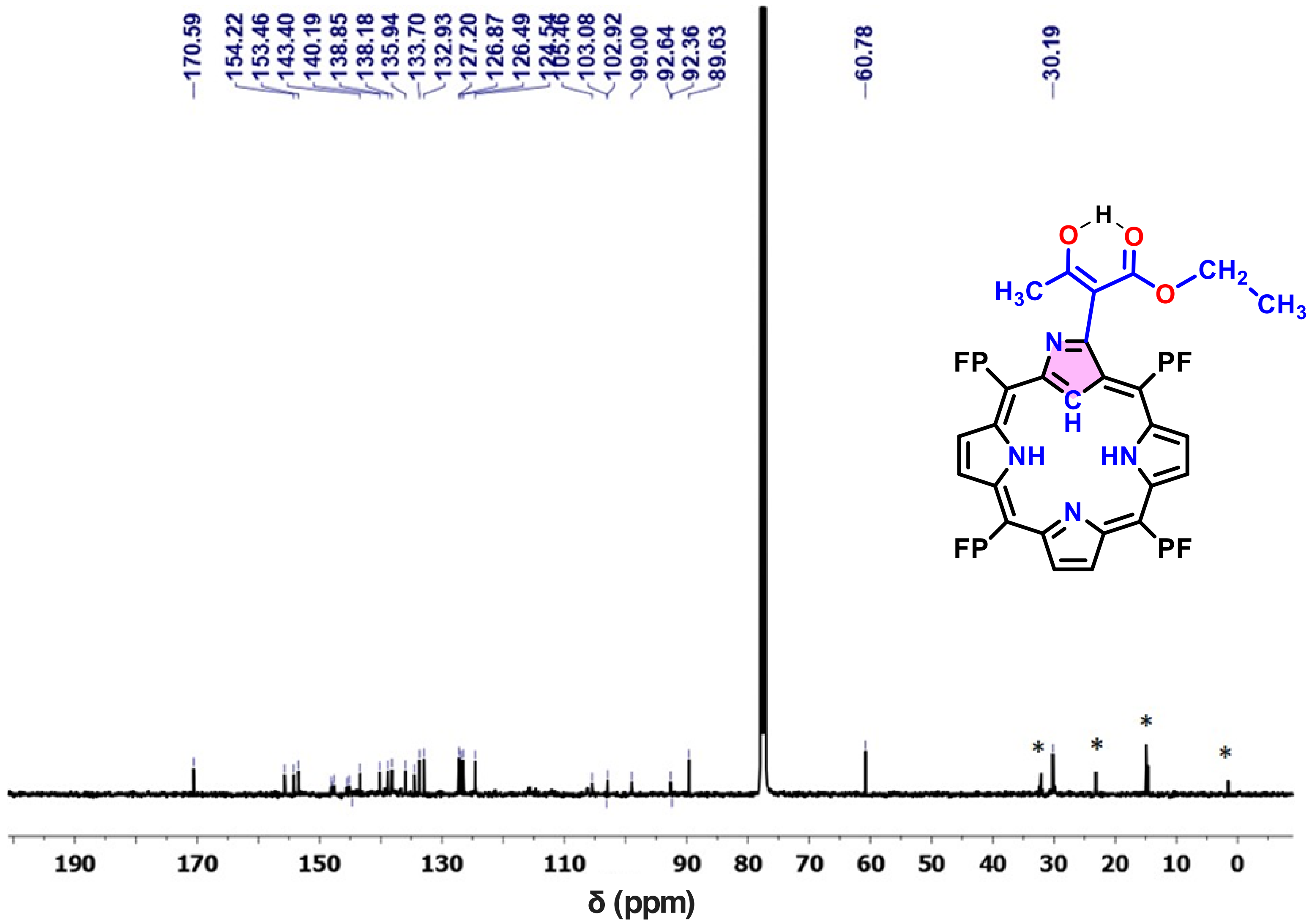

Figure S17. $^{13}C\{^1H\}$ NMR (101 MHz) spectrum of the compound **3b** recorded in $CDCl_3$. The Peaks with * are due to solvent impurities.

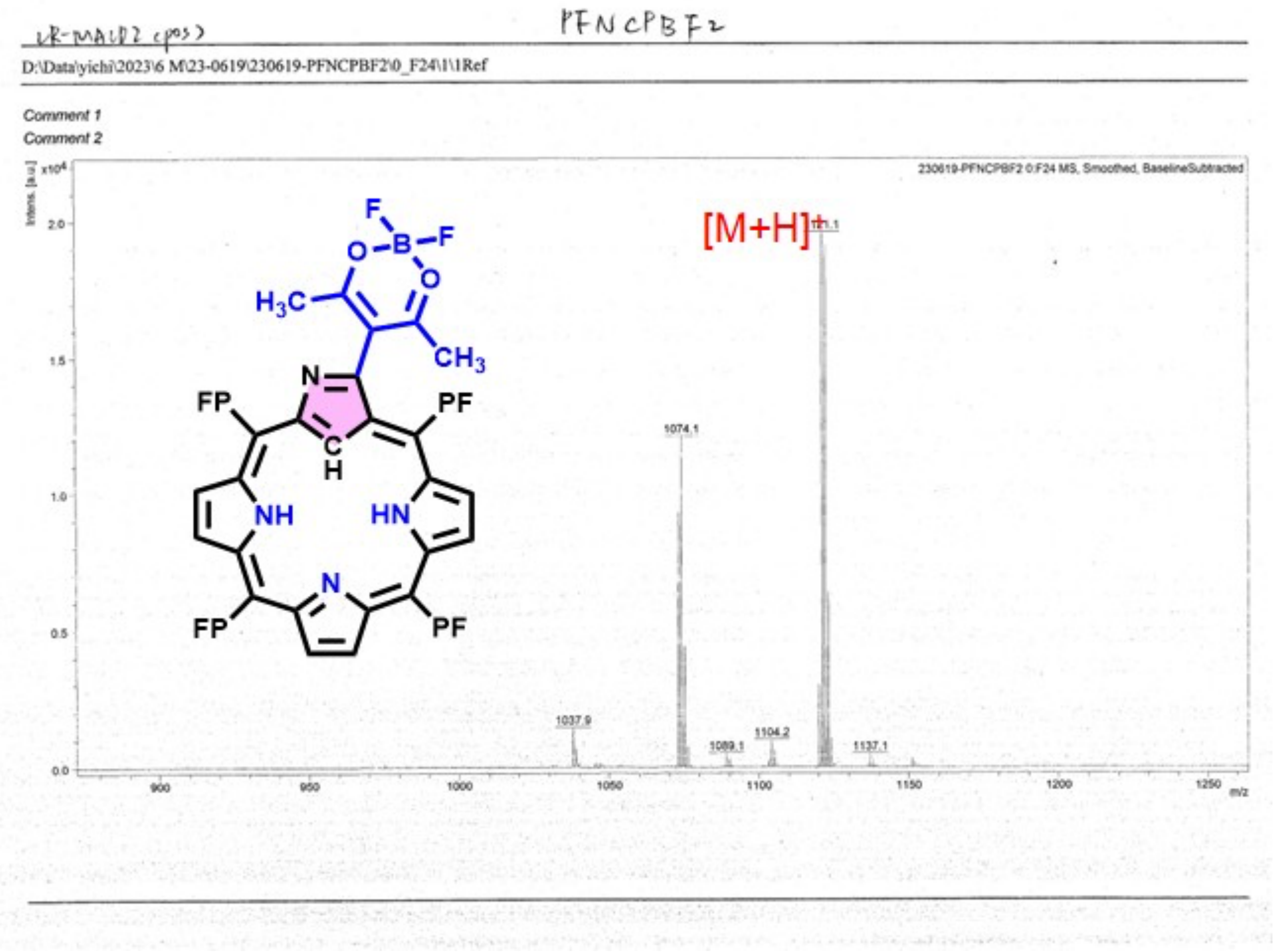

Figure S18. MALDI TOF mass spectrum of the compound **4**.

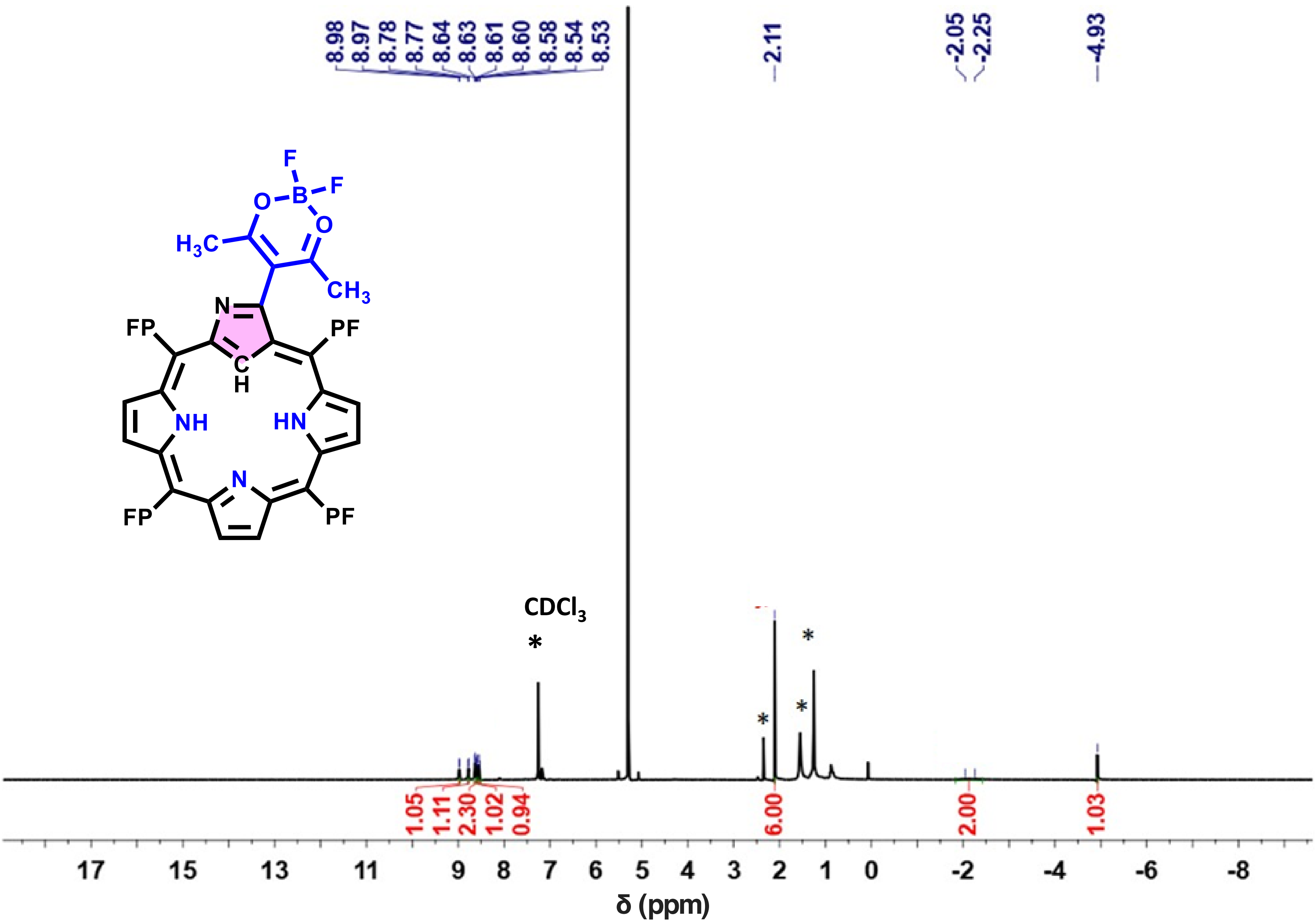

Figure S19. ^{1}H NMR (400 MHz) spectrum of compound **4** recorded in $CDCl_3$. The Peak with * is due to solvent impurity.

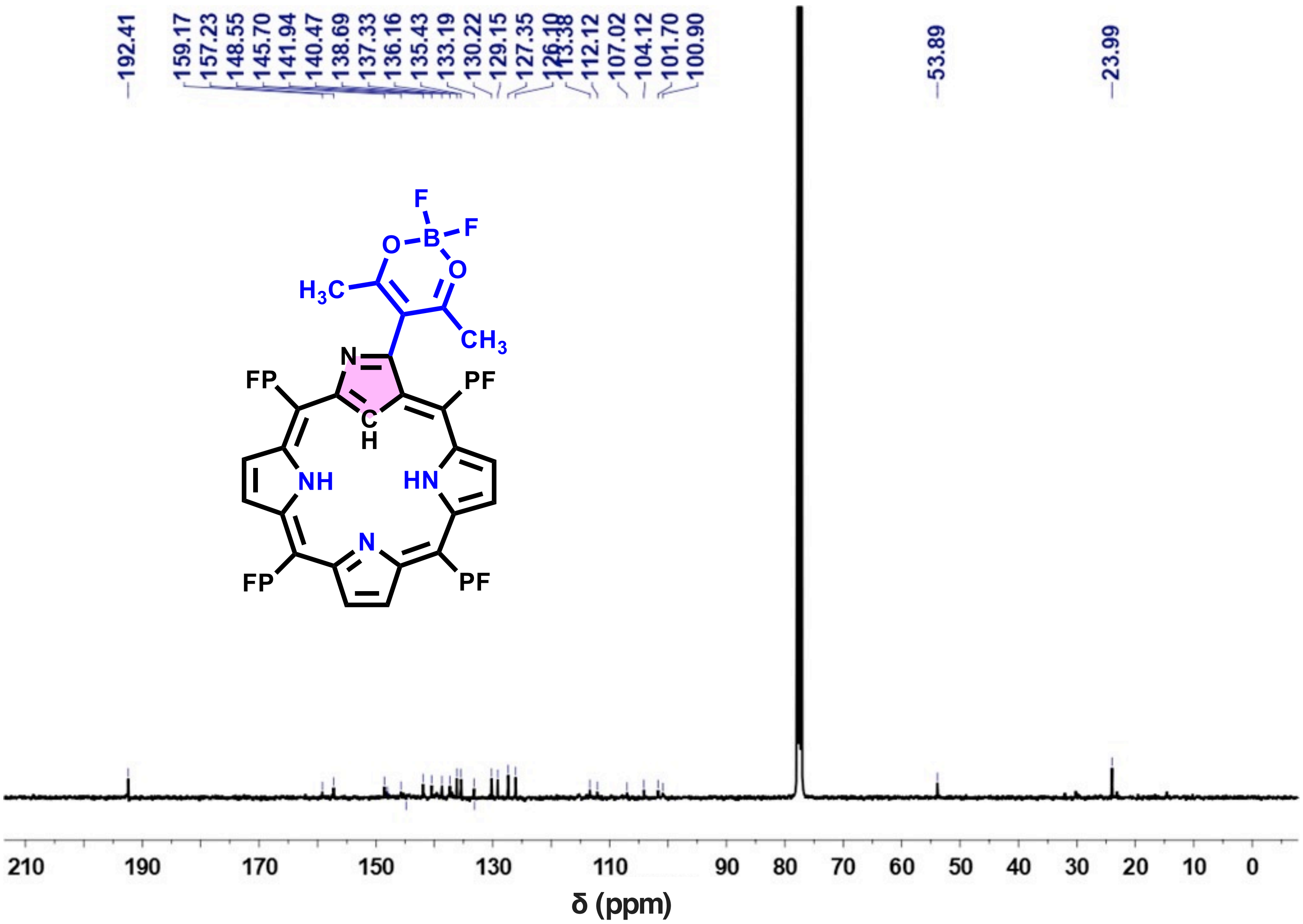

Figure S20. $^{13}C\{^{1}H\}$ NMR (101 MHz) spectrum of compound **4** recorded in $CDCl_3$. The Peak with * is due to solvent impurity.

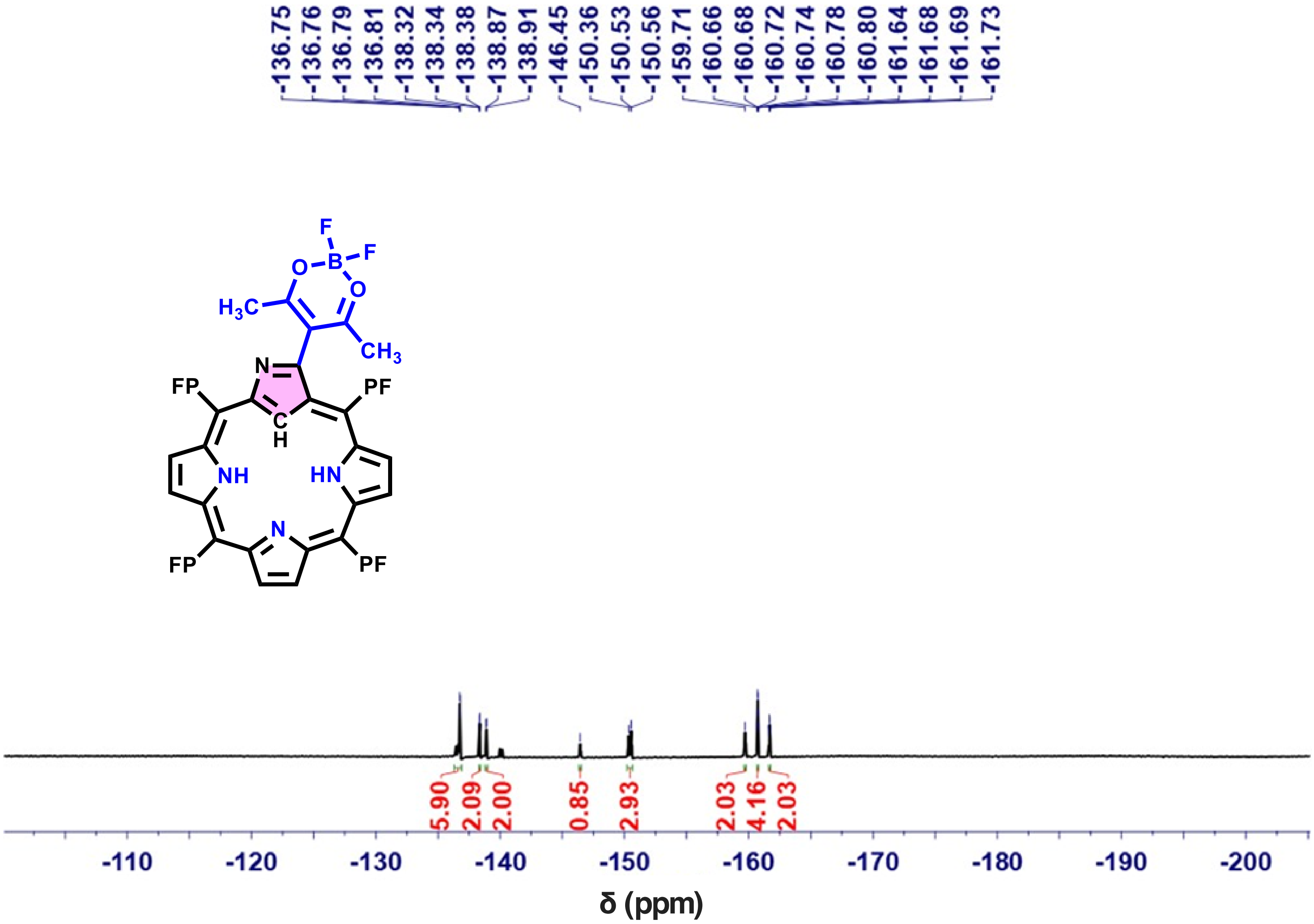

Figure S21. ^{19}F NMR (376 MHz) spectrum of compound **4** recorded in $CDCl_3$.

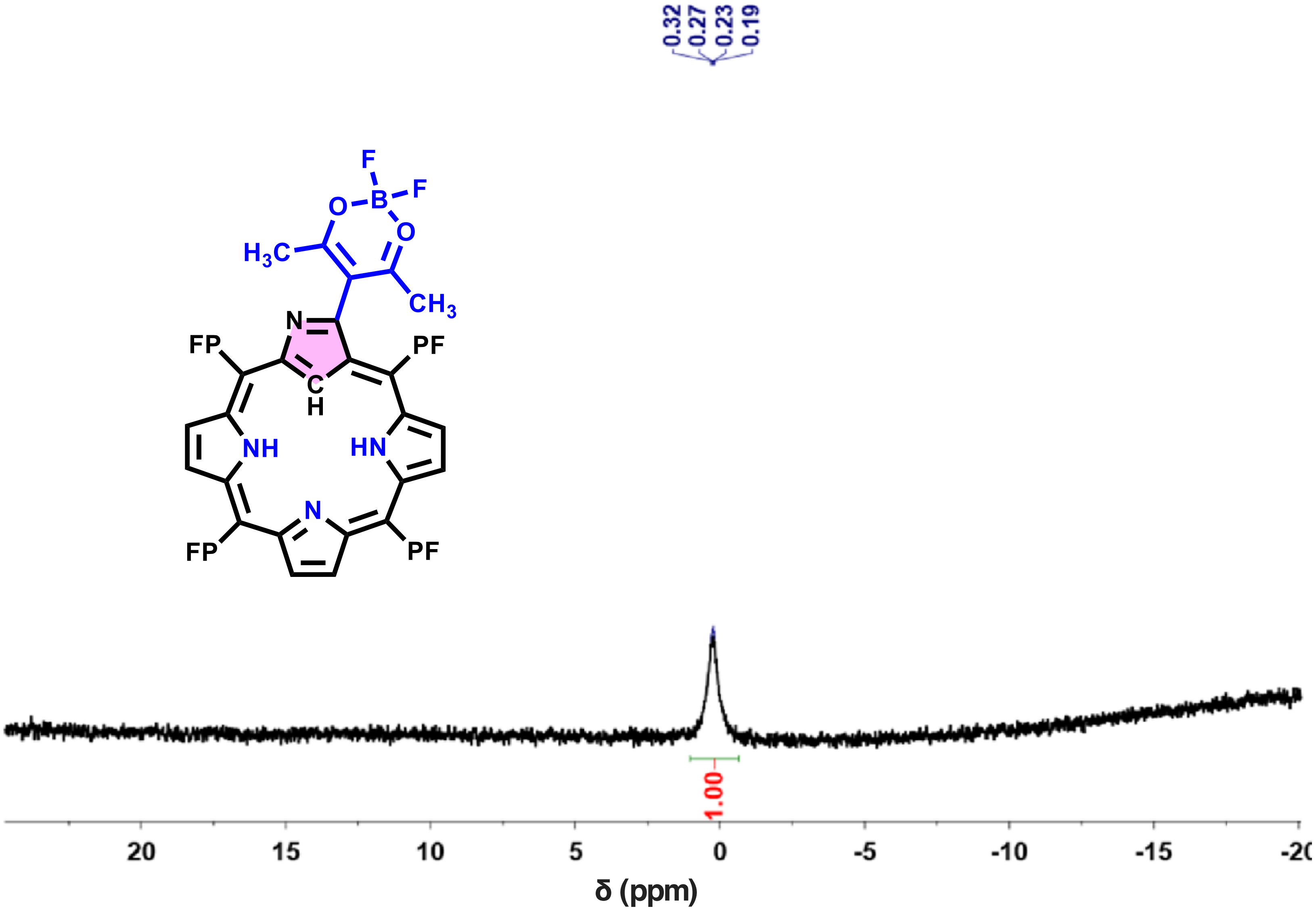

Figure S22. ^{11}B NMR (128 MHz) spectrum of compound **4** recorded in $CDCl_3$.

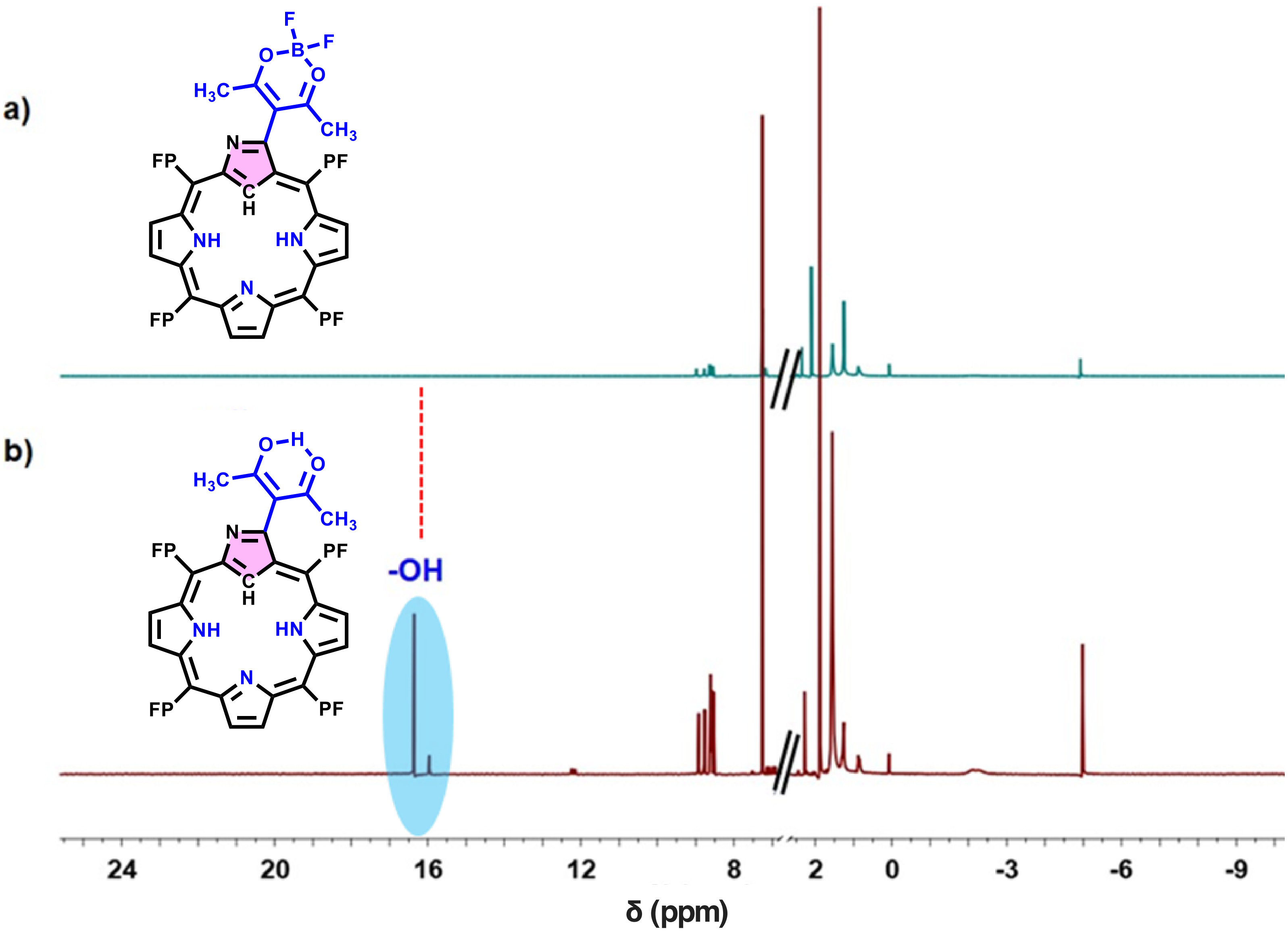

Figure S23. Comparison of ^{1}H NMR (400 MHz) spectrum of compound **4 (a)** and **2a (b)** recorded in $CDCl_3$. The Peak with * is due to solvent impurity.

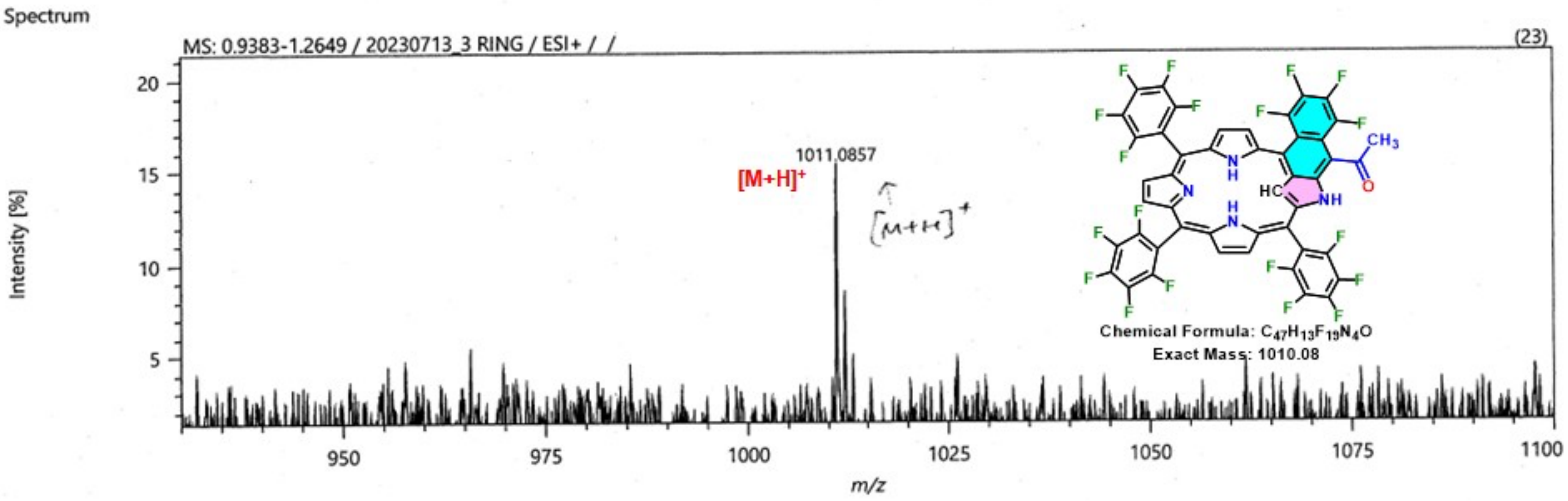

Elemental Composition

Parameters	
Tolerance:	±10.00 ppm
Electron:	Odd/Even
Charge:	+1
DBE:	-99.9 - 999.0

Elements Set 2:

Symbol	C	H	O	N	F
Min	0	0	1	4	19
Max	1000	4000	1	4	19

Results

Mass	Formula	Calculated Mass	Mass Difference [mDa]	Mass Difference [ppm]	DBE
1011.08566	C47 H14 N4 O F19	1011.08587	-0.22	-0.21	33.5

Figure S24. HR mass spectrum of the compound **5.**

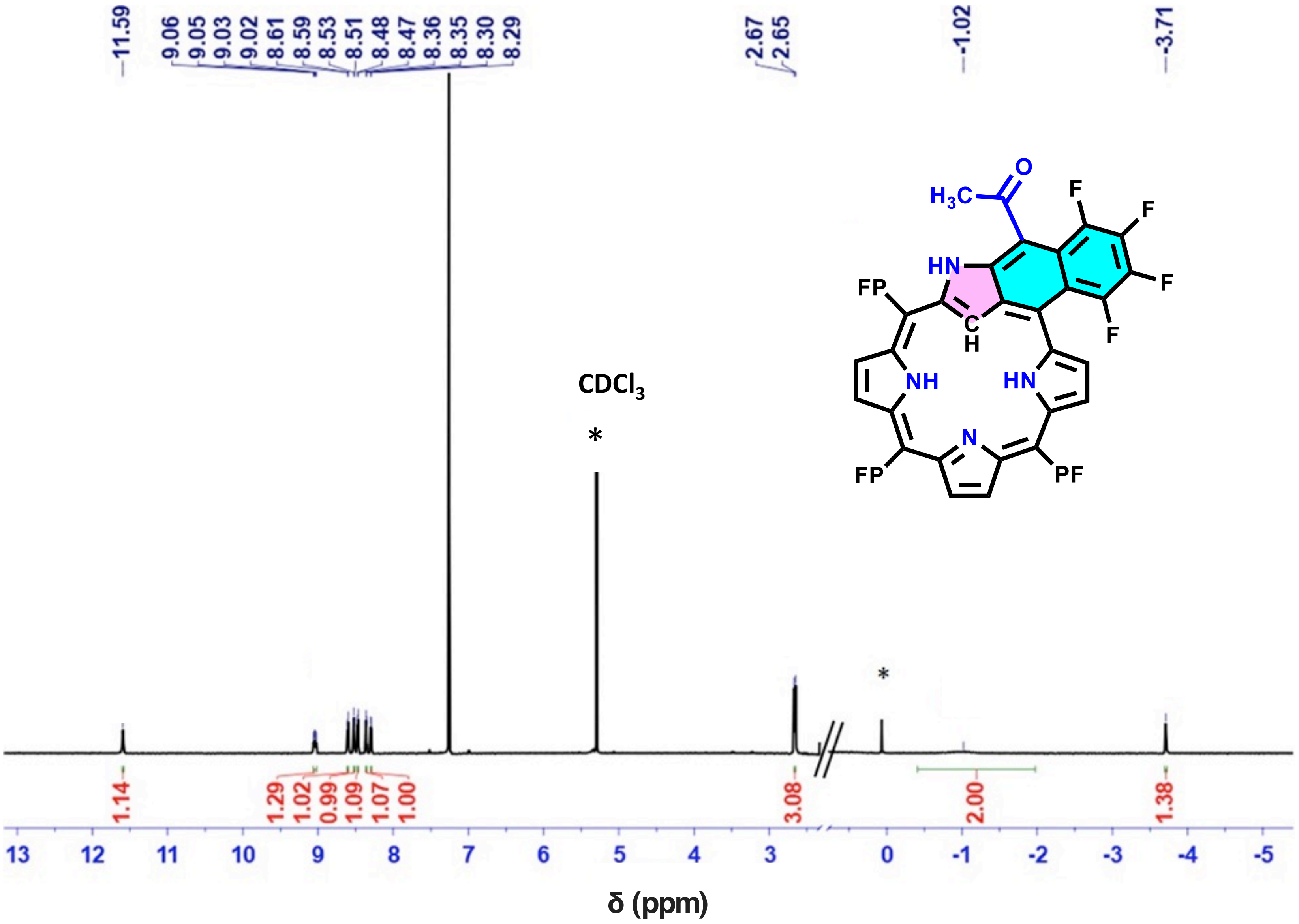

Figure S25. ^{1}H NMR (400 MHz) spectrum of the compound **5** recorded in $CDCl_3$. The Peaks with * are due to solvent impurities.

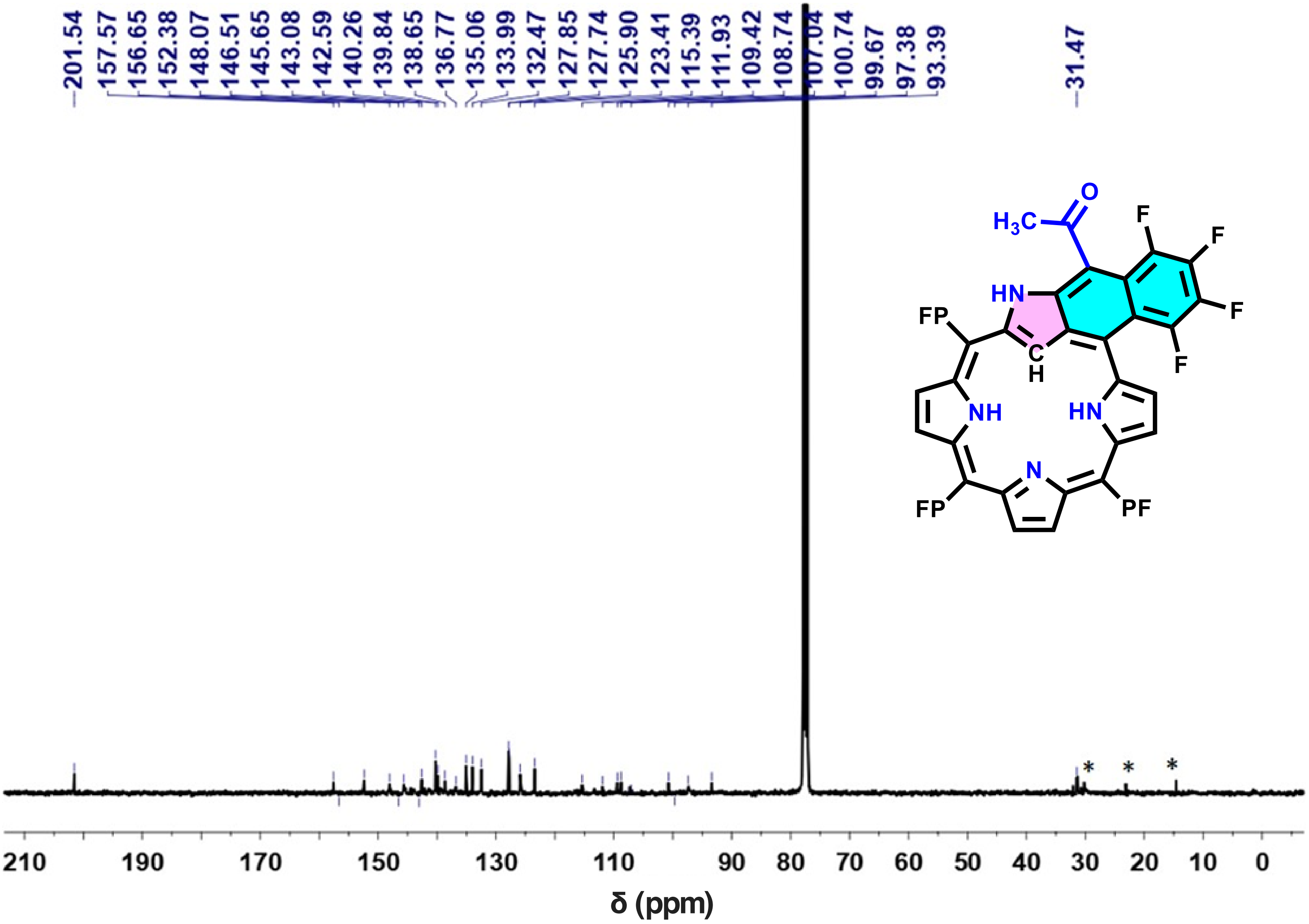

Figure S26. $^{13}C\{^{1}H\}$ NMR (101 MHz) spectrum of the compound **5** recorded in $CDCl_3$. . The Peaks with * are due to solvent impurities.

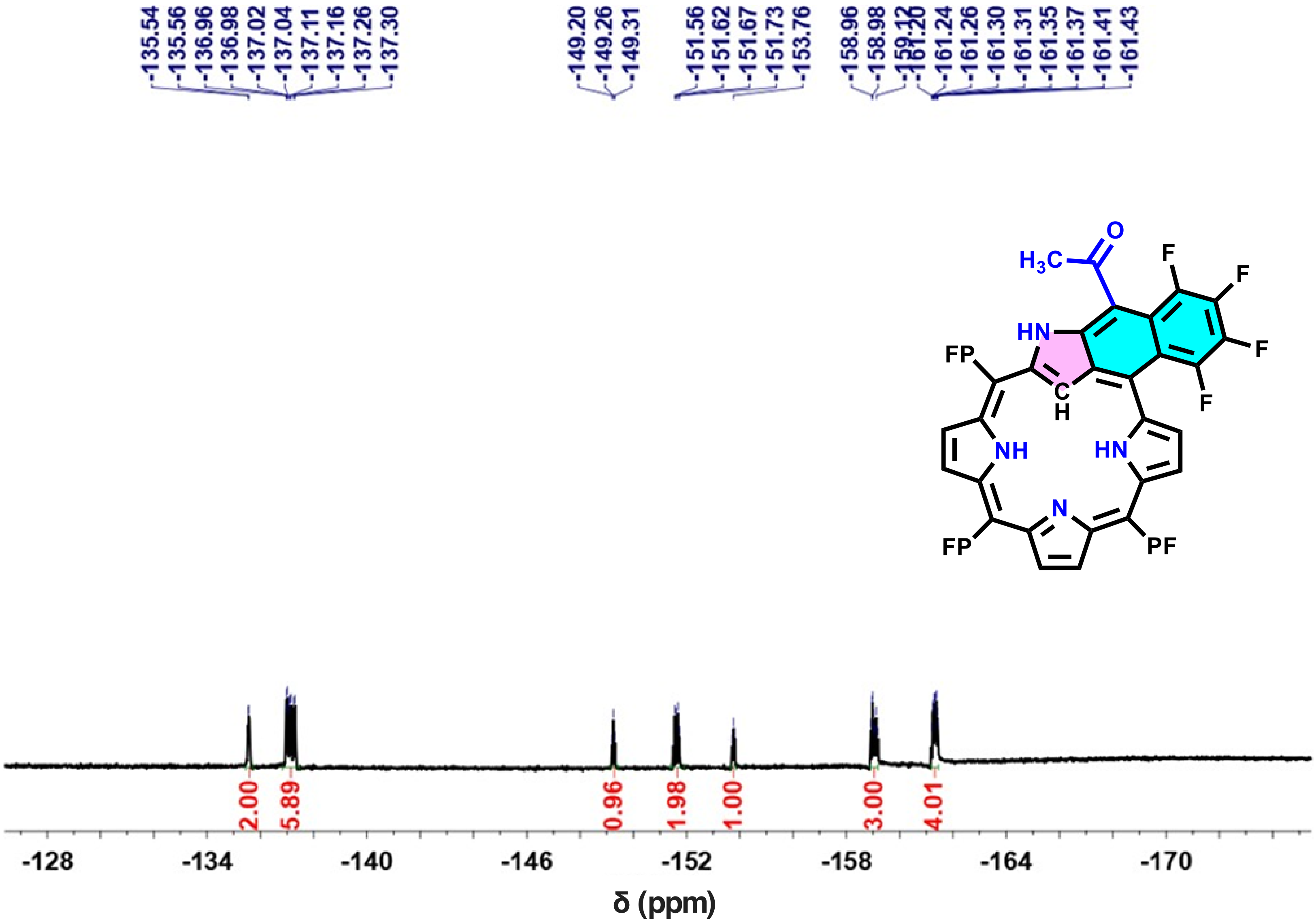

Figure S27. ^{19}F NMR (376 MHz) spectrum of the compound **5** recorded in $CDCl_3$.

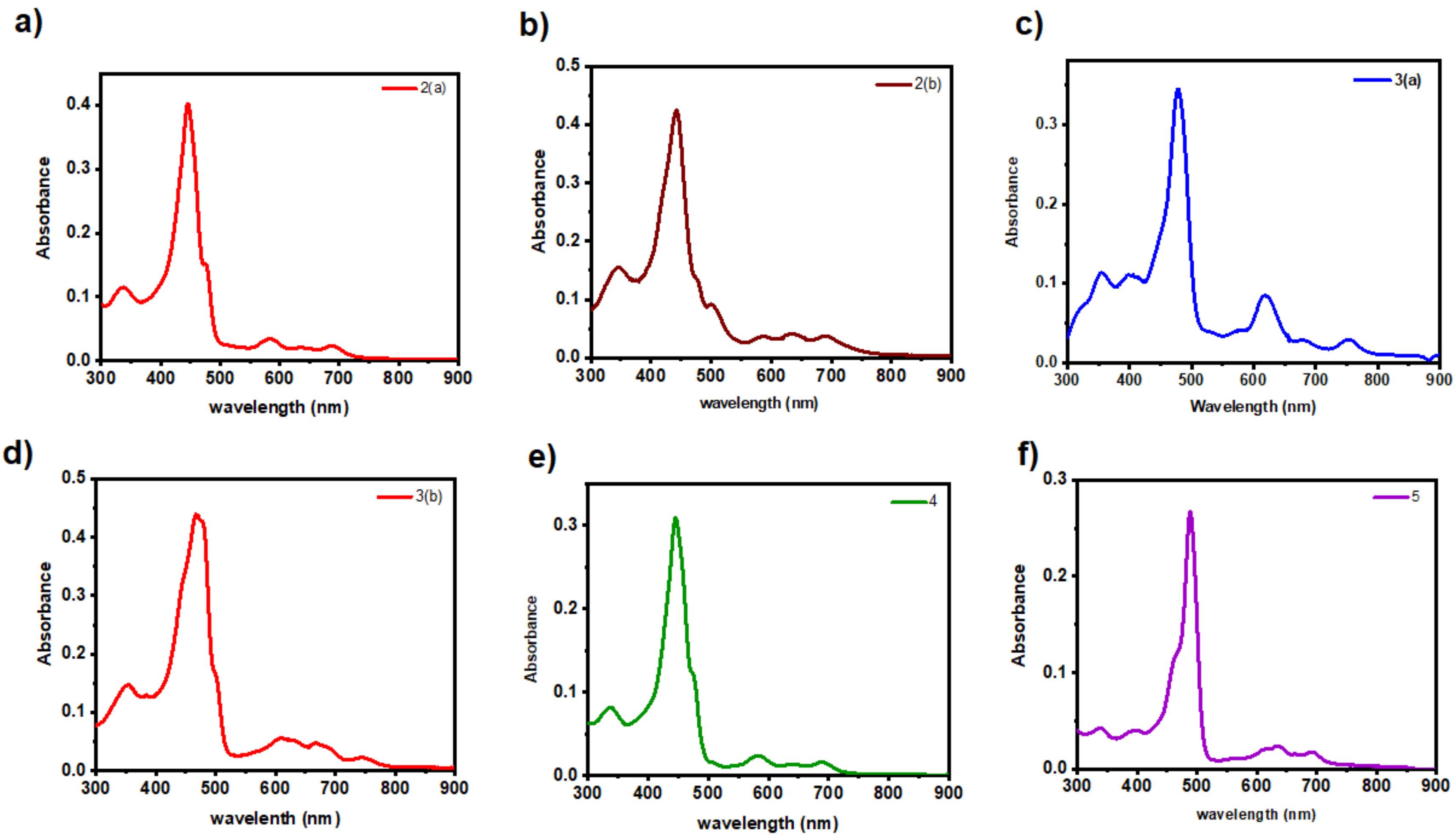

Figure S28. Absorption spectra of all the new compounds **2-5** recorded in CH_3CN.

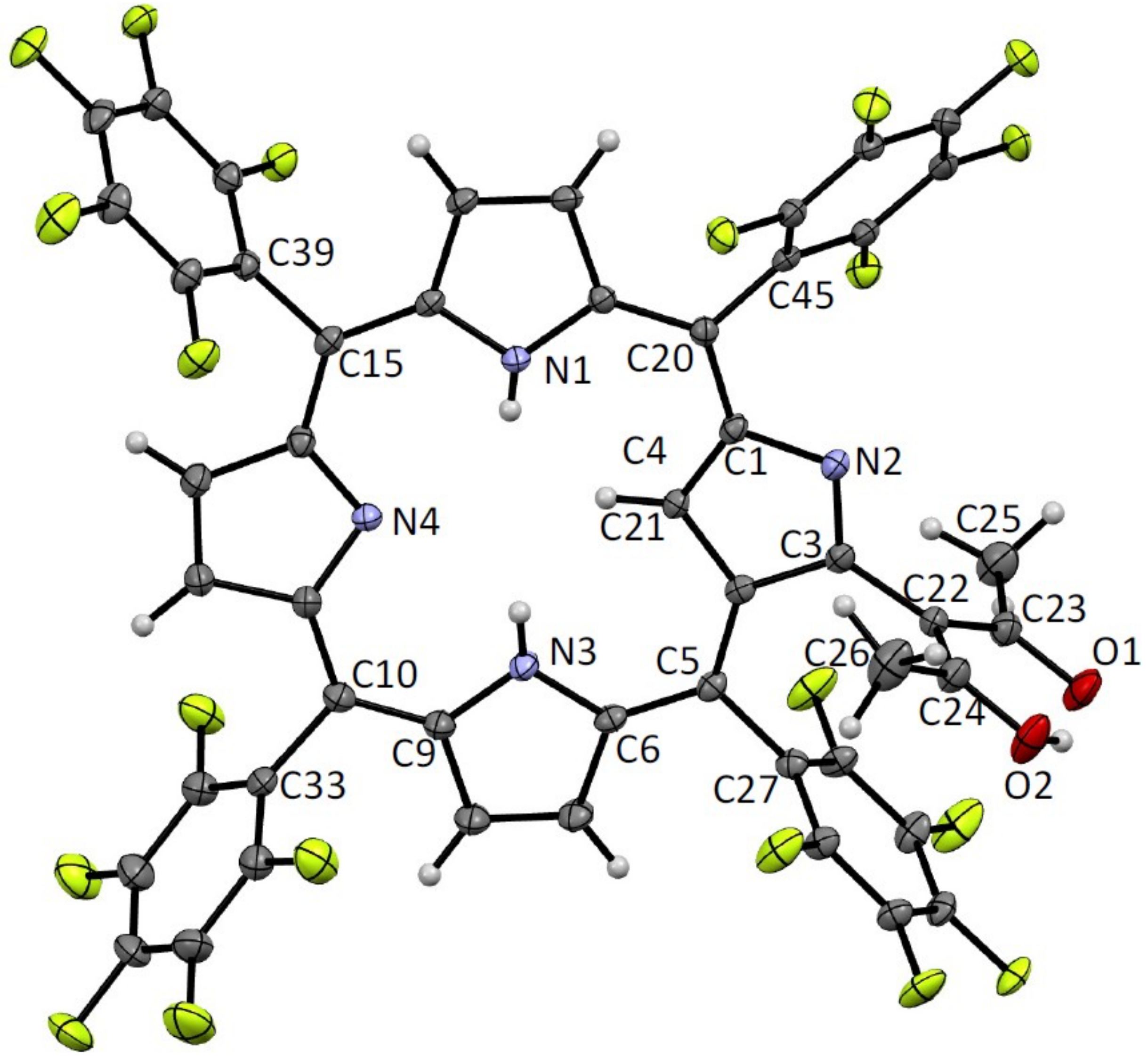

Figure S29. X-ray crystal structures of the compound **2a** top view.

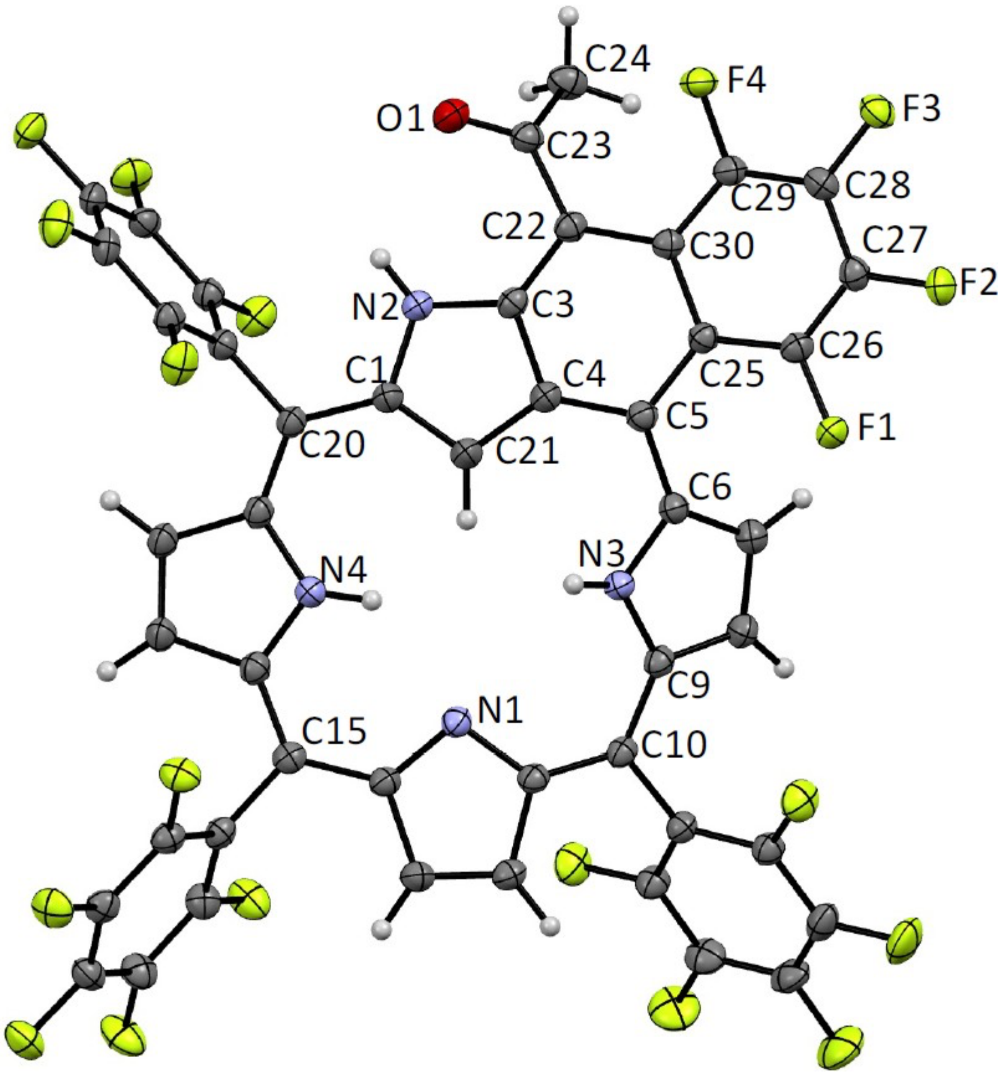

Figure S30. X-ray crystal structures of the compound **5** top view

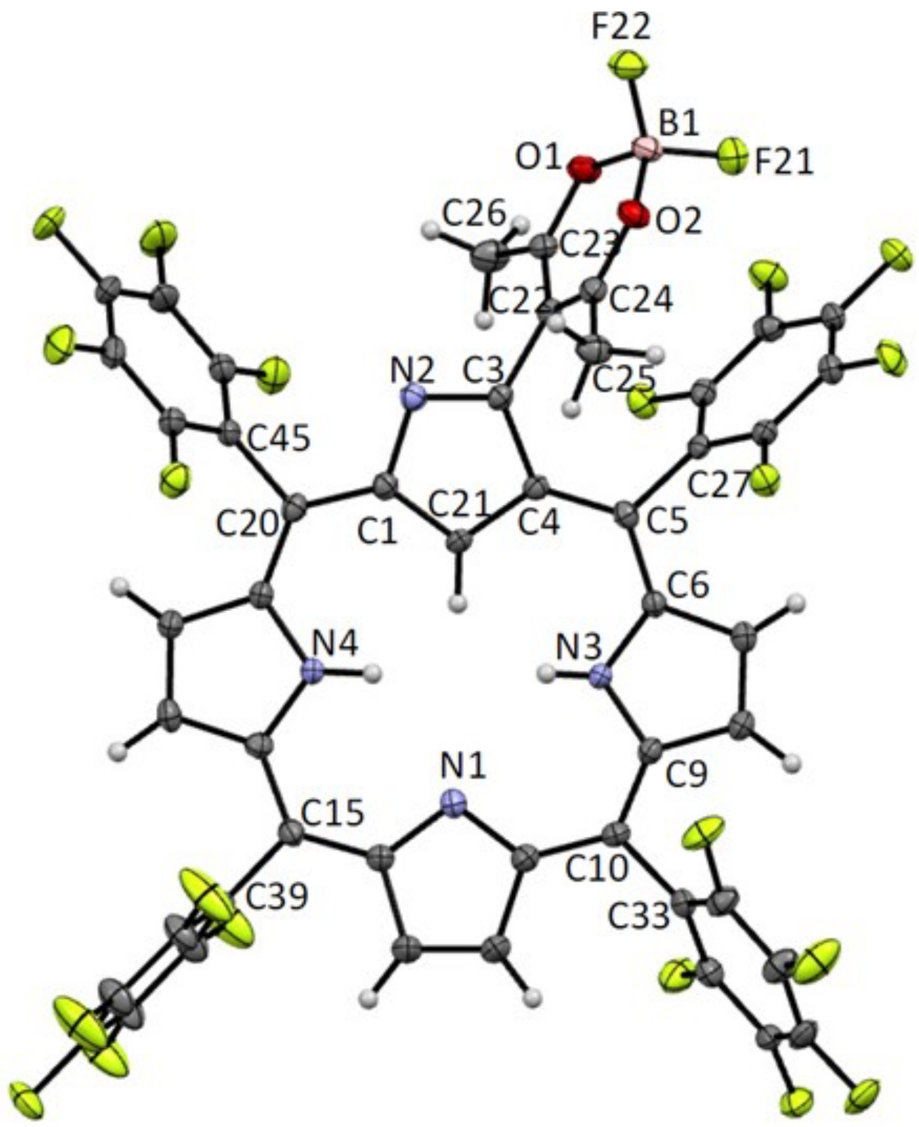

Figure S31. X-ray crystal structures of the compound **4** top view.

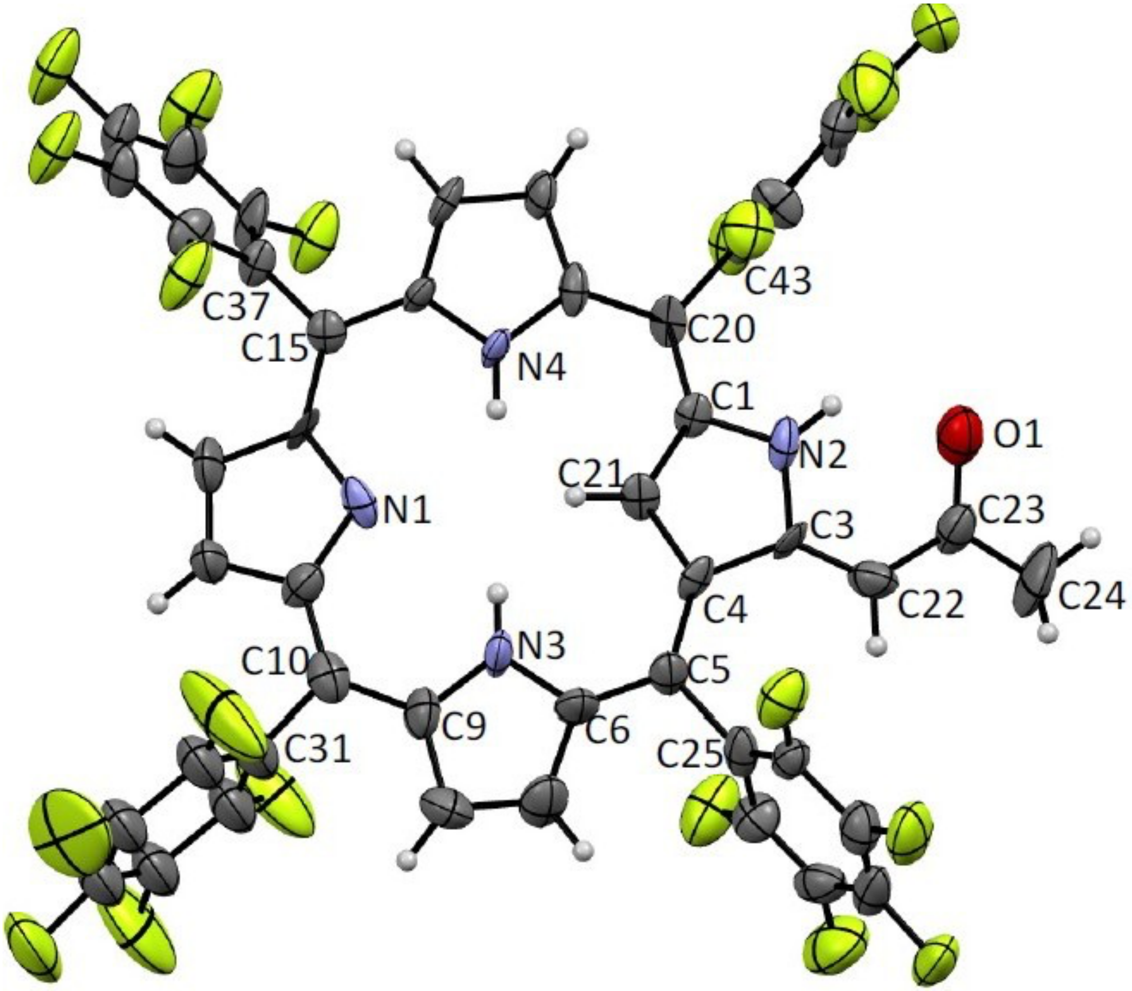

Figure S32. X-ray crystal structures of the compound **3a** top view.

Table S1. Absorption data of compound **1-5**

**Compd**	**Absorption data (λ_{abs}, nm (log ε_{max})**							
	**(I)**	**(II)**	**(III)**	**(IV)**	**(V)**	**(VI)**	**(VII)**	**(VIII)**
**1**	335(4.76)	440(5.29)	471(4.92)	581(4.27)	624(4.2)	679(4.14)	-	-
**2a**	337(4.75)	445(5.3)	475(4.87)	583(4.23)	637(4.00)	687(4.06)	-	-
**2b**	345(4.89)	442(5.32)	501(4.64)	587(4.23)	633(4.30)	698 (4.25)	-	-
**3a**	354(4.56)	399(4.56)	476(5.05)	618(5.45)	682(4.01)	752(5.02)	-	-
**3b**	353(4.88)	467(5.34)	478(5.32)	500(4.91)	610(4.43)	668(4.36)	745(4.04)	
**4**	337(4.60)	445(5.18)	475(4.77)	583(4.06)	641(3.81)	689(4.47)	-	-
**5**	337(4.32)	395(4.30)	488(5.12)	561(3.74)	612(4.00)	635(4.06)	666(3.87)	691(3.95)

Table S2. Crystal data and structure refinement for **i19015 (CCDC: 2322601;** compound **2a)**

Identification code	i19015	
Empirical formula	$C_{50} H_{18} Cl_2 F_{20} N_4 O_2$	
Formula weight	1157.58	
Temperature	100.0(2) K	
Wavelength	1.54178 Å	
Crystal system	Monoclinic	
Space group	C2/c	
Unit cell dimensions	a = 49.2381(9) Å	a= 90°.
	b = 7.46130(10) Å	b= 107.7430(10)°.
	c = 25.6950(4) Å	g = 90°.
Volume	8990.8(3) Å^3	
Z	8	
Density (calculated)	1.709 Mg/m^3	
Absorption coefficient	2.513 mm^{-1}	
F(000)	4600	
Crystal size	0.160 x 0.123 x 0.075 mm^3	
Theta range for data collection	1.884 to 66.593°.	
Index ranges	-57<=h<=57, -8<=k<=8, -30<=l<=29	
Reflections collected	57392	
Independent reflections	7933 [R(int) = 0.1693]	
Completeness to theta = 66.593°	99.8 %	
Absorption correction	Numerical	
Max. and min. transmission	0.8657 and 0.6371	
Refinement method	Full-matrix least-squares on F^2	
Data / restraints / parameters	7933 / 0 / 713	
Goodness-of-fit on F^2	1.025	
Final R indices [I>2sigma(I)]	R1 = 0.0523, wR2 = 0.1339	
R indices (all data)	R1 = 0.1094, wR2 = 0.1436	
Extinction coefficient	n/a	
Largest diff. peak and hole	0.467 and -0.929 e.Å^{-3}	

Table S3. Bond lengths [Å] for **i19015 (**compound **2a)**

N(1)-C(16)	1.362(3)	C(12)-C(13)	1.342(4)	C(30)-C(31)	1.373(4)
N(1)-C(19)	1.375(3)	C(13)-C(14)	1.458(4)	C(31)-F(4)	1.331(3)
N(2)-C(3)	1.314(3)	C(14)-C(15)	1.403(4)	C(31)-C(32)	1.382(4)
N(2)-C(1)	1.420(3)	C(15)-C(16)	1.406(4)	C(32)-F(5)	1.340(3)
N(3)-C(9)	1.354(4)	C(15)-C(39)	1.504(3)	C(33)-C(34)	1.382(4)
N(3)-C(6)	1.380(3)	C(16)-C(17)	1.433(4)	C(33)-C(38)	1.384(4)
N(4)-C(14)	1.373(3)	C(17)-C(18)	1.357(4)	C(34)-F(6)	1.345(4)
N(4)-C(11)	1.374(3)	C(18)-C(19)	1.436(3)	C(34)-C(35)	1.386(4)
C(1)-C(21)	1.390(4)	C(19)-C(20)	1.402(4)	C(35)-F(7)	1.348(4)
C(1)-C(20)	1.403(4)	C(20)-C(45)	1.484(3)	C(35)-C(36)	1.377(5)
C(3)-C(4)	1.476(4)	C(22)-C(24)	1.392(4)	C(36)-F(8)	1.342(3)
C(3)-C(22)	1.480(3)	C(22)-C(23)	1.424(4)	C(36)-C(37)	1.371(5)
C(4)-C(21)	1.410(3)	C(23)-O(1)	1.276(4)	C(37)-F(9)	1.343(4)
C(4)-C(5)	1.415(4)	C(23)-C(25)	1.482(4)	C(37)-C(38)	1.387(4)
C(5)-C(6)	1.394(4)	C(24)-O(2)	1.302(4)	C(38)-F(10)	1.339(3)
C(5)-C(27)	1.498(3)	C(24)-C(26)	1.491(4)	C(39)-C(44)	1.385(4)
C(6)-C(7)	1.433(4)	C(27)-C(32)	1.379(4)	C(39)-C(40)	1.398(4)
C(7)-C(8)	1.353(4)	C(27)-C(28)	1.393(4)	C(40)-F(11)	1.339(3)
C(8)-C(9)	1.432(4)	C(28)-F(1)	1.338(3)	C(40)-C(41)	1.382(4)
C(9)-C(10)	1.410(4)	C(28)-C(29)	1.377(4)	C(41)-F(12)	1.340(3)
C(10)-C(11)	1.401(4)	C(29)-F(2)	1.330(3)	C(41)-C(42)	1.378(4)
C(10)-C(33)	1.500(4)	C(29)-C(30)	1.387(4)	C(42)-F(13)	1.337(3)
C(11)-C(12)	1.464(4)	C(30)-F(3)	1.336(3)	C(42)-C(43)	1.379(4)

C(43)-F(14)	1.338(3)	C(46)-C(47)	1.381(4)	C(49)-C(50)	1.374(4)
C(43)-C(44)	1.390(4)	C(47)-F(19)	1.341(3)	C(50)-F(16)	1.341(3)
C(44)-F(15)	1.339(3)	C(47)-C(48)	1.382(4)	C(51)-Cl(1)	1.750(5)
C(45)-C(50)	1.395(4)	C(48)-F(18)	1.331(3)	C(51)-Cl(2)	1.754(5)
C(45)-C(46)	1.395(4)	C(48)-C(49)	1.388(4)		
C(46)-F(20)	1.335(3)	C(49)-F(17)	1.347(3)		

Table S4. Bond angles [°] for **i19015 (**compound **2a)**

C(16)-N(1)-C(19)	110.0(2)	C(10)-C(9)-C(8)	127.7(3)	C(19)-C(20)-C(1)	124.0(2)
C(3)-N(2)-C(1)	106.7(2)	C(11)-C(10)-C(9)	127.1(3)	C(19)-C(20)-C(45)	118.3(2)
C(9)-N(3)-C(6)	110.1(2)	C(11)-C(10)-C(33)	119.1(2)	C(1)-C(20)-C(45)	117.7(2)
C(14)-N(4)-C(11)	105.8(2)	C(9)-C(10)-C(33)	113.8(2)	C(1)-C(21)-C(4)	107.3(2)
C(21)-C(1)-C(20)	129.5(2)	N(4)-C(11)-C(10)	126.7(2)	C(24)-C(22)-C(23)	118.7(2)
C(21)-C(1)-N(2)	110.4(2)	N(4)-C(11)-C(12)	110.4(2)	C(24)-C(22)-C(3)	120.1(2)
C(20)-C(1)-N(2)	120.1(2)	C(10)-C(11)-C(12)	122.9(2)	C(23)-C(22)-C(3)	121.2(2)
N(2)-C(3)-C(4)	111.1(2)	C(13)-C(12)-C(11)	106.4(2)	O(1)-C(23)-C(22)	121.0(3)
N(2)-C(3)-C(22)	121.7(2)	C(12)-C(13)-C(14)	107.4(2)	O(1)-C(23)-C(25)	117.0(2)
C(4)-C(3)-C(22)	127.0(2)	N(4)-C(14)-C(15)	125.7(2)	C(22)-C(23)-C(25)	121.9(3)
C(21)-C(4)-C(5)	127.0(2)	N(4)-C(14)-C(13)	110.1(2)	O(2)-C(24)-C(22)	121.0(2)
C(21)-C(4)-C(3)	104.5(2)	C(15)-C(14)-C(13)	124.2(2)	O(2)-C(24)-C(26)	115.4(3)
C(5)-C(4)-C(3)	128.3(2)	C(14)-C(15)-C(16)	125.9(2)	C(22)-C(24)-C(26)	123.6(3)
C(6)-C(5)-C(4)	124.0(2)	C(14)-C(15)-C(39)	118.8(2)	C(32)-C(27)-C(28)	116.6(2)
C(6)-C(5)-C(27)	115.5(2)	C(16)-C(15)-C(39)	115.3(2)	C(32)-C(27)-C(5)	120.7(2)
C(4)-C(5)-C(27)	120.5(2)	N(1)-C(16)-C(15)	124.7(2)	C(28)-C(27)-C(5)	122.6(2)
N(3)-C(6)-C(5)	126.3(2)	N(1)-C(16)-C(17)	107.4(2)	F(1)-C(28)-C(29)	117.5(2)
N(3)-C(6)-C(7)	106.3(2)	C(15)-C(16)-C(17)	127.8(2)	F(1)-C(28)-C(27)	120.0(2)
C(5)-C(6)-C(7)	127.3(2)	C(18)-C(17)-C(16)	107.7(2)	C(29)-C(28)-C(27)	122.6(2)
C(8)-C(7)-C(6)	108.3(2)	C(17)-C(18)-C(19)	108.2(2)	F(2)-C(29)-C(28)	120.9(2)
C(7)-C(8)-C(9)	107.8(2)	N(1)-C(19)-C(20)	126.4(2)	F(2)-C(29)-C(30)	120.2(2)
N(3)-C(9)-C(10)	124.9(2)	N(1)-C(19)-C(18)	106.7(2)	C(28)-C(29)-C(30)	119.0(3)
N(3)-C(9)-C(8)	107.4(2)	C(20)-C(19)-C(18)	126.9(2)	F(3)-C(30)-C(31)	119.9(2)

F(3)-C(30)-C(29)	120.2(3)
C(31)-C(30)-C(29)	119.9(2)
F(4)-C(31)-C(30)	120.0(2)
F(4)-C(31)-C(32)	120.1(3)
C(30)-C(31)-C(32)	119.9(2)
F(5)-C(32)-C(27)	119.7(2)
F(5)-C(32)-C(31)	118.3(2)
C(27)-C(32)-C(31)	122.1(3)
C(34)-C(33)-C(38)	116.8(3)
C(34)-C(33)-C(10)	121.0(3)
C(38)-C(33)-C(10)	121.9(3)
F(6)-C(34)-C(33)	119.8(3)
F(6)-C(34)-C(35)	118.2(3)
C(33)-C(34)-C(35)	122.0(3)
F(7)-C(35)-C(36)	120.1(3)
F(7)-C(35)-C(34)	120.3(3)
C(36)-C(35)-C(34)	119.6(3)
F(8)-C(36)-C(37)	120.4(3)
F(8)-C(36)-C(35)	119.5(3)
C(37)-C(36)-C(35)	120.1(3)
F(9)-C(37)-C(36)	120.3(3)
F(9)-C(37)-C(38)	120.4(3)
C(36)-C(37)-C(38)	119.3(3)
F(10)-C(38)-C(33)	120.4(3)
F(10)-C(38)-C(37)	117.3(3)
C(33)-C(38)-C(37)	122.2(3)
C(44)-C(39)-C(40)	116.0(2)
C(44)-C(39)-C(15)	122.2(2)
C(40)-C(39)-C(15)	121.8(2)
F(11)-C(40)-C(41)	117.3(2)
F(11)-C(40)-C(39)	120.1(2)
C(41)-C(40)-C(39)	122.7(2)
F(12)-C(41)-C(42)	119.9(2)
F(12)-C(41)-C(40)	120.5(2)
C(42)-C(41)-C(40)	119.6(3)
F(13)-C(42)-C(41)	120.3(3)
F(13)-C(42)-C(43)	120.1(2)
C(41)-C(42)-C(43)	119.6(2)
F(14)-C(43)-C(42)	120.5(2)
F(14)-C(43)-C(44)	119.6(3)
C(42)-C(43)-C(44)	119.9(2)
F(15)-C(44)-C(39)	120.0(2)
F(15)-C(44)-C(43)	117.7(2)
C(39)-C(44)-C(43)	122.3(3)
C(50)-C(45)-C(46)	115.9(2)
C(50)-C(45)-C(20)	123.5(2)
C(46)-C(45)-C(20)	120.6(2)
F(20)-C(46)-C(47)	117.5(2)
F(20)-C(46)-C(45)	120.3(2)
C(47)-C(46)-C(45)	122.3(2)
F(19)-C(47)-C(46)	120.3(2)
F(19)-C(47)-C(48)	119.4(3)
C(46)-C(47)-C(48)	120.3(2)
F(18)-C(48)-C(47)	120.8(2)
F(18)-C(48)-C(49)	120.5(3)
C(47)-C(48)-C(49)	118.7(3)
F(17)-C(49)-C(50)	119.9(2)
F(17)-C(49)-C(48)	119.9(2)
C(50)-C(49)-C(48)	120.2(2)
F(16)-C(50)-C(49)	117.0(2)
F(16)-C(50)-C(45)	120.3(2)
C(49)-C(50)-C(45)	122.6(2)
Cl(1)-C(51)-Cl(2)	112.2(2)

Table S5. Crystal data and structure refinement for **i18568 (CCDC: 2322785;** compound **5).**

Identification code	i18568_sq	
Empirical formula	$C_{47} H_{13} F_{19} N_4 O$	
Formula weight	1010.61	
Temperature	100.0(2) K	
Wavelength	0.71073 Å	
Crystal system	Triclinic	
Space group	P-1	
Unit cell dimensions	a = 12.2616(3) Å	a= 102.7850(10)°.
	b = 14.1706(3) Å	b= 97.4290(10)°.
	c = 14.8201(4) Å	g = 114.1760(10)°.
Volume	2219.86(10) $Å^3$	
Z	2	
Density (calculated)	1.512 Mg/m^3	
Absorption coefficient	0.148 mm^{-1}	
F(000)	1004	
Crystal size	0.204 x 0.179 x 0.072 mm^3	
Theta range for data collection	2.381 to 30.157°.	
Index ranges	-17<=h<=17, -19<=k<=20, -20<=l<=20	
Reflections collected	119095	
Independent reflections	13071 [R(int) = 0.0867]	
Completeness to theta = 25.242°	99.8 %	
Absorption correction	Numerical	
Max. and min. transmission	1 and 0.9196	
Refinement method	Full-matrix least-squares on F^2	
Data / restraints / parameters	13071 / 0 / 654	
Goodness-of-fit on F^2	1.013	
Final R indices [I>2sigma(I)]	R1 = 0.0598, wR2 = 0.1737	
R indices (all data)	R1 = 0.0831, wR2 = 0.1953	
Extinction coefficient	0.0142(16)	
Largest diff. peak and hole	0.539 and -0.424 e.$Å^{-3}$	

Table S6. Bond lengths [Å] for **i18568_(compound 5)**

N(1)-C(14)	1.370(3)	C(12)-C(13)	1.353(3)	C(29)-C(30)	1.399(3)
N(1)-C(11)	1.383(3)	C(13)-C(14)	1.450(3)	C(31)-C(32)	1.390(3)
N(2)-C(3)	1.374(3)	C(14)-C(15)	1.420(3)	C(31)-C(36)	1.397(3)
N(2)-C(1)	1.388(3)	C(15)-C(16)	1.383(3)	C(32)-F(5)	1.334(3)
N(3)-C(9)	1.367(3)	C(15)-C(37)	1.493(3)	C(32)-C(33)	1.391(3)
N(3)-C(6)	1.372(3)	C(16)-C(17)	1.436(3)	C(33)-F(6)	1.334(3)
N(4)-C(19)	1.372(3)	C(17)-C(18)	1.361(3)	C(33)-C(34)	1.372(4)
N(4)-C(16)	1.371(3)	C(18)-C(19)	1.446(3)	C(34)-F(7)	1.338(3)
C(1)-C(21)	1.390(3)	C(19)-C(20)	1.385(3)	C(34)-C(35)	1.378(4)
C(1)-C(20)	1.424(3)	C(20)-C(43)	1.486(3)	C(35)-F(8)	1.337(3)
C(3)-C(22)	1.377(3)	C(22)-C(30)	1.440(3)	C(35)-C(36)	1.385(3)
C(3)-C(4)	1.433(3)	C(22)-C(23)	1.492(3)	C(36)-F(9)	1.333(2)
C(4)-C(21)	1.404(3)	C(23)-O(1)	1.223(3)	C(37)-C(42)	1.390(3)
C(4)-C(5)	1.412(3)	C(23)-C(24)	1.508(3)	C(37)-C(38)	1.393(3)
C(5)-C(6)	1.439(3)	C(25)-C(26)	1.416(3)	C(38)-F(10)	1.341(2)
C(5)-C(25)	1.442(3)	C(25)-C(30)	1.447(3)	C(38)-C(39)	1.381(3)
C(6)-C(7)	1.409(3)	C(26)-F(1)	1.347(2)	C(39)-F(11)	1.341(3)
C(7)-C(8)	1.383(3)	C(26)-C(27)	1.371(3)	C(39)-C(40)	1.372(3)
C(8)-C(9)	1.415(3)	C(27)-F(2)	1.341(2)	C(40)-F(12)	1.337(2)
C(9)-C(10)	1.408(3)	C(27)-C(28)	1.388(3)	C(40)-C(41)	1.386(3)
C(10)-C(11)	1.404(3)	C(28)-F(3)	1.337(2)	C(41)-F(13)	1.334(2)
C(10)-C(31)	1.490(3)	C(28)-C(29)	1.370(3)	C(41)-C(42)	1.379(3)
C(11)-C(12)	1.451(3)	C(29)-F(4)	1.346(2)	C(42)-F(14)	1.336(2)

C(43)-C(44)	1.394(3)	C(45)-F(16)	1.336(2)	C(47)-F(18)	1.348(2)
C(43)-C(48)	1.392(3)	C(45)-C(46)	1.382(3)	C(47)-C(48)	1.383(3)
C(44)-F(15)	1.332(2)	C(46)-F(17)	1.336(2)	C(48)-F(19)	1.339(2)
C(44)-C(45)	1.381(3)	C(46)-C(47)	1.370(3)		

Table S7. Bond angles [°] for compound **i18568 (**compound **5)**

C(14)-N(1)-C(11)	104.69(17)	C(8)-C(9)-C(10)	128.76(19)	C(19)-C(20)-C(1)	124.13(18)
C(3)-N(2)-C(1)	109.63(17)	C(9)-C(10)-C(11)	127.50(18)	C(19)-C(20)-C(43)	118.47(18)
C(9)-N(3)-C(6)	110.48(18)	C(9)-C(10)-C(31)	116.46(18)	C(1)-C(20)-C(43)	117.33(18)
C(19)-N(4)-C(16)	111.25(17)	C(11)-C(10)-C(31)	116.00(18)	C(4)-C(21)-C(1)	108.88(19)
N(2)-C(1)-C(21)	107.57(18)	N(1)-C(11)-C(10)	127.29(19)	C(3)-C(22)-C(30)	116.13(18)
N(2)-C(1)-C(20)	122.08(18)	N(1)-C(11)-C(12)	110.66(18)	C(3)-C(22)-C(23)	116.71(18)
C(21)-C(1)-C(20)	130.25(19)	C(10)-C(11)-C(12)	121.90(18)	C(30)-C(22)-C(23)	126.90(18)
N(2)-C(3)-C(22)	128.34(18)	C(13)-C(12)-C(11)	106.79(18)	O(1)-C(23)-C(22)	119.0(2)
N(2)-C(3)-C(4)	107.57(17)	C(12)-C(13)-C(14)	106.31(19)	O(1)-C(23)-C(24)	120.0(2)
C(22)-C(3)-C(4)	123.58(18)	N(1)-C(14)-C(15)	125.87(18)	C(22)-C(23)-C(24)	120.28(19)
C(21)-C(4)-C(5)	132.92(19)	N(1)-C(14)-C(13)	111.38(18)	C(26)-C(25)-C(5)	122.83(18)
C(21)-C(4)-C(3)	106.27(18)	C(15)-C(14)-C(13)	122.63(19)	C(26)-C(25)-C(30)	116.62(18)
C(5)-C(4)-C(3)	120.52(18)	C(16)-C(15)-C(14)	124.53(19)	C(5)-C(25)-C(30)	120.33(18)
C(4)-C(5)-C(6)	119.24(18)	C(16)-C(15)-C(37)	117.17(18)	F(1)-C(26)-C(27)	116.13(18)
C(4)-C(5)-C(25)	116.52(18)	C(14)-C(15)-C(37)	118.28(18)	F(1)-C(26)-C(25)	120.64(18)
C(6)-C(5)-C(25)	123.94(18)	N(4)-C(16)-C(15)	123.33(19)	C(27)-C(26)-C(25)	123.05(19)
N(3)-C(6)-C(7)	106.93(18)	N(4)-C(16)-C(17)	106.38(18)	F(2)-C(27)-C(26)	120.98(19)
N(3)-C(6)-C(5)	122.20(18)	C(15)-C(16)-C(17)	130.3(2)	F(2)-C(27)-C(28)	119.16(18)
C(7)-C(6)-C(5)	129.9(2)	C(18)-C(17)-C(16)	108.26(19)	C(26)-C(27)-C(28)	119.76(19)
C(8)-C(7)-C(6)	107.91(19)	C(17)-C(18)-C(19)	108.23(19)	F(3)-C(28)-C(27)	119.70(19)
C(7)-C(8)-C(9)	107.85(19)	N(4)-C(19)-C(20)	127.20(19)	F(3)-C(28)-C(29)	121.04(19)
N(3)-C(9)-C(8)	106.78(18)	N(4)-C(19)-C(18)	105.87(18)	C(27)-C(28)-C(29)	119.15(19)
N(3)-C(9)-C(10)	124.46(19)	C(20)-C(19)-C(18)	126.87(19)	F(4)-C(29)-C(30)	119.81(18)

F(4)-C(29)-C(28)	116.61(18)	F(9)-C(36)-C(35)	117.68(19)	C(37)-C(42)-C(41)	122.50(19)
C(30)-C(29)-C(28)	123.37(19)	F(9)-C(36)-C(31)	120.33(18)	C(44)-C(43)-C(48)	116.41(18)
C(29)-C(30)-C(22)	121.00(18)	C(35)-C(36)-C(31)	122.0(2)	C(44)-C(43)-C(20)	122.29(18)
C(29)-C(30)-C(25)	117.89(18)	C(42)-C(37)-C(38)	116.29(19)	C(48)-C(43)-C(20)	121.29(18)
C(22)-C(30)-C(25)	120.77(18)	C(42)-C(37)-C(15)	121.63(18)	F(15)-C(44)-C(45)	117.82(19)
C(32)-C(31)-C(36)	116.15(19)	C(38)-C(37)-C(15)	122.06(18)	F(15)-C(44)-C(43)	120.12(18)
C(32)-C(31)-C(10)	122.69(19)	F(10)-C(38)-C(39)	118.20(19)	C(45)-C(44)-C(43)	122.06(19)
C(36)-C(31)-C(10)	121.15(18)	F(10)-C(38)-C(37)	119.67(19)	F(16)-C(45)-C(44)	120.3(2)
F(5)-C(32)-C(31)	119.66(19)	C(39)-C(38)-C(37)	122.1(2)	F(16)-C(45)-C(46)	119.82(19)
F(5)-C(32)-C(33)	117.84(19)	F(11)-C(39)-C(40)	119.3(2)	C(44)-C(45)-C(46)	119.9(2)
C(31)-C(32)-C(33)	122.4(2)	F(11)-C(39)-C(38)	120.8(2)	F(17)-C(46)-C(47)	120.4(2)
F(6)-C(33)-C(34)	120.2(2)	C(40)-C(39)-C(38)	119.9(2)	F(17)-C(46)-C(45)	120.0(2)
F(6)-C(33)-C(32)	120.2(2)	F(12)-C(40)-C(39)	120.5(2)	C(47)-C(46)-C(45)	119.52(19)
C(34)-C(33)-C(32)	119.6(2)	F(12)-C(40)-C(41)	119.8(2)	F(18)-C(47)-C(46)	119.70(19)
F(7)-C(34)-C(33)	120.5(2)	C(39)-C(40)-C(41)	119.8(2)	F(18)-C(47)-C(48)	120.08(19)
F(7)-C(34)-C(35)	119.7(2)	F(13)-C(41)-C(40)	120.02(19)	C(46)-C(47)-C(48)	120.20(19)
C(33)-C(34)-C(35)	119.9(2)	F(13)-C(41)-C(42)	120.58(19)	F(19)-C(48)-C(43)	119.83(18)
F(8)-C(35)-C(34)	119.9(2)	C(40)-C(41)-C(42)	119.4(2)	F(19)-C(48)-C(47)	118.23(18)
F(8)-C(35)-C(36)	120.1(2)	F(14)-C(42)-C(37)	119.74(19)	C(43)-C(48)-C(47)	121.94(19)
C(34)-C(35)-C(36)	120.0(2)	F(14)-C(42)-C(41)	117.76(19)		

Table S8. Crystal data and structure refinement for **i19253 (CCDC: 2322592;** compound **4)**

Identification code	i19253	
Empirical formula	$C_{50} H_{16} B Cl_3 F_{22} N_4 O_2$	
Formula weight	1239.83	
Temperature	100.0(2) K	
Wavelength	0.71073 Å	
Crystal system	Monoclinic	
Space group	$P2_1/c$	
Unit cell dimensions	a = 26.8619(19) Å	a= 90°.
	b = 7.4507(4) Å	b= 112.226(2)°.
	c = 25.7190(18) Å	g = 90°.
Volume	4764.9(5) $Å^3$	
Z	4	
Density (calculated)	1.728 Mg/m^3	
Absorption coefficient	0.328 mm^{-1}	
F(000)	2456	
Crystal size	0.364 x 0.136 x 0.135 mm^3	
Theta range for data collection	1.638 to 27.099°.	
Index ranges	-34<=h<=34, -9<=k<=9, -32<=l<=32	
Reflections collected	80460	
Independent reflections	10520 [R(int) = 0.1206]	
Completeness to theta = 25.242°	99.9 %	
Absorption correction	Numerical	
Max. and min. transmission	0.8678 and 0.7138	
Refinement method	Full-matrix least-squares on F^2	
Data / restraints / parameters	10520 / 0 / 749	
Goodness-of-fit on F^2	1.022	
Final R indices [I>2sigma(I)]	R1 = 0.0542, wR2 = 0.1224	
R indices (all data)	R1 = 0.0872, wR2 = 0.1443	
Extinction coefficient	n/a	
Largest diff. peak and hole	0.483 and -0.644 e.$Å^{-3}$	

Table S9. Bond lengths [Å] for **i19253 (**compound **4).**

C(1)-C(21)	1.395(4)	C(11)-C(12)	1.456(4)	C(27)-C(32)	1.387(4)
C(1)-C(20)	1.408(4)	C(12)-C(13)	1.332(4)	C(28)-F(1)	1.345(3)
C(1)-N(2)	1.412(3)	C(13)-C(14)	1.456(4)	C(28)-C(29)	1.382(4)
N(1)-C(14)	1.371(3)	C(14)-C(15)	1.393(4)	C(29)-F(2)	1.339(3)
N(1)-C(11)	1.373(4)	C(15)-C(16)	1.399(4)	C(29)-C(30)	1.370(4)
N(2)-C(3)	1.309(3)	C(15)-C(39)	1.503(4)	C(30)-F(3)	1.338(3)
N(3)-C(9)	1.364(3)	C(16)-C(17)	1.418(4)	C(30)-C(31)	1.377(4)
N(3)-C(6)	1.377(3)	C(17)-C(18)	1.362(4)	C(31)-F(4)	1.333(3)
N(4)-C(16)	1.368(3)	C(18)-C(19)	1.425(4)	C(31)-C(32)	1.381(4)
N(4)-C(19)	1.379(3)	C(19)-C(20)	1.396(4)	C(32)-F(5)	1.343(3)
C(3)-C(4)	1.477(4)	C(20)-C(45)	1.490(4)	C(33)-C(34)	1.382(4)
C(3)-C(22)	1.482(4)	C(22)-C(24)	1.391(4)	C(33)-C(38)	1.384(4)
C(4)-C(21)	1.405(4)	C(22)-C(23)	1.396(4)	C(34)-F(6)	1.346(3)
C(4)-C(5)	1.408(4)	C(23)-O(1)	1.304(3)	C(34)-C(35)	1.371(4)
C(5)-C(6)	1.401(4)	C(23)-C(26)	1.482(4)	C(35)-F(7)	1.343(4)
C(5)-C(27)	1.499(4)	O(1)-B(1)	1.487(4)	C(35)-C(36)	1.371(4)
C(6)-C(7)	1.429(4)	C(24)-O(2)	1.298(3)	C(36)-F(8)	1.340(3)
C(7)-C(8)	1.355(4)	C(24)-C(25)	1.487(4)	C(36)-C(37)	1.371(4)
C(8)-C(9)	1.425(4)	O(2)-B(1)	1.489(4)	C(37)-F(9)	1.346(3)
C(9)-C(10)	1.395(4)	B(1)-F(21)	1.363(4)	C(37)-C(38)	1.382(4)
C(10)-C(11)	1.404(4)	B(1)-F(22)	1.365(4)	C(38)-F(10)	1.339(3)
C(10)-C(33)	1.495(4)	C(27)-C(28)	1.382(4)	C(39)-C(40)	1.373(4)

C(39)-C(44)	1.378(4)	C(44)-F(15)	1.344(3)	C(49)-F(19)	1.339(3)
C(40)-F(11)	1.346(3)	C(45)-C(50)	1.384(4)	C(49)-C(50)	1.377(4)
C(40)-C(41)	1.375(4)	C(45)-C(46)	1.391(4)	C(50)-F(20)	1.343(3)
C(41)-F(12)	1.340(3)	C(46)-F(16)	1.345(3)	C(51)-Cl(1)	1.753(3)
C(41)-C(42)	1.370(4)	C(46)-C(47)	1.368(4)	C(51)-Cl(2)	1.753(3)
C(42)-F(13)	1.341(3)	C(47)-F(17)	1.349(3)	C(51)-Cl(3)	1.764(3)
C(42)-C(43)	1.369(4)	C(47)-C(48)	1.374(5)		
C(43)-F(14)	1.333(3)	C(48)-F(18)	1.336(3)		
C(43)-C(44)	1.375(4)	C(48)-C(49)	1.376(4)		

Table S10. Bond angles [°] of **i19253 (**compound **4)**

C(21)-C(1)-C(20)	130.1(2)	C(10)-C(9)-C(8)	128.7(3)	C(19)-C(20)-C(1)	124.4(2)
C(21)-C(1)-N(2)	110.4(2)	C(9)-C(10)-C(11)	126.6(3)	C(19)-C(20)-C(45)	117.8(2)
C(20)-C(1)-N(2)	119.3(2)	C(9)-C(10)-C(33)	115.6(2)	C(1)-C(20)-C(45)	117.7(2)
C(14)-N(1)-C(11)	104.8(2)	C(11)-C(10)-C(33)	117.7(2)	C(1)-C(21)-C(4)	107.5(2)
C(3)-N(2)-C(1)	106.4(2)	N(1)-C(11)-C(10)	126.5(2)	C(24)-C(22)-C(23)	118.6(2)
C(9)-N(3)-C(6)	110.7(2)	N(1)-C(11)-C(12)	110.7(2)	C(24)-C(22)-C(3)	121.7(2)
C(16)-N(4)-C(19)	110.3(2)	C(10)-C(11)-C(12)	122.7(3)	C(23)-C(22)-C(3)	119.2(2)
N(2)-C(3)-C(4)	111.8(2)	C(13)-C(12)-C(11)	106.8(3)	O(1)-C(23)-C(22)	121.2(3)
N(2)-C(3)-C(22)	119.2(2)	C(12)-C(13)-C(14)	106.8(3)	O(1)-C(23)-C(26)	114.6(2)
C(4)-C(3)-C(22)	129.0(2)	N(1)-C(14)-C(15)	126.3(2)	C(22)-C(23)-C(26)	124.2(3)
C(21)-C(4)-C(5)	128.0(2)	N(1)-C(14)-C(13)	110.8(2)	C(23)-O(1)-B(1)	123.2(2)
C(21)-C(4)-C(3)	103.9(2)	C(15)-C(14)-C(13)	122.9(3)	O(2)-C(24)-C(22)	121.4(2)
C(5)-C(4)-C(3)	128.0(2)	C(14)-C(15)-C(16)	126.8(3)	O(2)-C(24)-C(25)	115.0(2)
C(6)-C(5)-C(4)	124.1(2)	C(14)-C(15)-C(39)	118.0(2)	C(22)-C(24)-C(25)	123.6(2)
C(6)-C(5)-C(27)	115.7(2)	C(16)-C(15)-C(39)	115.2(2)	C(24)-O(2)-B(1)	123.2(2)
C(4)-C(5)-C(27)	120.1(2)	N(4)-C(16)-C(15)	123.8(3)	F(21)-B(1)-F(22)	112.5(2)
N(3)-C(6)-C(5)	126.0(2)	N(4)-C(16)-C(17)	107.0(2)	F(21)-B(1)-O(1)	108.8(2)
N(3)-C(6)-C(7)	105.9(2)	C(15)-C(16)-C(17)	129.1(3)	F(22)-B(1)-O(1)	108.0(3)
C(5)-C(6)-C(7)	127.9(2)	C(18)-C(17)-C(16)	108.0(2)	F(21)-B(1)-O(2)	109.1(3)
C(8)-C(7)-C(6)	108.3(2)	C(17)-C(18)-C(19)	108.6(2)	F(22)-B(1)-O(2)	108.8(2)
C(7)-C(8)-C(9)	108.4(2)	N(4)-C(19)-C(20)	125.4(2)	O(1)-B(1)-O(2)	109.6(2)
N(3)-C(9)-C(10)	124.8(2)	N(4)-C(19)-C(18)	106.0(2)	C(28)-C(27)-C(32)	115.8(2)
N(3)-C(9)-C(8)	106.5(2)	C(20)-C(19)-C(18)	128.6(2)	C(28)-C(27)-C(5)	120.6(2)

C(32)-C(27)-C(5)	123.6(2)
F(1)-C(28)-C(27)	119.8(2)
F(1)-C(28)-C(29)	117.5(2)
C(27)-C(28)-C(29)	122.7(3)
F(2)-C(29)-C(30)	120.5(2)
F(2)-C(29)-C(28)	119.8(3)
C(30)-C(29)-C(28)	119.7(2)
F(3)-C(30)-C(29)	120.2(2)
F(3)-C(30)-C(31)	120.1(3)
C(29)-C(30)-C(31)	119.7(2)
F(4)-C(31)-C(30)	120.6(2)
F(4)-C(31)-C(32)	120.0(2)
C(30)-C(31)-C(32)	119.5(3)
F(5)-C(32)-C(31)	116.7(2)
F(5)-C(32)-C(27)	120.6(2)
C(31)-C(32)-C(27)	122.6(2)
C(34)-C(33)-C(38)	116.6(3)
C(34)-C(33)-C(10)	120.3(3)
C(38)-C(33)-C(10)	123.1(3)
F(6)-C(34)-C(35)	118.4(3)
F(6)-C(34)-C(33)	119.1(3)
C(35)-C(34)-C(33)	122.5(3)
F(7)-C(35)-C(36)	120.1(3)
F(7)-C(35)-C(34)	120.5(3)
C(36)-C(35)-C(34)	119.4(3)
F(8)-C(36)-C(37)	120.0(3)
F(8)-C(36)-C(35)	119.8(3)
C(37)-C(36)-C(35)	120.1(3)
F(9)-C(37)-C(36)	119.0(3)
F(9)-C(37)-C(38)	121.4(3)
C(36)-C(37)-C(38)	119.6(3)
F(10)-C(38)-C(37)	118.7(3)
F(10)-C(38)-C(33)	119.6(3)
C(37)-C(38)-C(33)	121.7(3)
C(40)-C(39)-C(44)	115.8(3)
C(40)-C(39)-C(15)	122.5(2)
C(44)-C(39)-C(15)	121.7(3)
F(11)-C(40)-C(39)	119.7(3)
F(11)-C(40)-C(41)	117.5(3)
C(39)-C(40)-C(41)	122.8(3)
F(12)-C(41)-C(42)	119.7(3)
F(12)-C(41)-C(40)	120.9(3)
C(42)-C(41)-C(40)	119.4(3)
F(13)-C(42)-C(43)	119.5(3)
F(13)-C(42)-C(41)	120.6(3)
C(43)-C(42)-C(41)	119.9(3)
F(14)-C(43)-C(42)	119.9(3)
F(14)-C(43)-C(44)	121.0(3)
C(42)-C(43)-C(44)	119.1(3)
F(15)-C(44)-C(43)	118.0(3)
F(15)-C(44)-C(39)	118.9(3)
C(43)-C(44)-C(39)	123.0(3)
C(50)-C(45)-C(46)	116.0(3)
C(50)-C(45)-C(20)	122.0(2)
C(46)-C(45)-C(20)	122.0(2)
F(16)-C(46)-C(47)	118.0(3)
F(16)-C(46)-C(45)	119.9(3)
C(47)-C(46)-C(45)	122.1(3)
F(17)-C(47)-C(46)	120.0(3)
F(17)-C(47)-C(48)	119.6(3)
C(46)-C(47)-C(48)	120.3(3)
F(18)-C(48)-C(47)	120.6(3)
F(18)-C(48)-C(49)	120.1(3)
C(47)-C(48)-C(49)	119.3(3)
F(19)-C(49)-C(48)	120.1(3)
F(19)-C(49)-C(50)	120.3(3)
C(48)-C(49)-C(50)	119.5(3)
F(20)-C(50)-C(49)	117.1(3)
F(20)-C(50)-C(45)	120.2(2)
C(49)-C(50)-C(45)	122.7(3)
Cl(1)-C(51)-Cl(2)	112.02(17)
Cl(1)-C(51)-Cl(3)	110.98(17)
Cl(2)-C(51)-Cl(3)	109.57(18)

Table S11. Crystal data and structure refinement for **d24266**_sq. **(**compound **3a)**

Identification code	d24266_sq	
Empirical formula	C47 H14 F20 N4 O	
Formula weight	1030.62	
Temperature	200(2) K	
Wavelength	0.71073 Å	
Crystal system	Monoclinic	
Space group	$P2_1/c$	
Unit cell dimensions	a = 18.169(4) Å	a= 90°.
	b = 11.781(2) Å	b= 103.38(3)°.
	c = 23.950(5) Å	g = 90°.
Volume	4987.4(18) $Å^3$	
Z	4	
Density (calculated)	1.373 Mg/m^3	
Absorption coefficient	0.136 mm^{-1}	
F(000)	2048	
Crystal size	0.28 x 0.16 x 0.06 mm^3	
Theta range for data collection	1.937 to 26.120°.	
Index ranges	-20<=h<=21, -14<=k<=14, -29<=l<=27	
Reflections collected	44622	
Independent reflections	9207 [R(int) = 0.1983]	
Completeness to theta = 25.242°	99.4 %	
Absorption correction	Semi-empirical from equivalents	
Max. and min. transmission	0.9922 and 0.9645	
Refinement method	Full-matrix least-squares on F^2	
Data / restraints / parameters	9207 / 0 / 608	
Goodness-of-fit on F^2	1.187	
Final R indices [I>2sigma(I)]	R1 = 0.1809, wR2 = 0.4793	
R indices (all data)	R1 = 0.3123, wR2 = 0.5603	
Extinction coefficient	n/a	
Largest diff. peak and hole	0.533 and -0.422 e.$Å^{-3}$	

Table S12. Bond lengths [Å] and angles [°] for **d24266**_sq. **(**compound **3a)**

C(31)-C(32)	1.3900	C(16)-C(17)	1.352(15)	C(12)-C(11)	1.382(17)
C(31)-C(36)	1.3900	C(16)-C(15)	1.401(14)	C(43)-C(48)	1.326(16)
C(31)-C(10)	1.446(15)	C(20)-C(19)	1.399(14)	C(43)-C(44)	1.349(15)
C(32)-F(6)	1.301(17)	C(20)-C(43)	1.499(15)	C(44)-F(20)	1.340(14)
C(32)-C(33)	1.3900	C(6)-C(5)	1.358(16)	C(44)-C(45)	1.361(19)
C(33)-F(7)	1.21(2)	C(6)-C(7)	1.423(18)	C(37)-C(42)	1.315(16)
C(33)-C(34)	1.3900	C(15)-C(37)	1.400(14)	C(37)-C(38)	1.393(16)
C(34)-F(8)	1.336(12)	C(15)-C(14)	1.400(15)	C(25)-C(26)	1.323(18)
C(34)-C(35)	1.3900	C(14)-C(13)	1.421(16)	C(25)-C(30)	1.37(2)
C(35)-F(9)	1.357(18)	C(17)-C(18)	1.328(14)	C(48)-F(16)	1.305(12)
C(35)-C(36)	1.3900	C(19)-C(18)	1.408(16)	C(48)-C(47)	1.409(19)
C(36)-F(10)	1.202(15)	C(9)-C(8)	1.418(19)	C(45)-F(19)	1.272(15)
N(4)-C(16)	1.317(11)	C(9)-C(10)	1.421(18)	C(45)-C(46)	1.40(2)
N(4)-C(19)	1.355(14)	C(23)-O(1)	1.301(17)	C(46)-F(18)	1.315(16)
N(3)-C(6)	1.340(14)	C(23)-C(24)	1.397(18)	C(46)-C(47)	1.38(2)
N(3)-C(9)	1.341(15)	C(23)-C(22)	1.398(18)	C(47)-F(17)	1.340(16)
N(1)-C(14)	1.321(13)	C(4)-C(5)	1.391(16)	C(40)-C(39)	1.285(19)
N(1)-C(11)	1.399(16)	C(4)-C(3)	1.449(15)	C(40)-F(13)	1.337(14)
N(2)-C(1)	1.333(12)	C(3)-C(22)	1.315(15)	C(40)-C(41)	1.353(17)
N(2)-C(3)	1.358(14)	C(8)-C(7)	1.36(2)	C(41)-F(12)	1.265(13)
C(1)-C(20)	1.358(14)	C(5)-C(25)	1.471(17)	C(41)-C(42)	1.367(17)
C(1)-C(21)	1.378(14)	C(10)-C(11)	1.359(18)	C(26)-F(1)	1.294(15)
C(21)-C(4)	1.375(14)	C(13)-C(12)	1.347(18)	C(26)-C(27)	1.385(19)

C(28)-C(29)	1.36(2)	C(38)-C(39)	1.338(18)	C(30)-F(5)	1.295(16)
C(28)-F(3)	1.324(15)	C(39)-F(14)	1.388(16)	C(42)-F(11)	1.332(14)
C(28)-C(27)	1.34(2)	C(29)-F(4)	1.313(18)	C(27)-F(2)	1.306(17)
C(38)-F(15)	1.253(13)	C(29)-C(30)	1.29(2)		

Table S13. Bond angels [°] of **d24266**_sq. **(**compound **3a)**

C(32)-C(31)-C(36)	120.0	N(2)-C(1)-C(21)	106.9(10)	N(3)-C(9)-C(10)	120.6(12)
C(32)-C(31)-C(10)	118.6(10)	C(20)-C(1)-C(21)	132.3(9)	C(8)-C(9)-C(10)	127.0(13)
C(36)-C(31)-C(10)	121.1(10)	C(1)-C(21)-C(4)	111.5(10)	O(1)-C(23)-C(24)	118.5(14)
F(6)-C(32)-C(33)	119.3(10)	N(4)-C(16)-C(17)	108.6(9)	O(1)-C(23)-C(22)	122.3(11)
F(6)-C(32)-C(31)	120.6(10)	N(4)-C(16)-C(15)	124.1(10)	C(24)-C(23)-C(22)	119.2(16)
C(33)-C(32)-C(31)	120.0	C(17)-C(16)-C(15)	127.3(9)	C(21)-C(4)-C(5)	128.7(10)
F(7)-C(33)-C(32)	122.5(16)	C(1)-C(20)-C(19)	124.1(10)	C(21)-C(4)-C(3)	103.0(10)
F(7)-C(33)-C(34)	117.3(17)	C(1)-C(20)-C(43)	123.3(9)	C(5)-C(4)-C(3)	128.3(9)
C(32)-C(33)-C(34)	120.0	C(19)-C(20)-C(43)	112.5(10)	C(22)-C(3)-N(2)	117.0(10)
F(8)-C(34)-C(33)	124.7(12)	N(3)-C(6)-C(5)	126.2(11)	C(22)-C(3)-C(4)	134.8(11)
F(8)-C(34)-C(35)	115.1(12)	N(3)-C(6)-C(7)	106.4(11)	N(2)-C(3)-C(4)	107.9(8)
C(33)-C(34)-C(35)	120.0	C(5)-C(6)-C(7)	127.4(11)	C(7)-C(8)-C(9)	102.1(12)
F(9)-C(35)-C(36)	121.3(11)	C(16)-C(15)-C(37)	114.6(10)	C(4)-C(5)-C(6)	124.7(10)
F(9)-C(35)-C(34)	118.5(10)	C(16)-C(15)-C(14)	124.2(10)	C(4)-C(5)-C(25)	121.3(10)
C(36)-C(35)-C(34)	120.0	C(37)-C(15)-C(14)	121.1(10)	C(6)-C(5)-C(25)	113.8(11)
F(10)-C(36)-C(35)	118.3(11)	N(1)-C(14)-C(13)	108.1(11)	C(17)-C(18)-C(19)	105.5(11)
F(10)-C(36)-C(31)	121.6(11)	N(1)-C(14)-C(15)	128.6(10)	C(11)-C(10)-C(9)	127.6(13)
C(35)-C(36)-C(31)	120.0	C(13)-C(14)-C(15)	123.3(10)	C(11)-C(10)-C(31)	119.1(12)
C(16)-N(4)-C(19)	108.5(9)	C(18)-C(17)-C(16)	110.0(10)	C(9)-C(10)-C(31)	113.3(13)
C(6)-N(3)-C(9)	108.0(10)	N(4)-C(19)-C(20)	124.4(11)	C(12)-C(13)-C(14)	109.3(11)
C(14)-N(1)-C(11)	107.1(10)	N(4)-C(19)-C(18)	107.4(9)	C(13)-C(12)-C(11)	105.6(12)
C(1)-N(2)-C(3)	110.6(9)	C(20)-C(19)-C(18)	128.2(11)	C(10)-C(11)-N(1)	128.3(12)
N(2)-C(1)-C(20)	120.7(10)	N(3)-C(9)-C(8)	112.3(11)	C(10)-C(11)-C(12)	121.7(14)

N(1)-C(11)-C(12)	109.9(11)	C(44)-C(45)-C(46)	116.7(13)	F(15)-C(38)-C(37)	119.5(11)
C(8)-C(7)-C(6)	111.1(12)	F(18)-C(46)-C(47)	117(2)	C(39)-C(38)-C(37)	122.9(12)
C(3)-C(22)-C(23)	125.5(12)	F(18)-C(46)-C(45)	121.0(17)	C(40)-C(39)-C(38)	120.1(14)
C(48)-C(43)-C(44)	119.6(11)	C(47)-C(46)-C(45)	121.8(14)	C(40)-C(39)-F(14)	120.3(13)
C(48)-C(43)-C(20)	121.0(9)	F(17)-C(47)-C(46)	123.4(16)	C(38)-C(39)-F(14)	119.4(14)
C(44)-C(43)-C(20)	119.4(12)	F(17)-C(47)-C(48)	120.1(14)	F(4)-C(29)-C(28)	119.8(18)
F(20)-C(44)-C(43)	121.2(12)	C(46)-C(47)-C(48)	116.2(15)	F(4)-C(29)-C(30)	119.0(19)
F(20)-C(44)-C(45)	115.7(12)	C(39)-C(40)-F(13)	116.7(13)	C(28)-C(29)-C(30)	121.2(17)
C(43)-C(44)-C(45)	123.0(14)	C(39)-C(40)-C(41)	120.6(12)	F(5)-C(30)-C(29)	120.5(16)
C(42)-C(37)-C(38)	114.2(10)	F(13)-C(40)-C(41)	122.6(12)	F(5)-C(30)-C(25)	119.6(15)
C(42)-C(37)-C(15)	122.9(10)	F(12)-C(41)-C(40)	119.9(12)	C(29)-C(30)-C(25)	119.8(16)
C(38)-C(37)-C(15)	122.9(9)	F(12)-C(41)-C(42)	121.6(11)	C(37)-C(42)-F(11)	118.9(11)
C(26)-C(25)-C(30)	118.9(14)	C(40)-C(41)-C(42)	118.4(11)	C(37)-C(42)-C(41)	123.6(11)
C(26)-C(25)-C(5)	119.4(15)	F(1)-C(26)-C(25)	124.2(12)	F(11)-C(42)-C(41)	117.1(12)
C(30)-C(25)-C(5)	121.7(14)	F(1)-C(26)-C(27)	112.4(14)	F(2)-C(27)-C(28)	122.6(16)
F(16)-C(48)-C(43)	121.5(11)	C(25)-C(26)-C(27)	123.3(16)	F(2)-C(27)-C(26)	122.5(17)
F(16)-C(48)-C(47)	115.8(13)	C(29)-C(28)-F(3)	120(2)	C(28)-C(27)-C(26)	114.9(16)
C(43)-C(48)-C(47)	122.1(11)	C(29)-C(28)-C(27)	121.9(15)		
F(19)-C(45)-C(44)	126.2(15)	F(3)-C(28)-C(27)	117.8(19)		
F(19)-C(45)-C(46)	116.6(15)	F(15)-C(38)-C(39)	117.4(13)		

Table S14. Cartesian coordinates and computed total energies of molecules **2a**

```
================================================================================
                                INPUT FILE
================================================================================
NAME = i19015.inp
|  1> ## avogadro generated ORCA input file
|  2> # Advanced Mode
|  3> #
|  4> ! B3LYP SP def2-TZVP def2/J LargePrint
|  5>
|  6> * xyz 0 1
|  7>    N      24.39555      0.99742      15.97908
|  8>    N      21.25754      3.78561      17.24853
|  9>    N      25.59236      2.91139      19.61252
| 10>    N      26.53375      0.67842      17.90021
| 11>    C      22.41224      3.11538      16.76575
| 12>    C      21.61606      4.41428      18.34520
| 13>    C      23.03894      4.17351      18.65623
| 14>    C      23.76573      4.57092      19.80302
| 15>    C      24.97183      3.99495      20.19970
| 16>    C      25.76903      4.34553      21.33803
| 17>    H      25.60682      5.07220      21.92806
| 18>    C      26.79241      3.46442      21.42875
| 19>    H      27.47028      3.46005      22.09428
| 20>    C      26.67041      2.53997      20.34198
| 21>    C      27.47980      1.41827      20.07034
| 22>    C      27.41240      0.57724      18.95142
| 23>    C      28.34752     -0.52679      18.72940
| 24>    H      29.03986     -0.81252      19.31383
| 25>    C      28.03316     -1.05522      17.53687
| 26>    H      28.47630     -1.77733      17.10708
| 27>    C      26.88635     -0.31697      17.02230
| 28>    C      26.24970     -0.59696      15.80385
| 29>    C      25.08812      0.02850      15.31897
| 30>    C      24.40457     -0.22264      14.08442
| 31>    H      24.65522     -0.85868      13.42478
| 32>    C      23.33524      0.61090      14.02664
| 33>    H      22.71323      0.67072      13.31106
| 34>    C      23.30682      1.38687      15.23466
| 35>    C      22.33584      2.32648      15.60787
| 36>    C      23.49456      3.35021      17.60590
```

37>	H	24.37736	3.01774	17.49338
38>	C	20.63738	5.15809	19.16981
39>	C	20.49927	6.57039	19.05685
40>	C	19.81596	4.47572	20.06336
41>	C	21.19885	7.34490	18.00442
42>	H	20.54693	7.66436	17.34613
43>	H	21.85753	6.77127	17.56007
44>	H	21.65461	8.11118	18.41125
45>	C	19.76806	2.98936	20.16950
46>	H	20.20315	2.70706	21.00101
47>	H	20.23529	2.59205	19.40514
48>	H	18.83430	2.69197	20.17214
49>	C	23.22523	5.62778	20.71698
50>	C	22.51955	5.32608	21.87897
51>	C	22.03025	6.29837	22.72230
52>	C	22.25146	7.63154	22.41013
53>	C	22.95629	7.96136	21.27866
54>	C	23.43993	6.96418	20.45360
55>	C	28.50064	1.13797	21.13248
56>	C	28.14254	0.53279	22.32200
57>	C	29.04275	0.37256	23.36351
58>	C	30.33349	0.83108	23.22221
59>	C	30.72583	1.43251	22.05434
60>	C	29.80959	1.57418	21.02245
61>	C	26.82284	-1.66572	14.91466
62>	C	27.35268	-1.36983	13.65563
63>	C	27.81289	-2.34574	12.79209
64>	C	27.76708	-3.66866	13.17394
65>	C	27.27735	-4.00115	14.41980
66>	C	26.81037	-3.00434	15.26834
67>	C	21.14124	2.48643	14.74252
68>	C	20.79145	3.74010	14.24132
69>	C	19.68082	3.93090	13.44240
70>	C	18.86977	2.86288	13.10803
71>	C	19.19284	1.60267	13.59301
72>	C	20.29695	1.43308	14.39292
73>	F	22.27454	4.05082	22.19935
74>	F	21.34468	5.97217	23.81389
75>	F	21.77103	8.59552	23.20067
76>	F	23.15573	9.24022	20.96819
77>	F	24.13539	7.32249	19.36621
78>	F	26.88017	0.10294	22.49989

```
| 79>   F     28.65707    -0.21784    24.51227
| 80>   F     31.20187     0.68212    24.23440
| 81>   F     31.98128     1.88763    21.91531
| 82>   F     30.23419     2.16990    19.90074
| 83>   F     27.43403    -0.09857    13.24381
| 84>   F     28.32656    -2.01157    11.59987
| 85>   F     28.22501    -4.62273    12.35674
| 86>   F     27.25050    -5.27769    14.82071
| 87>   F     26.34000    -3.37659    16.46572
| 88>   F     20.52833     0.19670    14.85837
| 89>   F     18.40509     0.54969    13.30305
| 90>   F     17.79273     3.03737    12.34492
| 91>   F     19.38791     5.15236    12.97257
| 92>   F     21.56160     4.80222    14.48842
| 93>   O     19.72877     7.22049    19.83941
| 94>   O     19.03529     5.11921    20.88246
| 95>   H     19.13512     5.94604    20.77300
| 96>   H     24.63087     1.20462    16.59151
| 97>   H     25.33773     2.44196    19.00288
| 98> *
| 99>
|100>
|101>                    ****END OF INPUT****
================================================================================

                   ****************************
                   * Single Point Calculation *
                   ****************************

---------------------------------
CARTESIAN COORDINATES (ANGSTROEM)
---------------------------------
  N     24.395550    0.997420   15.979080
  N     21.257540    3.785610   17.248530
  N     25.592360    2.911390   19.612520
  N     26.533750    0.678420   17.900210
  C     22.412240    3.115380   16.765750
  C     21.616060    4.414280   18.345200
  C     23.038940    4.173510   18.656230
  C     23.765730    4.570920   19.803020
  C     24.971830    3.994950   20.199700
  C     25.769030    4.345530   21.338030
```

H	25.606820	5.072200	21.928060
C	26.792410	3.464420	21.428750
H	27.470280	3.460050	22.094280
C	26.670410	2.539970	20.341980
C	27.479800	1.418270	20.070340
C	27.412400	0.577240	18.951420
C	28.347520	-0.526790	18.729400
H	29.039860	-0.812520	19.313830
C	28.033160	-1.055220	17.536870
H	28.476300	-1.777330	17.107080
C	26.886350	-0.316970	17.022300
C	26.249700	-0.596960	15.803850
C	25.088120	0.028500	15.318970
C	24.404570	-0.222640	14.084420
H	24.655220	-0.858680	13.424780
C	23.335240	0.610900	14.026640
H	22.713230	0.670720	13.311060
C	23.306820	1.386870	15.234660
C	22.335840	2.326480	15.607870
C	23.494560	3.350210	17.605900
H	24.377360	3.017740	17.493380
C	20.637380	5.158090	19.169810
C	20.499270	6.570390	19.056850
C	19.815960	4.475720	20.063360
C	21.198850	7.344900	18.004420
H	20.546930	7.664360	17.346130
H	21.857530	6.771270	17.560070
H	21.654610	8.111180	18.411250
C	19.768060	2.989360	20.169500
H	20.203150	2.707060	21.001010
H	20.235290	2.592050	19.405140
H	18.834300	2.691970	20.172140
C	23.225230	5.627780	20.716980
C	22.519550	5.326080	21.878970
C	22.030250	6.298370	22.722300
C	22.251460	7.631540	22.410130
C	22.956290	7.961360	21.278660
C	23.439930	6.964180	20.453600
C	28.500640	1.137970	21.132480
C	28.142540	0.532790	22.322000
C	29.042750	0.372560	23.363510
C	30.333490	0.831080	23.222210

C	30.725830	1.432510	22.054340
C	29.809590	1.574180	21.022450
C	26.822840	-1.665720	14.914660
C	27.352680	-1.369830	13.655630
C	27.812890	-2.345740	12.792090
C	27.767080	-3.668660	13.173940
C	27.277350	-4.001150	14.419800
C	26.810370	-3.004340	15.268340
C	21.141240	2.486430	14.742520
C	20.791450	3.740100	14.241320
C	19.680820	3.930900	13.442400
C	18.869770	2.862880	13.108030
C	19.192840	1.602670	13.593010
C	20.296950	1.433080	14.392920
F	22.274540	4.050820	22.199350
F	21.344680	5.972170	23.813890
F	21.771030	8.595520	23.200670
F	23.155730	9.240220	20.968190
F	24.135390	7.322490	19.366210
F	26.880170	0.102940	22.499890
F	28.657070	-0.217840	24.512270
F	31.201870	0.682120	24.234400
F	31.981280	1.887630	21.915310
F	30.234190	2.169900	19.900740
F	27.434030	-0.098570	13.243810
F	28.326560	-2.011570	11.599870
F	28.225010	-4.622730	12.356740
F	27.250500	-5.277690	14.820710
F	26.340000	-3.376590	16.465720
F	20.528330	0.196700	14.858370
F	18.405090	0.549690	13.303050
F	17.792730	3.037370	12.344920
F	19.387910	5.152360	12.972570
F	21.561600	4.802220	14.488420
O	19.728770	7.220490	19.839410
O	19.035290	5.119210	20.882460
H	19.135120	5.946040	20.773000
H	24.630870	1.204620	16.591510
H	25.337730	2.441960	19.002880

TIMINGS

```
Total SCF time: 0 days 2 hours 12 min 15 sec

Total time                  ....    7935.392 sec
Sum of individual times     ....    7909.991 sec  ( 99.7%)

Fock matrix formation       ....    7741.282 sec  ( 97.6%)
  Split-RI-J                ....     498.152 sec  (  6.4% of F)
  Chain of spheres X        ....    7069.479 sec  ( 91.3% of F)
  XC integration            ....     171.809 sec  (  2.2% of F)
    Basis function eval.    ....      44.417 sec  ( 25.9% of XC)
    Density eval.           ....      54.404 sec  ( 31.7% of XC)
    XC-Functional eval.     ....       1.793 sec  (  1.0% of XC)
    XC-Potential eval.      ....      66.678 sec  ( 38.8% of XC)
Diagonalization             ....      18.972 sec  (  0.2%)
Density matrix formation    ....       3.994 sec  (  0.1%)
Population analysis         ....      79.236 sec  (  1.0%)
Initial guess               ....      24.647 sec  (  0.3%)
Orbital Transformation      ....       0.000 sec  (  0.0%)
Orbital Orthonormalization  ....       0.000 sec  (  0.0%)
DIIS solution               ....       1.607 sec  (  0.0%)
SOSCF solution              ....       4.187 sec  (  0.1%)
Grid generation             ....      36.066 sec  (  0.5%)

Maximum memory used throughout the entire SCF-calculation: 2402.0 MB

-------------------------   --------------------
FINAL SINGLE POINT ENERGY     -4242.199481367262
-------------------------   --------------------


                    ***************************************
                    *     ORCA property calculations      *
                    ***************************************

                         ---------------------
                         Active property flags
                         ---------------------
   (+) Dipole Moment


------------------------------------------------------------------------------
```

```
                    ORCA ELECTRIC PROPERTIES CALCULATION
-------------------------------------------------------------------------

Dipole Moment Calculation                  ... on
Quadrupole Moment Calculation              ... off
Polarizability Calculation                 ... off
GBWName                                    ... i19015.gbw
Electron density                           ... i19015.scfp
SHARK General Contraction Test:  Segmented basis detected
   -> Pre-screening matrix found on disk - Trying to read with NShells=889
   -> Passing Pre-screening matrix on to SHARK ...ok
   -> Leaving CheckPreScreeningMatrix
----------------------
SHARK INTEGRAL PACKAGE
----------------------

Number of atoms                             ...     91
Number of basis functions                   ...   2421
Number of shells                            ...    889
Maximum angular momentum                    ...      3
Integral batch strategy                     ... SHARK/LIBINT Hybrid
RI-J (if used) integral strategy            ... SPLIT-RIJ (Revised 2003 algorithm where possible)
Printlevel                                  ...      2
Contraction scheme used                     ... SEGMENTED contraction
Coulomb Range Separation                    ... NOT USED
Exchange Range Separation                   ... NOT USED
Finite Nucleus Model                        ... NOT USED
Auxiliary Coulomb fitting basis             ... AVAILABLE
   # of basis functions in Aux-J            ...   3851
   # of shells in Aux-J                     ...   1205
   Maximum angular momentum in Aux-J        ...      4
Auxiliary J/K fitting basis                 ... NOT available
Auxiliary Correlation fitting basis         ... NOT available
Auxiliary 'external' fitting basis          ... NOT available
Integral threshold                          ...     1.000000e-10
Primitive cut-off                           ...     1.000000e-11
Primitive pair pre-selection threshold      ...     1.000000e-11

The origin for moment calculation is the CENTER OF MASS  = (46.196316,  4.242342 34.133176)

-------------
DIPOLE MOMENT
```

	X	Y	Z
Electronic contribution:	24.33686	-19.68809	-7.85366
Nuclear contribution :	-22.88191	18.54010	7.65269
Total Dipole Moment :	1.45495	-1.14799	-0.20097

Magnitude (a.u.) : 1.86418
Magnitude (Debye) : 4.73836

Rotational spectrum

Rotational constants in cm-1: 0.000850 0.000729 0.000434
Rotational constants in MHz : 25.478442 21.867454 13.024548

Dipole components along the rotational axes:
x,y,z [a.u.] : 1.787422 0.519404 -0.102426
x,y,z [Debye]: 4.543266 1.320220 -0.260347

Timings for individual modules:

Sum of individual times ... 7940.702 sec (= 132.345 min)
GTO integral calculation ... 5.206 sec (= 0.087 min) 0.1 %
SCF iterations ... 7935.496 sec (= 132.258 min) 99.9 %
****ORCA TERMINATED NORMALLY****
TOTAL RUN TIME: 0 days 2 hours 12 minutes 21 seconds 844 msec

Table S15. Cartesian coordinates and computed total energies of molecules **4**

INPUT FILE

```
================================================================================
NAME = i19253cry.inp
|  1> ## avogadro generated ORCA input file
|  2> # Advanced Mode
|  3> #
|  4> ! B3LYP SP def2-TZVP def2/J LargePrint
|  5>
|  6>
|  7> * xyz 0 1
|  8>   C    -0.85637    0.79366    14.93579
|  9>   N    -0.84914    3.82323    18.77229
| 10>   N    -0.08152    0.03684    14.02980
| 11>   N     1.23130    1.76795    18.55947
| 12>   N    -2.25122    3.03051    16.35642
| 13>   C     0.95527   -0.39571    14.70126
| 14>   C     0.93288    0.06643    16.10401
| 15>   C     1.93071   -0.09286    17.08437
| 16>   C     2.02428    0.68661    18.24502
| 17>   C     3.01568    0.61479    19.27229
| 18>   H     3.69116   -0.04890    19.34797
| 19>   C     2.82717    1.65531    20.11976
| 20>   H     3.35950    1.85528    20.88078
| 21>   C     1.69527    2.39877    19.67607
| 22>   C     1.14559    3.55878    20.22132
| 23>   C    -0.03143    4.20198    19.80773
| 24>   C    -0.57450    5.36599    20.49423
| 25>   H    -0.18075    5.82681    21.22574
| 26>   C    -1.72930    5.66029    19.89944
| 27>   H    -2.32656    6.35999    20.13647
| 28>   C    -1.89980    4.70282    18.81584
| 29>   C    -2.98592    4.73306    17.94406
| 30>   C    -3.15739    3.95859    16.79211
| 31>   C    -4.21255    3.98536    15.84566
| 32>   H    -4.98248    4.54061    15.88316
| 33>   C    -3.92830    3.07494    14.87296
| 34>   H    -4.46958    2.88609    14.11541
| 35>   C    -2.68379    2.45313    15.18121
| 36>   C    -2.00634    1.46068    14.47084
| 37>   C    -0.26107    0.80088    16.19733
```

| 38>	H	-0.60074	1.22474	16.97671
| 39>	C	1.95693	-1.24469	14.01487
| 40>	C	1.61201	-2.54895	13.65439
| 41>	O	2.45518	-3.32121	13.02836
| 42>	C	3.17451	-0.73234	13.57761
| 43>	O	4.02937	-1.47103	12.93863
| 44>	B	3.86969	-2.94177	12.77131
| 45>	F	4.66777	-3.58236	13.67123
| 46>	F	4.16629	-3.27326	11.48103
| 47>	C	3.57466	0.69064	13.74272
| 48>	H	4.48218	0.73428	14.11001
| 49>	H	2.95259	1.13596	14.35521
| 50>	H	3.55284	1.13927	12.87171
| 51>	C	0.28910	-3.16357	13.91521
| 52>	H	-0.28545	-3.04781	13.12978
| 53>	H	-0.12554	-2.72988	14.69007
| 54>	H	0.40340	-4.11951	14.09830
| 55>	C	3.01116	-1.11543	16.89805
| 56>	C	2.69913	-2.45794	16.79780
| 57>	C	3.65815	-3.43652	16.61735
| 58>	C	4.97890	-3.08380	16.53453
| 59>	C	5.33710	-1.75704	16.62426
| 60>	C	4.35823	-0.79936	16.80429
| 61>	C	1.92838	4.18584	21.33035
| 62>	C	3.05426	4.93724	21.05061
| 63>	C	3.75804	5.59881	22.02400
| 64>	C	3.36546	5.48640	23.33320
| 65>	C	2.28695	4.70659	23.66256
| 66>	C	1.57953	4.06399	22.66455
| 67>	C	-4.10994	5.68199	18.25042
| 68>	C	-5.19615	5.31909	19.00823
| 69>	C	-6.22854	6.18234	19.29250
| 70>	C	-6.20440	7.45174	18.77820
| 71>	C	-5.13706	7.86200	18.02541
| 72>	C	-4.11589	6.97553	17.77478
| 73>	C	-2.50318	1.11799	13.10898
| 74>	C	-2.85804	-0.18125	12.75928
| 75>	C	-3.29005	-0.50358	11.50169
| 76>	C	-3.40192	0.46713	10.53611
| 77>	C	-3.05275	1.76268	10.83926
| 78>	C	-2.61997	2.06879	12.11047
| 79>	F	1.41557	-2.85339	16.87358

```
| 80>   F       3.28750      -4.71595      16.48551
| 81>   F       5.91595      -4.02149      16.35347
| 82>   F       6.61525      -1.39172      16.52315
| 83>   F       4.76609       0.47920      16.85317
| 84>   F       3.48123       5.02754      19.77759
| 85>   F       4.84009       6.32702      21.70594
| 86>   F       4.07169       6.09546      24.29605
| 87>   F       1.97266       4.54345      24.96110
| 88>   F       0.53755       3.29641      23.00839
| 89>   F      -5.27036       4.07348      19.51308
| 90>   F      -7.26628       5.78991      20.04371
| 91>   F      -7.19783       8.31392      19.04035
| 92>   F      -5.08716       9.11358      17.56842
| 93>   F      -3.07754       7.40156      17.03614
| 94>   F      -2.81139      -1.15960      13.68042
| 95>   F      -3.65068      -1.77202      11.21747
| 96>   F      -3.85907       0.16417       9.31776
| 97>   F      -3.14752       2.71996       9.90827
| 98>   F      -2.27778       3.34521      12.34905
| 99>   H      -1.46515       2.87540      16.77777
|100>   H       0.62068       2.04336      18.09595
|101> *
|102>
|103>
|104>                    ****END OF INPUT****
================================================================================

                      ****************************
                      * Single Point Calculation *
                      ****************************

---------------------------------
CARTESIAN COORDINATES (ANGSTROEM)
---------------------------------
  C     -0.856370    0.793660   14.935790
  N     -0.849140    3.823230   18.772290
  N     -0.081520    0.036840   14.029800
  N      1.231300    1.767950   18.559470
  N     -2.251220    3.030510   16.356420
  C      0.955270   -0.395710   14.701260
  C      0.932880    0.066430   16.104010
  C      1.930710   -0.092860   17.084370
```

C	2.024280	0.686610	18.245020
C	3.015680	0.614790	19.272290
H	3.691160	-0.048900	19.347970
C	2.827170	1.655310	20.119760
H	3.359500	1.855280	20.880780
C	1.695270	2.398770	19.676070
C	1.145590	3.558780	20.221320
C	-0.031430	4.201980	19.807730
C	-0.574500	5.365990	20.494230
H	-0.180750	5.826810	21.225740
C	-1.729300	5.660290	19.899440
H	-2.326560	6.359990	20.136470
C	-1.899800	4.702820	18.815840
C	-2.985920	4.733060	17.944060
C	-3.157390	3.958590	16.792110
C	-4.212550	3.985360	15.845660
H	-4.982480	4.540610	15.883160
C	-3.928300	3.074940	14.872960
H	-4.469580	2.886090	14.115410
C	-2.683790	2.453130	15.181210
C	-2.006340	1.460680	14.470840
C	-0.261070	0.800880	16.197330
H	-0.600740	1.224740	16.976710
C	1.956930	-1.244690	14.014870
C	1.612010	-2.548950	13.654390
O	2.455180	-3.321210	13.028360
C	3.174510	-0.732340	13.577610
O	4.029370	-1.471030	12.938630
B	3.869690	-2.941770	12.771310
F	4.667770	-3.582360	13.671230
F	4.166290	-3.273260	11.481030
C	3.574660	0.690640	13.742720
H	4.482180	0.734280	14.110010
H	2.952590	1.135960	14.355210
H	3.552840	1.139270	12.871710
C	0.289100	-3.163570	13.915210
H	-0.285450	-3.047810	13.129780
H	-0.125540	-2.729880	14.690070
H	0.403400	-4.119510	14.098300
C	3.011160	-1.115430	16.898050
C	2.699130	-2.457940	16.797800
C	3.658150	-3.436520	16.617350

C	4.978900	-3.083800	16.534530
C	5.337100	-1.757040	16.624260
C	4.358230	-0.799360	16.804290
C	1.928380	4.185840	21.330350
C	3.054260	4.937240	21.050610
C	3.758040	5.598810	22.024000
C	3.365460	5.486400	23.333200
C	2.286950	4.706590	23.662560
C	1.579530	4.063990	22.664550
C	-4.109940	5.681990	18.250420
C	-5.196150	5.319090	19.008230
C	-6.228540	6.182340	19.292500
C	-6.204400	7.451740	18.778200
C	-5.137060	7.862000	18.025410
C	-4.115890	6.975530	17.774780
C	-2.503180	1.117990	13.108980
C	-2.858040	-0.181250	12.759280
C	-3.290050	-0.503580	11.501690
C	-3.401920	0.467130	10.536110
C	-3.052750	1.762680	10.839260
C	-2.619970	2.068790	12.110470
F	1.415570	-2.853390	16.873580
F	3.287500	-4.715950	16.485510
F	5.915950	-4.021490	16.353470
F	6.615250	-1.391720	16.523150
F	4.766090	0.479200	16.853170
F	3.481230	5.027540	19.777590
F	4.840090	6.327020	21.705940
F	4.071690	6.095460	24.296050
F	1.972660	4.543450	24.961100
F	0.537550	3.296410	23.008390
F	-5.270360	4.073480	19.513080
F	-7.266280	5.789910	20.043710
F	-7.197830	8.313920	19.040350
F	-5.087160	9.113580	17.568420
F	-3.077540	7.401560	17.036140
F	-2.811390	-1.159600	13.680420
F	-3.650680	-1.772020	11.217470
F	-3.859070	0.164170	9.317760
F	-3.147520	2.719960	9.908270
F	-2.277780	3.345210	12.349050
H	-1.465150	2.875400	16.777770

```
  H      0.620680    2.043360   18.095950

TIMINGS
-------

Total SCF time: 0 days 1 hours 43 min 54 sec

Total time                  ....    6234.849 sec
Sum of individual times     ....    6206.995 sec  ( 99.6%)

Fock matrix formation       ....    6030.336 sec  ( 96.7%)
  Split-RI-J                ....     405.386 sec  (  6.7% of F)
  Chain of spheres X        ....    5487.519 sec  ( 91.0% of F)
  XC integration            ....     136.028 sec  (  2.3% of F)
    Basis function eval.    ....      32.008 sec  ( 23.5% of XC)
    Density eval.           ....      41.633 sec  ( 30.6% of XC)
    XC-Functional eval.     ....       1.251 sec  (  0.9% of XC)
    XC-Potential eval.      ....      55.945 sec  ( 41.1% of XC)
Diagonalization             ....      16.210 sec  (  0.3%)
Density matrix formation    ....       3.225 sec  (  0.1%)
Population analysis         ....      89.783 sec  (  1.4%)
Initial guess               ....      26.536 sec  (  0.4%)
Orbital Transformation      ....       0.000 sec  (  0.0%)
Orbital Orthonormalization  ....       0.000 sec  (  0.0%)
DIIS solution               ....       1.773 sec  (  0.0%)
SOSCF solution              ....       1.939 sec  (  0.0%)
Grid generation             ....      37.193 sec  (  0.6%)

Maximum memory used throughout the entire SCF-calculation: 2551.2 MB

-------------------------   --------------------
FINAL SINGLE POINT ENERGY     -4466.547051301894
-------------------------   --------------------


                     ***************************************
                     *     ORCA property calculations      *
                     ***************************************

                           ---------------------
                           Active property flags
                           ---------------------
```

(+) Dipole Moment

```
------------------------------------------------------------------------------
                ORCA ELECTRIC PROPERTIES CALCULATION
------------------------------------------------------------------------------

Dipole Moment Calculation                       ... on
Quadrupole Moment Calculation                   ... off
Polarizability Calculation                      ... off
GBWName                                         ... i19253cry.gbw
Electron density                                ... i19253cry.scfp
SHARK General Contraction Test:  Segmented basis detected
   -> Pre-screening matrix found on disk - Trying to read with NShells=918
   -> Passing Pre-screening matrix on to SHARK ...ok
   -> Leaving CheckPreScreeningMatrix
----------------------
SHARK INTEGRAL PACKAGE
----------------------

Number of atoms                             ...     93
Number of basis functions                   ...   2508
Number of shells                            ...    918
Maximum angular momentum                    ...      3
Integral batch strategy                     ... SHARK/LIBINT Hybrid
RI-J (if used) integral strategy            ... SPLIT-RIJ (Revised 2003 algorithm where possible)
Printlevel                                  ...      2
Contraction scheme used                     ... SEGMENTED contraction
Coulomb Range Separation                    ... NOT USED
Exchange Range Separation                   ... NOT USED
Finite Nucleus Model                        ... NOT USED
Auxiliary Coulomb fitting basis             ... AVAILABLE
   # of basis functions in Aux-J            ...   3987
   # of shells in Aux-J                     ...   1245
   Maximum angular momentum in Aux-J        ...      4
Auxiliary J/K fitting basis                 ... NOT available
Auxiliary Correlation fitting basis         ... NOT available
Auxiliary 'external' fitting basis          ... NOT available
Integral threshold                          ...     1.000000e-10
Primitive cut-off                           ...     1.000000e-11
Primitive pair pre-selection threshold      ...     1.000000e-11
```

The origin for moment calculation is the CENTER OF MASS = (0.114405, 3.554985 31.794896)

DIPOLE MOMENT

	X	Y	Z
Electronic contribution:	-1.78738	8.11283	6.01681
Nuclear contribution :	-0.01378	-5.89855	-3.77825
Total Dipole Moment :	-1.80117	2.21428	2.23856

Magnitude (a.u.) : 3.62745
Magnitude (Debye) : 9.22025

Rotational spectrum

Rotational constants in cm-1: 0.000871 0.000630 0.000402
Rotational constants in MHz : 26.106091 18.894455 12.066474

Dipole components along the rotational axes:
x,y,z [a.u.] : -3.580978 0.087036 0.572228
x,y,z [Debye]: -9.102122 0.221228 1.454488

Timings for individual modules:

Sum of individual times ... 6240.513 sec (= 104.009 min)
GTO integral calculation ... 5.544 sec (= 0.092 min) 0.1 %
SCF iterations ... 6234.969 sec (= 103.916 min) 99.9 %
****ORCA TERMINATED NORMALLY****
TOTAL RUN TIME: 0 days 1 hours 44 minutes 1 seconds 828 msec

Table S16. Cartesian coordinates and computed total energies of molecules **5**

INPUT FILE

```
================================================================================
NAME = i18568.inp
|  1> ## avogadro generated ORCA input file
|  2> # Advanced Mode
|  3> #
|  4> ! B3LYP SP def2-TZVP def2/J LargePrint
|  5> * xyz 0 1
|  6>   N     -2.14146     3.16730     5.93411
|  7>   N      1.04923     8.12294     4.80421
|  8>   N     -1.48287     5.31517     7.99871
|  9>   N     -0.86317     4.33253     3.71523
| 10>   C      0.50518     6.88438     4.55266
| 11>   C      0.74548     8.51013     6.07951
| 12>   C     -0.04896     7.45593     6.66695
| 13>   C     -0.44282     7.54796     8.02438
| 14>   C     -0.91886     6.35658     8.67938
| 15>   C     -0.70309     5.92257    10.01057
| 16>   H     -0.34558     6.44013    10.72247
| 17>   C     -1.11663     4.58577    10.07860
| 18>   H     -1.04651     4.01409    10.83410
| 19>   C     -1.65523     4.24378     8.82318
| 20>   C     -2.24809     3.01958     8.42907
| 21>   C     -2.59818     2.62387     7.11469
| 22>   C     -3.42402     1.45498     6.87338
| 23>   H     -3.85845     0.92209     7.52896
| 24>   C     -3.45486     1.27178     5.53235
| 25>   H     -3.92479     0.59744     5.05598
| 26>   C     -2.61926     2.32166     4.97296
| 27>   C     -2.28318     2.36463     3.59220
| 28>   C     -1.43842     3.29245     3.01311
| 29>   C     -1.00983     3.45466     1.66003
| 30>   H     -1.25233     2.89251     0.93361
| 31>   C     -0.19426     4.54942     1.57880
| 32>   H      0.23258     4.87699     0.79585
| 33>   C     -0.10305     5.11169     2.89678
| 34>   C      0.56688     6.27599     3.24212
| 35>   C     -0.15297     6.45981     5.70740
| 36>   H     -0.59788     5.62774     5.81789
| 37>   C      1.22156     9.59706     6.79015
```

```
| 38>   C    2.34099   10.36650    6.17217
| 39>   C    3.59862   10.64924    6.95448
| 40>   H    4.31968   10.06737    6.63525
| 41>   H    3.43718   10.47749    7.90571
| 42>   H    3.85638   11.58663    6.83093
| 43>   C   -0.21031    8.79966    8.70357
| 44>   C   -0.89626    9.17284    9.88301
| 45>   C   -0.71022   10.38637   10.48576
| 46>   C    0.18272   11.30856    9.96345
| 47>   C    0.82046   11.00520    8.76811
| 48>   C    0.65166    9.80468    8.10612
| 49>   C   -2.51808    2.03378    9.51019
| 50>   C   -3.35697    2.28729   10.57655
| 51>   C   -3.63977    1.34501   11.55590
| 52>   C   -3.07282    0.09232   11.47525
| 53>   C   -2.22107   -0.20596   10.43090
| 54>   C   -1.91641    0.76346    9.48444
| 55>   C   -2.83405    1.30552    2.68220
| 56>   C   -2.47141   -0.04126    2.81892
| 57>   C   -2.99447   -1.00775    1.99074
| 58>   C   -3.85453   -0.68743    0.97134
| 59>   C   -4.21767    0.64440    0.81025
| 60>   C   -3.71102    1.61942    1.67223
| 61>   C    1.35089    6.98885    2.20823
| 62>   C    0.75475    7.63898    1.13957
| 63>   C    1.47993    8.30006    0.18278
| 64>   C    2.87215    8.30745    0.24643
| 65>   C    3.49900    7.67656    1.28991
| 66>   C    2.74220    7.01491    2.25850
| 67>   F   -1.82718    8.37309   10.41407
| 68>   F   -1.44240   10.73344   11.57003
| 69>   F    0.34442   12.48892   10.54951
| 70>   F    1.52187   12.00025    8.20775
| 71>   F   -3.97851    3.47011   10.65046
| 72>   F   -4.46446    1.62610   12.55950
| 73>   F   -3.35494   -0.83069   12.39029
| 74>   F   -1.65346   -1.40518   10.36881
| 75>   F   -1.05947    0.43854    8.51829
| 76>   F   -1.60415   -0.39992    3.76261
| 77>   F   -2.61328   -2.29984    2.12066
| 78>   F   -4.34246   -1.59148    0.14513
| 79>   F   -5.05558    1.00112   -0.17388
```

```
| 80>   F     -4.10560      2.88061      1.47751
| 81>   F     -0.56814      7.66133      1.03436
| 82>   F      0.88116      8.93428     -0.83637
| 83>   F      3.58777      8.91998     -0.69525
| 84>   F      4.84148      7.66308      1.35631
| 85>   F      3.39529      6.40428      3.25473
| 86>   O      2.30074     10.59742      4.96145
| 87>   H     -1.75462      5.49142      7.30871
| 88>   H     -1.61035      4.18403      5.90781
| 89>   H      1.27150      8.53123      4.26276
| 90> *
| 91>
| 92>
| 93>                   ****END OF INPUT****
================================================================================

                 *****************************
                 * Single Point Calculation *
                 *****************************

---------------------------------
CARTESIAN COORDINATES (ANGSTROEM)
---------------------------------
  N    -2.141460    3.167300    5.934110
  N     1.049230    8.122940    4.804210
  N    -1.482870    5.315170    7.998710
  N    -0.863170    4.332530    3.715230
  C     0.505180    6.884380    4.552660
  C     0.745480    8.510130    6.079510
  C    -0.048960    7.455930    6.666950
  C    -0.442820    7.547960    8.024380
  C    -0.918860    6.356580    8.679380
  C    -0.703090    5.922570   10.010570
  H    -0.345580    6.440130   10.722470
  C    -1.116630    4.585770   10.078600
  H    -1.046510    4.014090   10.834100
  C    -1.655230    4.243780    8.823180
  C    -2.248090    3.019580    8.429070
  C    -2.598180    2.623870    7.114690
  C    -3.424020    1.454980    6.873380
  H    -3.858450    0.922090    7.528960
  C    -3.454860    1.271780    5.532350
```

H	-3.924790	0.597440	5.055980
C	-2.619260	2.321660	4.972960
C	-2.283180	2.364630	3.592200
C	-1.438420	3.292450	3.013110
C	-1.009830	3.454660	1.660030
H	-1.252330	2.892510	0.933610
C	-0.194260	4.549420	1.578800
H	0.232580	4.876990	0.795850
C	-0.103050	5.111690	2.896780
C	0.566880	6.275990	3.242120
C	-0.152970	6.459810	5.707400
H	-0.597880	5.627740	5.817890
C	1.221560	9.597060	6.790150
C	2.340990	10.366500	6.172170
C	3.598620	10.649240	6.954480
H	4.319680	10.067370	6.635250
H	3.437180	10.477490	7.905710
H	3.856380	11.586630	6.830930
C	-0.210310	8.799660	8.703570
C	-0.896260	9.172840	9.883010
C	-0.710220	10.386370	10.485760
C	0.182720	11.308560	9.963450
C	0.820460	11.005200	8.768110
C	0.651660	9.804680	8.106120
C	-2.518080	2.033780	9.510190
C	-3.356970	2.287290	10.576550
C	-3.639770	1.345010	11.555900
C	-3.072820	0.092320	11.475250
C	-2.221070	-0.205960	10.430900
C	-1.916410	0.763460	9.484440
C	-2.834050	1.305520	2.682200
C	-2.471410	-0.041260	2.818920
C	-2.994470	-1.007750	1.990740
C	-3.854530	-0.687430	0.971340
C	-4.217670	0.644400	0.810250
C	-3.711020	1.619420	1.672230
C	1.350890	6.988850	2.208230
C	0.754750	7.638980	1.139570
C	1.479930	8.300060	0.182780
C	2.872150	8.307450	0.246430
C	3.499000	7.676560	1.289910
C	2.742200	7.014910	2.258500

F	-1.827180	8.373090	10.414070
F	-1.442400	10.733440	11.570030
F	0.344420	12.488920	10.549510
F	1.521870	12.000250	8.207750
F	-3.978510	3.470110	10.650460
F	-4.464460	1.626100	12.559500
F	-3.354940	-0.830690	12.390290
F	-1.653460	-1.405180	10.368810
F	-1.059470	0.438540	8.518290
F	-1.604150	-0.399920	3.762610
F	-2.613280	-2.299840	2.120660
F	-4.342460	-1.591480	0.145130
F	-5.055580	1.001120	-0.173880
F	-4.105600	2.880610	1.477510
F	-0.568140	7.661330	1.034360
F	0.881160	8.934280	-0.836370
F	3.587770	8.919980	-0.695250
F	4.841480	7.663080	1.356310
F	3.395290	6.404280	3.254730
O	2.300740	10.597420	4.961450
H	-1.754620	5.491420	7.308710
H	-1.610350	4.184030	5.907810
H	1.271500	8.531230	4.262760

TIMINGS

Total SCF time: 0 days 1 hours 43 min 55 sec

Total time .... 6235.597 sec
Sum of individual times .... 6216.741 sec (99.7%)

Fock matrix formation .... 6081.041 sec (97.5%)
Split-RI-J .... 393.153 sec (6.5% of F)
Chain of spheres X .... 5536.417 sec (91.0% of F)
XC integration .... 150.085 sec (2.5% of F)
Basis function eval. .... 37.257 sec (24.8% of XC)
Density eval. .... 46.606 sec (31.1% of XC)
XC-Functional eval. .... 1.559 sec (1.0% of XC)
XC-Potential eval. .... 60.224 sec (40.1% of XC)
Diagonalization .... 17.951 sec (0.3%)
Density matrix formation .... 3.061 sec (0.0%)

```
Population analysis         ....      58.702 sec  (  0.9%)
Initial guess               ....      21.279 sec  (  0.3%)
Orbital Transformation      ....       0.000 sec  (  0.0%)
Orbital Orthonormalization  ....       0.000 sec  (  0.0%)
DIIS solution               ....       1.632 sec  (  0.0%)
SOSCF solution              ....       2.733 sec  (  0.0%)
Grid generation             ....      30.343 sec  (  0.5%)

Maximum memory used throughout the entire SCF-calculation: 2173.1 MB

-------------------------   --------------------
FINAL SINGLE POINT ENERGY     -3989.172497051828
-------------------------   --------------------


                            ***************************************
                            *     ORCA property calculations      *
                            ***************************************

                                    ---------------------
                                    Active property flags
                                    ---------------------
   (+) Dipole Moment


------------------------------------------------------------------------------
                       ORCA ELECTRIC PROPERTIES CALCULATION
------------------------------------------------------------------------------

Dipole Moment Calculation                       ... on
Quadrupole Moment Calculation                   ... off
Polarizability Calculation                      ... off
GBWName                                         ... i18568.gbw
Electron density                                ... i18568.scfp
SHARK General Contraction Test:  Segmented basis detected
   -> Pre-screening matrix found on disk - Trying to read with NShells=833
   -> Passing Pre-screening matrix on to SHARK ...ok
   -> Leaving CheckPreScreeningMatrix
----------------------
SHARK INTEGRAL PACKAGE
----------------------
```

```
Number of atoms                          ...     84
Number of basis functions                 ...   2279
Number of shells                         ...    833
Maximum angular momentum                    ...      3
Integral batch strategy                  ... SHARK/LIBINT Hybrid
RI-J (if used) integral strategy           ... SPLIT-RIJ (Revised 2003 algorithm where possible)
Printlevel                           ...      2
Contraction scheme used                   ... SEGMENTED contraction
Coulomb Range Separation                   ... NOT USED
Exchange Range Separation                  ... NOT USED
Finite Nucleus Model                     ... NOT USED
Auxiliary Coulomb fitting basis            ... AVAILABLE
   # of basis functions in Aux-J           ...   3622
   # of shells in Aux-J                  ...   1130
   Maximum angular momentum in Aux-J        ...      4
Auxiliary J/K fitting basis               ... NOT available
Auxiliary Correlation fitting basis        ... NOT available
Auxiliary 'external' fitting basis         ... NOT available
Integral threshold                       ...     1.000000e-10
Primitive cut-off                        ...     1.000000e-11
Primitive pair pre-selection threshold      ...     1.000000e-11

The origin for moment calculation is the CENTER OF MASS  = (-1.679035,  9.430528 10.846439)

-------------
DIPOLE MONENT
-------------
                                X             Y             Z
Electronic contribution:    -14.18951     -19.40484      -6.62402
Nuclear contribution   :     13.96671      18.35936       7.74828
                        -----------------------------------------
Total Dipole Moment    :     -0.22281      -1.04548       1.12425
                        -----------------------------------------
Magnitude (a.u.)       :      1.55133
Magnitude (Debye)      :      3.94316



--------------------
Rotational spectrum
--------------------
```

Rotational constants in cm-1: 0.000927 0.000764 0.000459
Rotational constants in MHz : 27.790809 22.906964 13.755721

Dipole components along the rotational axes:
x,y,z [a.u.] : -1.038069 1.065005 0.441357
x,y,z [Debye]: -2.638563 2.707028 1.121841

Timings for individual modules:

Sum of individual times ... 6240.281 sec (= 104.005 min)
GTO integral calculation ... 4.586 sec (= 0.076 min) 0.1 %
SCF iterations ... 6235.695 sec (= 103.928 min) 99.9 %
****ORCA TERMINATED NORMALLY****
TOTAL RUN TIME: 0 days 1 hours 44 minutes 1 seconds 323 msec
